# WR1065 conjugated to thiol-PEG polymers as novel anticancer prodrugs: broad spectrum efficacy, synergism, and drug resistance reversal

**DOI:** 10.3389/fonc.2023.1212604

**Published:** 2023-07-28

**Authors:** Dale M. Walker, Tsvetelina I. Lazarova, Steven W. Riesinger, Miriam C. Poirier, Terri Messier, Brian Cunniff, Vernon E. Walker

**Affiliations:** ^1^ The Burlington HC Research Group, Inc., Jericho, VT, United States; ^2^ MedChem Partners LLC, Lexington, MA, United States; ^3^ Carcinogen–DNA Interactions Section, Laboratory of Cellular Carcinogenesis and Tumor Promotion, Center for Cancer Research, National Cancer Institute, National Institutes of Health, Bethesda, MD, United States; ^4^ Department of Pathology and Laboratory Medicine, Redox Biology and Pathology Program, Larner College of Medicine, University of Vermont, Burlington, VT, United States

**Keywords:** cancer drug resistance, cancer drug sensitivity testing, cisplatin, cancer drug synergism, EGFR TKIs, normal cell safety, oxidation/reduction reactions, p53

## Abstract

The lack of anticancer agents that overcome innate/acquired drug resistance is the single biggest barrier to achieving a durable complete response to cancer therapy. To address this issue, a new drug family was developed for intracellular delivery of the bioactive aminothiol WR1065 by conjugating it to discrete thiol-PEG polymers: 4-star-PEG-S-S-WR1065 (4SP65) delivers four WR1065s/molecule and m-PEG_6_-S-S-WR1065 (1LP65) delivers one. Infrequently, WR1065 has exhibited anticancer effects when delivered via the FDA-approved cytoprotectant amifostine, which provides one WR1065/molecule extracellularly. The relative anticancer effectiveness of 4SP65, 1LP65, and amifostine was evaluated in a panel of 15 human cancer cell lines derived from seven tissues. Additional experiments assessed the capacity of 4SP65 co-treatments to potentiate the anticancer effectiveness and overcome drug resistance to cisplatin, a chemotherapeutic, or gefitinib, a tyrosine kinase inhibitor (TKI) targeting oncogenic *EGFR* mutations. The CyQUANT^®^-NF proliferation assay was used to assess cell viability after 48-h drug treatments, with the National Cancer Institute COMPARE methodology employed to characterize dose-response metrics. In normal human epithelial cells, 4SP65 or 1LP65 enhanced or inhibited cell growth but was not cytotoxic. In cancer cell lines, 4SP65 and 1LP65 induced dose-dependent cytostasis and cytolysis achieving 99% cell death at drug concentrations of 11.2 ± 1.2 µM and 126 ± 15.8 µM, respectively. Amifostine had limited cytostatic effects in 11/14 cancer cell lines and no cytolytic effects. Binary pairs of 4SP65 plus cisplatin or gefitinib increased the efficacy of each partner drug and surmounted resistance to cytolysis by cisplatin and gefitinib in relevant cancer cell lines. 4SP65 and 1LP65 were significantly more effective against *TP53*-mutant than *TP53*-wild-type cell lines, consistent with WR1065-mediated reactivation of mutant p53. Thus, 4SP65 and 1LP65 represent a unique prodrug family for innovative applications as broad-spectrum anticancer agents that target p53 and synergize with a chemotherapeutic and an EGFR-TKI to prevent or overcome drug resistance.

## Introduction

The aminothiol, 2-[(3-aminopropyl)amino]ethanethiol (WR1065), was originally developed at the Walter Reed Army Institute of Research as the major active ingredient of the FDA-approved prodrug, amifostine, to protect normal cells from radiation-induced damage (1, 2); however, WR1065 has since been reported to improve the therapeutic index of chemotherapeutic agents in some cancer patients receiving chemotherapy plus amifostine (3–7). The first reports of the anticancer effects of amifostine, when administered alone, showed that the prodrug suppressed the growth of Ehrlich ascites tumor cells (i.e., strain LP-12 of a spontaneous murine mammary adenocarcinoma) in mice (8) and significantly improved impaired hematopoiesis and slowed disease progression during a year of amifostine therapy in a human male patient with advanced stage myelodysplastic syndrome (9). Further studies of WR1065 (*in vitro*) and amifostine (*in vivo*) showed that, in a few human/rodent cancer cell types/neoplasms, WR1065 had anticancer activity alone and enhanced the effects of several classes of chemotherapeutics including platinum-based drugs (cisplatin, carboplatin), taxanes (paclitaxel), antimetabolites (5-fluorouracil), and anthracyclines (doxorubicin), as well as the tyrosine kinase inhibitor (TKI) imatinib (10–16). For example, Dai et al. (10) found that administration of either amifostine or paclitaxel inhibited the growth of human Hec50co (p53-null) endometrial tumor xenografts by ~50% compared to tumor size at the start of treatment in female nude mice (*P<*0.05), whereas cotreatment with both amifostine and paclitaxel resulted in synergistic effects overcoming resistance to cell killing, reducing tumor weight in treated animals by 96% compared to vehicle-treated controls (*P<*0.001) and, after cessation of treatment, nearly doubling the survival of animals with advanced endometrial cancer compared to treatment with paclitaxel alone. Yet, among seven meta-analyses assessing the impact of amifostine on tumor response rates in cancer patients receiving radiotherapy and/or chemotherapy (17–21), four reports found non-significant evidence of amifostine-induced beneficial effects on response types while one report showed that combination therapy with amifostine achieved significantly higher rates of complete response.

The dual effects of WR1065, that is protection of normal cells from toxic effects of radiation or chemotherapy and enhancement of the effects of chemotherapeutics in certain cancers, are paradoxical (10, 13, 15). The cytoprotective effects of WR1065 have been attributed to its activity as an antioxidant (22), but its structure and range of effects better support its activity as a reductant, nucleophilic, reactive sulfur species (23–25). Among the molecular activities reported for WR1065 (3–5, 26), the interaction of the aminothiol with subsets of proteins, transcription factors, and nucleic acids may play a crucial role in its broad range of observed effects in cancer cells including inhibition of angiogenesis, invasion, metastasis, neoplastic transformation, and occurrence of secondary cancers (5, 27–30). For example, under non-reducing conditions, WR1065 binds to the p53 protein, the p50 subunit of NF-κB, and the c-Jun subunit of AP-1, and modulates downstream events (31, 32). Binding of WR1065 to p53 results in alterations in protein conformation, modulation of p53 post-translational modifications, enhanced DNA binding, and activation of transcriptional targets including negative regulators of the cell cycle (e.g., p21^WAF1^, GADD45, 14-3-3σ), regulators of apoptosis (Bax-1, Aip-1, APO-1/Fas, Apaf-1), and genes involved in the control of intracellular redox metabolism (PIG-3, COX-2, NOS-2) (3–5, 31, 33). WR1065 is reported to target p53 by (i) enhancing p53 activity through prevention of its proteasomal degradation, (ii) increasing nuclear p53 protein levels in both normal and tumor cells, and (iii) activating a subset of mutant p53 proteins by restoring their active conformations (34–36). Thus, WR1065 works to inhibit/reduce mutant p53 orchestration of stress response mechanisms that facilitate tumor cell survival and adaptation to multiple stress conditions (37). While amifostine was developed initially to protect normal cells, decades of efforts to solve drug potency and delivery restrictions (1, 38–40) via novel packaging or second-generation phosphorothioate derivatives [(41–43) among 30 relevant reports] have yet to advance WR1065 for new disease applications.

Since mutant *TP53* is the most common driver or co-driver in various cancer types, novel therapies that reactivate mutant p53 protein and/or prevent degradation of wild-type p53 have the potential to be promising agents for use alone or in combination therapies addressing the problem of drug resistance (44–46). Despite recent advancements in new therapeutic options, the lack of anticancer agents that surmount drug resistance is the single biggest barrier to achieving improved therapy for neoplasia in general (47, 48). Primary or acquired drug resistance poses a difficult problem because (i) aggressive cancers often are heterogeneous such that not all cells respond to current therapies and (ii) resistance can occur via multiple mechanisms such as mutation induction and gene expression changes that can be triggered simultaneously (49). The current approach to this barrier is to use combinations of antineoplastic drugs with differing modes of action, but to date success has been limited (48, 50–52). Thus, the need for novel effective anticancer agents and rationally designed combinations that prevent and overcome drug resistance is imperative (50).

For this report, a novel family of prodrugs was designed to deliver WR1065 or other bioactive aminothiols intracellularly to both normal and diseased cells for new clinical uses including cancer therapy. The first two synthesized Burlington HC Research Group (BRG) prodrugs included (i) WR1065 conjugated via bioreducible disulfide bonds to each thiol-terminated arm of a 4-arm PEG-SH scaffold to yield 4-'star'-PEG-S-S-WR1065 (4SP65) and (ii) WR1065 conjugated to one end of m-PEG_6_-SH to produce m-PEG_6_-S-S-WR1065 (1LP65). Preclinical studies of 4SP65, 1LP65, and amifostine were conducted to define the relative capacity of each prodrug to inhibit cell growth or induce cell death in a panel of human cancer cell lines with differing *TP53* gene mutation status. Series of experiments also were performed to define the degree that co-treatment with 4SP65 enhances the anticancer efficacy and overcomes drug resistance (i) to cisplatin, as a representative cytotoxic chemotherapeutic, and (ii) to gefitinib, as a representative TKI targeting epidermal growth factor receptor (*EGFR*) mutations, in relevant panels of human cancer cell lines.

## Materials and methods

### Chemicals, pharmaceuticals, and reagents

WR1065 dihydrochloride [2-(3-aminopropyl)aminoethanethiol dihydrochloride, empirical formula C5H14N2S·2HCl, CAS#14653-77-1, 207.16 Da; catalog#W2020] and 4-arm-PEG-SH [pentaerythritol core, formula C(CH_2_O(CH_2_CH_2_O)nCH_2_CH_2_SH)_4_, 10,000 Da average; catalog#JKA7008] were purchased from Sigma-Aldrich (St. Louis, MO, USA). m-PEG_6_-thiol (formula C13H28O6S, CAS#441771-60-4, 312.4 Da; catalog#BP-22084) was obtained from BroadPharm (San Diego, CA, USA). Additional high purity chemicals used for synthesizing 4SP65 or 1LP65 were acquired by MedChem Partners. Amifostine was purchased from Moravek Biochemicals (Brea, CA, USA). Other pharmaceuticals or reagents including cisplatin (DDP) (Tocris Bioscience; catalog#2251/250), difluoromethylornithine (DFMO) (Selleck Chemical LLC; catalog#50-217-3144), gefitinib (LC Laboratories; catalog#G44081G), Trypan Blue, alamarBlue™ cell viability reagent (Invitrogen), and CyQUANT^®^-NF cell proliferation assay kits were obtained via ThermoFisher Scientific (Waltham, MA, USA).

### Synthesis of 4SP65 and 1LP65

4SP65 was synthesized as the first BRG prodrug using a novel multi-step scheme described in brief in Supplementary Material and in detail in a Composition of Matter patent (WO2017087668), entitled *Methods for improved protection and delivery of aminothiols and analogs thereof*. 4SP65 (average 10,532 Da), as shown below, is the abbreviation for the trifluoroacetic acid salt of the prodrug or *conjugate 7* in **Figure S1**.


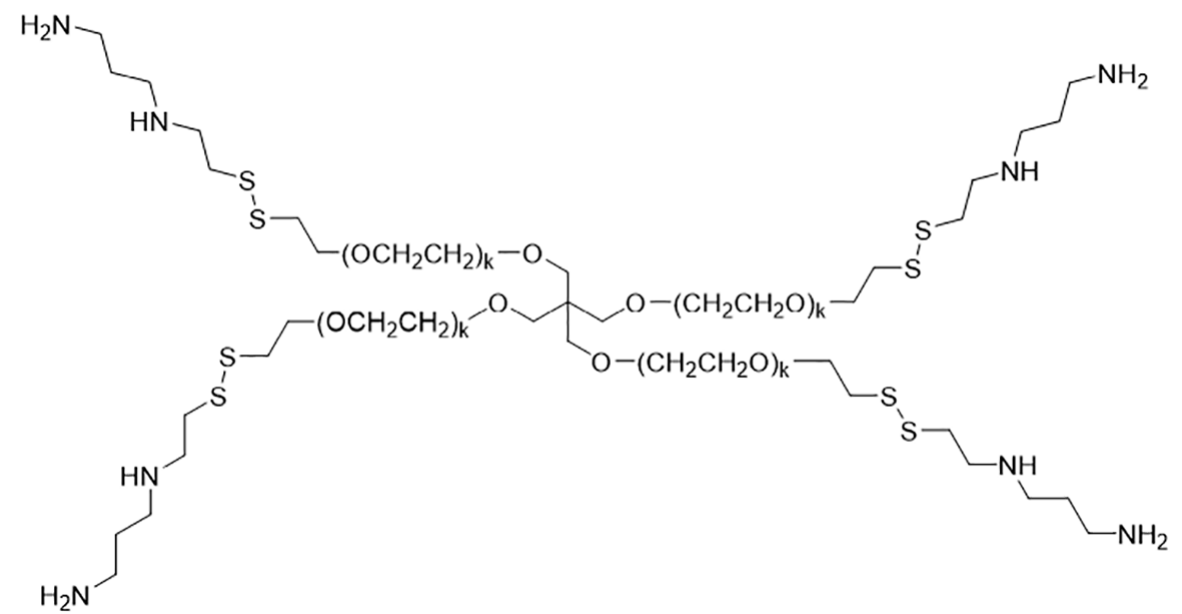


1LP65 was synthesized using the same multistep method in **Figure S1** except for the substitution of m-PEG_6_-SH shown below for *compound 5*, 


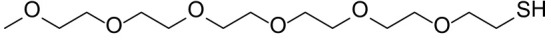


to generate Boc-protected *conjugate 6* and then the final product *conjugate 7* or 1LP65 following Boc deprotection:


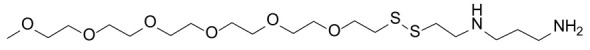


1LP65 is the abbreviation for the di-trifluoroacetic acid salt of this prodrug, weighing 483 Da once dissolved in solution (**Figures S3, S4**). **Figures S1–S4** and experimental findings related to the solubility, storage, and stability of 1LP65 and 4SP65 are presented in Supplementary Material.

### Cell lines and culture conditions

Normal human mammary epithelial cell (NHMEC) strains used to assess potential cytotoxicity of 4SP65 and 1LP65 were established from reduction mammoplasty tissue from a healthy female donor (M99005 or ‘Strain-1’) (53) or purchased from ATCC (Manassas, VA, USA; batch#70043304 or ‘Strain-2’) or Cell Applications (San Diego, CA, USA; lot#1669 or ‘Strain-3’). Human cancer cell lines including HCC38, MDA-MB-231, A549, National Cancer Institute (NCI)-H460, NCI-H1437, NCI-H1975, DU145, LNCaP, PC3, PANC1, SKOV3, and HL60, which were free of Mycoplasma, were purchased from ATCC. HMESO1, PPMMill, and TOV21G cells, obtained from Dr. Brian Cunniff, were verified to be Mycoplasma negative using the LookOut^®^ Mycoplasma PCR Detection Kit (Sigma-Aldrich) and to match previously annotated DNA fingerprints by the Vermont Integrative Genomics Resource DNA Analysis Facility.

Cell culture medium components from various vendors were obtained through ThermoFisher Scientific. Normal mammary epithelial cells were cultured in Mammary Epithelium Growth Medium (Lonza, Lexington, MA, USA), containing BulletKit™ growth supplements, at 37°C in a humidified incubator with 5% CO_2_. Mesothelioma and ovarian cancer cell lines were maintained in 50:50 DMEM (Corning, Manassas, VA, USA)/F12 medium (Lonza) supplemented with 10% (vol/vol) fetal bovine serum (FBS; Corning) and 100 U/mL penicillin-streptomycin (Corning). H1975 cells were cultured in an ATCC modified RPMI-1640 medium (Gibco, Waltham, MA, USA) with low L-glutamine, 10% FBS, and Pen-Strep. All other human cancer cell lines were grown in standard RPMI-1640 medium (Corning) supplemented with FBS and Pen-Strep.

### Assessment of 4SP65 prodrug stability, reducing capacity, and cellular uptake

To determine drug stability in tissue culture medium for short-term treatments, 4SP65 was added to medium containing alamarBlue reagent in 96-well plates and incubated in the absence of cells at 37°C for 72 h. After this incubation period, wells with medium containing alamarBlue alone or both alamarBlue and 4SP65 were assessed for fluorescence readings using a Tecan Infinite 200 PRO plate reader (San Jose, CA, USA).

To compare the utility of the alamarBlue assay and the CyQUANT^®^-NF cell proliferation assay for determining changes in cell numbers following test agent exposures, H1437 non-small cell lung cancer (NSCLC) cells were exposed for 48 h to 0 - 12.5 µM 4SP65 or to 0 - 8 µM cisplatin. After treatments, fluorescence counts were determined using alamarBlue reagent or CyQUANT^®^ dye according to manufacturers’ instructions using a plate reader. Both logarithmic and polynomial curves were fitted to each relative cell survival curve, and R^2^ values were determined using trendlines and formulas calculated with Excel software.

Since the BRG prodrugs are composed of WR1065 as one of the major constituents, assays were conducted to determine if factors reported to modulate the activity of amino acid and polyamine plasma membrane transport systems impacted the efficacy of 4SP65 or 1LP65 in selected cancer cell lines. HL60 cells were exposed to 4SP65 with up to 0.5 mM NaCl, MBA-MD-231 cells were tested in the presence and absence of medium supplemented with estradiol plus insulin (in the BulletKit™ for growth supplements for NHMECs), HCC38 cells were exposed to 4SP65 with and without 10 µM insulin added to the growth medium, and multiple cells lines were pretreated for 24 h with 1 mM DFMO prior to starting exposures to 4SP65.

### Cell viability assays

To test anticancer effectiveness of individual drugs or drug combinations, the CyQUANT^®^-NF proliferation assay was used with a plate reader (i) to generate standard curves for cell numbers for each cell line, (ii) to measure the starting cell numbers at initiation of drug treatment, and (iii) to obtain fluorescence readings from cells in 96-well plates for comparisons to a standard curve for the relevant cancer cell line and quantifying cell viability after drug treatments. DNA dyes like CyQUANT are a highly reliable method for determining the effects of anticancer agents on cell proliferation (54). Trypan blue exclusion was used to estimate cell numbers in a few pilot experiments and thereafter was applied to confirm very low viable cell numbers and cell death.

To assess the effects of 4SP65 or 1LP65 on the growth of normal epithelium, experiments were performed using NHMEC strains from differing donors (53). Strain-1 NHMECs were plated at 5,000 cells/well in 24-well plates and allowed to reach 50% confluence before treatment with 0-150 µM 4SP65 for 48 h. Cell viability was determined using a hemocytometer and trypan blue exclusion assay. Two other NHMEC strains, exposed to 0-150 µM 4SP65 or 0-500 µM 1LP65 for 48 h, were handled in the same fashion except that viability of cells in 96-well plates was determined using CyQUANT dye. Due to the short life span of these NHMECs, standard curves were not generated and fluorescence readings were used as a surrogate marker of cell number (54).

A series of experiments was conducted to determine the relative efficacy of 4SP65, 1LP65, and amifostine as single agents in the same human cancer cell lines. The effectiveness of 4SP65 was tested against 15 cancer cell lines derived from seven tissues while the efficacy of 1LP65 was examined against 11 cancer cell lines developed from four tissues. For short-term cell viability assays, 2000–5000 cells/well were plated in 96-well dishes and incubated for 24 h to allow cells to enter log-phase growth. Then starting cell numbers were determined and cells in the remaining wells were treated with 0 to ≤50 µM 4SP65 or 0 to ≤200 µM 1LP65 to characterize drug effectiveness following a single 48-h exposure. The efficacy of 0 to ≤500 µM amifostine was tested in a parallel manner in 14 cancer cell lines reported to have either medium, high, or no expression of plasma membrane-anchored alkaline phosphatase, the enzyme required for metabolism of amifostine to WR1065 and its subsequent efficient uptake into cells (55).

Additional series of experiments were performed in a parallel fashion to define the relative effectiveness of 4SP65 alone versus binary pairs of 4SP65 with cisplatin or gefitinib against selected human cancer cell lines. First, drug combination studies were conducted to compare dose-response metrics for 4SP65 alone, cisplatin alone, or both drugs combined against six human cancer cell lines derived from three tissues. Then, the dose-response metrics for 4SP65 alone, gefitinib alone, or both drugs combined were evaluated in A549 NSCLC cells. Starting cell numbers were determined 24 h after plating and remaining cells were treated with 0 to ≤50 µM 4SP65 alone, 0 to >15 µM cisplatin alone, 0 to 25 µM gefitinib alone, or with 4SP65 combined with cisplatin or gefitinib at selected ratios to measure dose-response metrics for drug effectiveness following single 48-h exposures.

### Data analyses and statistical testing

The experimental design followed guidelines for comparisons between drugs used by the NCI (56) along with recommended modifications (54). For drug combination studies, experimental design methods recommended by Chou et al. (57) were used. Data analyses used NCI methodology (56) along with those of Brooks et al. (58) to characterize concentration parameters that measure drug effectiveness following a single antagonist cancer drug treatment (Tong 2010 https://escholarship.org/uc/item/9295t422). Five metrics were calculated and used to characterize the shape of the dose-response curve following exposure to a single test agent. Using cell numbers based on fluorescence measurements and standard curves, the percentage growth was calculated at each drug concentration level. The percentage of growth inhibition was calculated as follows:


Eq. 1: [(Ti-Tz)/(C-Tz)]×100, for  concentrations  for  which  Ti >/= Tz

**Figure 1:**
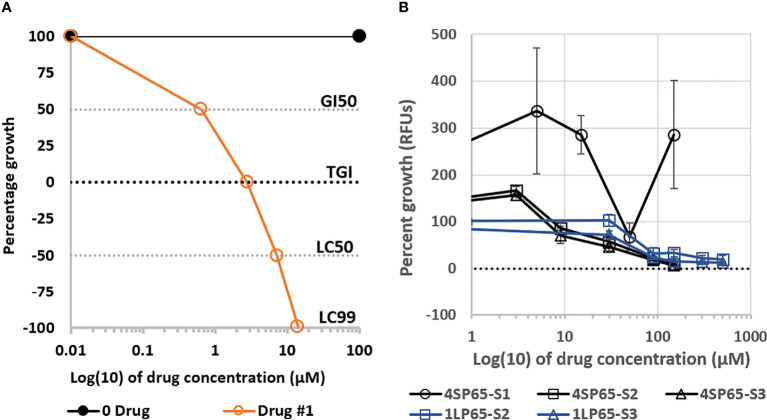
Plots **(A)** of a fitted logistic curve showing concentration parameters to measure drug effectiveness and **(B)** dose-response curves for growth of normal human mammary epithelial cells (NHMECs) treated with 4SP65 or 1LP65. In graph **(A)**, individual dose-response metrics representing the degree of effectiveness of a theoretical drug are shown on the Y-axis and defined as follows: 100 − No growth inhibition, demonstrating growth of sham-exposed control cells above starting cell numbers; GI50 − Indicates drug level that induces ‘growth inhibition of 50%’ after starting cell numbers are removed, consistent with ‘slowing of progressive disease’; TGI − Indicates drug level that induces ‘total growth inhibition’ of starting cell numbers, consistent with ‘induction of stable disease’; LC50 − Indicates drug level that induces a ‘lethal concentration of 50%’ that reduces starting cell numbers by half, consistent with ‘induction of partial disease resolution’; LC99 − Indicates drug level that induces a ‘lethal concentration of 99%’ that reduces starting cell numbers to nearly 0, consistent with ‘near complete disease resolution’. In graph **(B)**, the curves show percentage of cell growth, represented by relative fluorescence counts/units (RFUs), in strains of NHMECs exposed to 4SP65 or 1LP65, with the dotted line at ‘0’ on the Y-axis denoting relative fluorescence of starting cell numbers at the initiation of drug treatments. Tested strains of NHMECs, from three different individuals, included #M99005 (or ‘S1’), #70043304 (or ‘S2’), and #1669 (or ‘S3’). Strains were plated, incubated for ~24 h prior to initiation of treatment, and scored for growth after a 48-h exposure over a dose range of 4SP65 or 1LP65. Concentrations of 150 µM 4SP65 and 500 µM 1LP65 were the highest levels that could be tested based upon the maximum solubility of 4SP65 in aqueous medium and the need for sufficient dilution of DMSO using aqueous medium to dilute 40 mM 1LP65 in DMSO. Error bars represent SEMs of replicate experiments.

Eq. 2: [(Ti-Tz)/Tz]×100, for concentrations for which Ti < Tz

where Tz = cell numbers or fluorescence counts at time xero, C = vehicle-exposed control cell numbers at the end of the assessment time period, and Ti = test agent-exposed cell numbers or fluorescence counts at each drug concentration. The inhibitory concentration 50% (IC50) was calculated as the concentration of the drug that reduced the growth of treated cells by 50% compared to vehicle-exposed control cells. However, IC50 values do not consider the initial cell population at time zero, leading to the development of three new special concentration parameters to improve the measurement of drug effectiveness and to enhance comparisons between drugs (see **Figure 1A**). The growth inhibition of 50% (GI50) was calculated using Equation 1 = 50. The drug concentration resulting in total growth inhibition (TGI) was calculated using Equation 1 = 0. The LC50/99 was calculated using Equation 2 = -50 and Eq. 2 = -99, respectively. To define dose-response metrics in cancer cell lines, relationships between drug exposure levels and cell counts were used to calculate growth inhibition values for each exposure level, plotted on semi-log curves, and modelled using best fit regression curves and trend line formulas generated by Excel. For NHMECs, the relationships between drug exposure levels and fluorescence units were used to define dose-response metrics. The selectivity index values for individual drugs were calculated using IC50 levels in NHMECs divided by the IC50 value for each cancer cell line. Statistical comparisons of differences in individual dose-response metrics between drug treatments and cell lines were conducted using Student’s t-test, with *P*-values <0.05 considered significant.

Possible synergy between treatments with 4SP65-cisplatin or 4SP65-gefitinib drug pairs was explored further using SynergyFinder 2.0 (https://synergyfinder.fimm.fi) (59), a stand-alone web-application for interactive analysis that allows for inputting data from independent replicate experiments in order to calculate a 95% confidence interval for synergy scoring. Relative cell growth inhibition values for differing levels of each drug alone and in combination were input into SynergyFinder to generate dose-response curves for each drug alone and to produce a dose-response matrix. The dose-response matrix data then were used in generating a two-dimension heatmap and three-dimension volcano plot to visualize the distribution of areas of differing degrees of synergism and to calculate average and maximum synergy scores in selected human cancer cell lines. The expected drug combination responses were calculated using both the Highest Single Agent (HSA) model and the Zero interaction potency (ZIP) model. The HSA model, or Gaddum’s non-interaction model, assumes that the expected combination effect equals the higher individual drug effect at the dose in the combination, representing the idea that a synergistic drug combination should produce additional benefits on top of what its components can achieve alone (60). The ZIP model captures the drug interaction relationships by comparing the change in the potency (effect at a given dose level) of the dose–response curves between individual drugs and their combinations (60). Synergy scores < −10 indicate that the interaction between two drugs is likely to be antagonistic; scores from −10 to +10 indicate likely additive effects; and scores >10 indicate likely synergistic effects (59).

## Results

### Evidence of BRG prodrug stability, reducing capacity, and cellular uptake

To determine 4SP65 prodrug stability in standard growth medium, reduction of alamarBlue by 4SP65 was evaluated in a cell free system for 72 h. At this timepoint, there was no significant change in the fluorescence of alamarBlue reagent in wells containing 4SP65 compared to control wells with medium containing alamarBlue without 4SP65 (data not shown). This finding indicates that 4SP65 did not induce detectable conversion of resazurin to resorufin in the absence of cells.

A series of experiments was conducted to determine the relative capacity of alamarBlue reagent or CyQUANT^®^ dye to measure cell viability following 48-h exposures to 4SP65 (**Supplementary Figure 5**). The resulting data show that goodness-of-fit testing did not achieve R^2^ values of 0.95 or greater for logarithmic or polynomial curves fitted to data for 4SP65-exposed H1437 NSCLC cells using alamarBlue reagent. However, a R^2^ value of 0.996 was obtained for a polynomial curve fit to the relative cell survival data using CyQUANT^®^ dye in 4SP65-exposed H1437 cells. These findings demonstrate that conversion of alamarBlue reagent is non-linear in cells exposed to 4SP65 and the data are consistent with reported activity of WR1065 as a potent reducing agent and hydrogen donor in populations of normal and cancer cells (61–64). As a control, simultaneous studies were conducted using H1437 cells exposed to increasing levels of cisplatin. After removing the relative cell survival data for the two highest cisplatin concentrations where the curve flattened, curves fitted to either alamarBlue or CyQUANT^®^ fluorescence counts were linear across the change in drug levels and had R^2^ values ≥ 0.95, showing that cisplatin had negligible effects upon the reactions involved in alamarBlue conversion in cancer cells.

Since 4SP65 is larger than 500 - 600 Da, and because previous studies of WR1065 implicated the involvement of an amino acid/polyamine plasma membrane transport system in drug uptake (65), it was postulated that some kind of active transport process was involved in BRG prodrug entry into cells. Thus, a series of pilot experiments was performed to obtain qualitative data for the effect of plasma membrane transport inducers upon the effects of BRG prodrugs in differing cancer cell lines. The overall goal was to determine if repeated evidence of enhanced anticancer effects was observed in the presence versus the absence of an inducer of one or more plasma membrane transport systems. The effects of insulin alone or insulin plus estradiol, known inducers of plasma membrane transport, were evaluated (66). HCC38 and MDA-MB-231 human mammary cancer cell lines were chosen so that estradiol and insulin, reagents provided in the BulletKit™ for growth of NHMECs, could be used. The first experiment was performed by allowing HCC38 cells to reach 99% confluence and then exposing cells for 48 h to 4SP65 at 10 µM with and without 10 µM insulin. Relative cell inhibition was 3.4 ± 10.9% in cells exposed to 4SP65 alone and 42 ± 6.5% in cells exposed to 4SP65 with added insulin, representing a 12.4-fold increase in drug efficacy in growth-arrested cells. In exponentially-growing MDA-MB-231 cells exposed for 48 h to 4SP65 with or without insulin plus estradiol, drug efficacy at the TGI level was increased by nearly 4-fold with hormones (TGI = 1.2 µM) versus without hormones (TGI = 4.6 µM). In all cases, induction of cell death by BRG prodrugs occurred at lower doses in the presence of a plasma membrane transport inducer compared to in its absence.

Another series of experiments was performed to assess the impact of DFMO on the anticancer efficacy of the BRG prodrugs. DFMO is an irreversible inhibitor of ornithine decarboxylase, the rate-limiting enzyme involved with polyamine biosynthesis that also impacts polyamine transport (67). Results for A549 NSCLC cells treated with 1LP65 are illustrative of the influence of DFMO pretreatment in most human cancer cell lines tested. A549 cells were pretreated with DFMO at 1 mM for 24 h before being exposed to increasing concentrations of 1LP65 for 48 h. In A549 cells, the IC50 for 1LP65 decreased from 51 µM without DFMO pretreatment to 33.1 µM with DFMO; while the GI50 decreased from 38.6 to 17.1 µM without and with DFMO, respectively. Taken together, these sets of studies support the conclusion that both 4SP65 and 1LP65 are actively transported by one or more to-be-characterized membrane transport systems.

### 4SP65 and 1LP65 have only growth enhancement or inhibitory effects in normal mammary epithelial cells

For this report, initial evaluations of the safety of 4SP65 and 1LP65 focused on their effects upon the growth of NHMECs from different donors. In a trial experiment, Strain-1 cells were exposed for 48 h to 0, 5, 15, 50, or 150 µM 4SP65 (**Figure 1B**). Treatments with 5 and 15 µM 4SP65 greatly enhanced the growth of Strain-1 cells by 336% and 286%, respectively, compared to sham-exposed control cells. In contrast, the growth of Strain-1 cells exposed to an intermediate concentration of 50 µM 4SP65 was 68% of the control cell growth. Remarkably, treatment of Strain-1 cells with a higher concentration of 150 µM 4SP65 re-established a predominant pattern of growth enhancement by the drug. In subsequent repetitive experiments, 48-h treatments of Strain-2 and Strain-3 cells with 4SP65 induced dose-response curves with growth enhancement of 162% ± 7% at 3 µM drug and concentration-dependent decreases in growth relative to control cells of 78% ± 12%, 52% ± 8%, 21% ± 4%, and 8.7% ± 2% after respective exposures to 9, 30, 90, and 150 µM drug (**Figure 1B**). Notably, the average effect per unit dose of 4SP65 (% growth ÷exposure concentration) for 9, 30, 90, and 150 µM drug exposures was 8.7, 1.70, 0.23, and 0.058, respectively, indicating that 4SP65-mediated growth inhibitory effects reached saturation, consistent with the induction of p53-mediated cell-cycle arrest in normal cells (3, 31). Last, while IC50 values could not be predicted for Strain-1 and Strain-2 NHMECs exposed to 4SP65, an IC50 of 76 ± 14 µM was calculated for Strain-3 cells.

Treatment of Strain-2 and Strain-3 cells for 48 h with 0-500 µM 1LP65 also induced concentration-dependent decreases in cell growth, reaching 19.5% and 12.5% of control cell values, respectively, at the highest drug level (**Figure 1B**). Following exposures to 90, 150, 300, or 500 µM 1LP65, an average effect per unit dose of 2.9, 0.31, 0.06, and 0.03, respectively, was observed, providing evidence that 1LP65-mediated growth inhibition approached saturation in both strains of normal cells. However, the responses to 1LP65 exposure in these two strains of NHMECs were sufficiently different that the average IC50 values were calculated to be >500 µM in Strain-2 cells and 210 µM in Strain-3 cells, averaging >355 µM for the two normal cell strains.

### 4SP65 has broad-spectrum *in vitro* anticancer effectiveness

**Figure 2:**
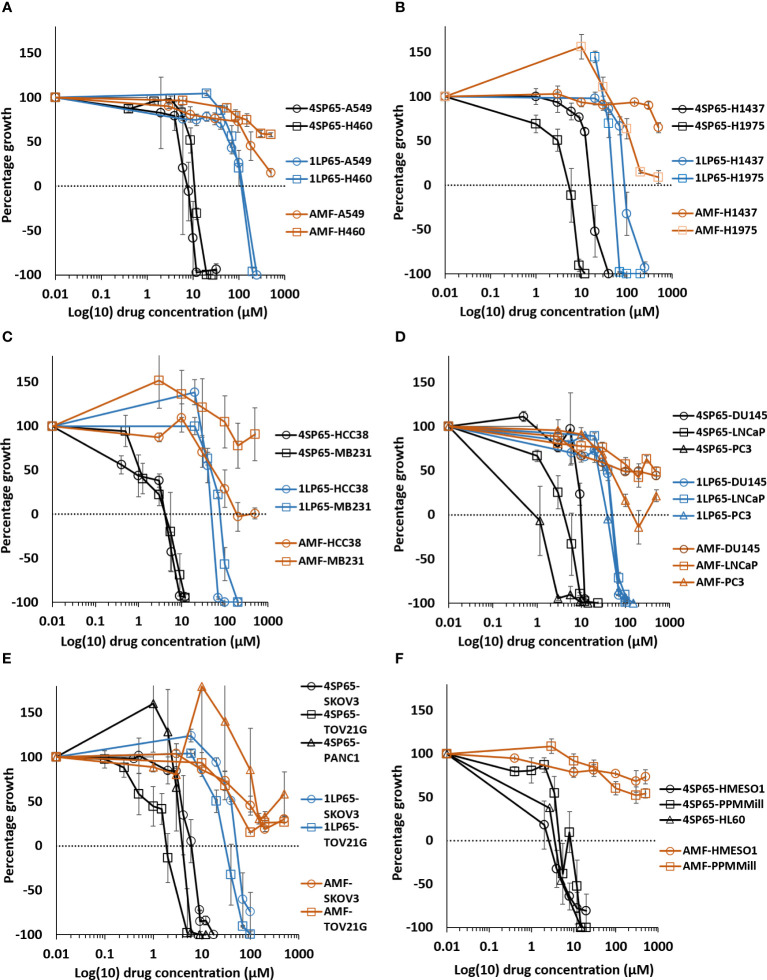
Dose-response curves for growth of human cancer cell lines exposed to 4SP65, 1LP65, or amifostine (AMF). Graphs **(A–F)** show percentage of cell growth (cytostatic growth inhibitory effects above dotted lines, total growth inhibitory effects at dotted horizontal lines, and lethal effects below dotted lines) after 48-h drug treatments, with the dotted line representing starting cell numbers. In graph C, MB231 represents MDA-MB-231 cells. Data points represent average values across 3-7 experiments for individual drugs and cell lines (see **Tables 1**–**3**); error bars represent SEs for the means of related experiments, with a minimum of four biological replicates/treatment level included in each experiment.

The BRG prodrug 4SP65, delivering four WR1065s/molecule intracellularly, yielded broad-spectrum *in vitro* anticancer effects against 15 human cancer cell lines representing four NSCLCs (A549, H460, H1437, and H1975), an acute promyelocytic leukemia (HL-60), two triple negative breast cancers (HCC38 and MDA-MB-231), three prostate cancers (DU145, LNCaP, and PC3), a serous and a clear cell ovarian carcinoma (SKOV3 and TOV21G, respectively), one pancreatic cancer (PANC1), and two malignant pleural mesotheliomas (HMESO1 and PPMMill). The results of cell proliferation assays demonstrated that, based on individual dose-response metrics listed in **Tables 1**–**3**, single 48-h exposures to 4SP65 had dose-dependent activities against all cancer cell lines tested, yielding a consistent pattern where cytostatic effects transitioned to cytocidal effects as drug levels were increased (**Figure 2**). The GI50 values show that cell growth above starting cell numbers was blocked by 50% at an average concentration of 4.1 ± 0.8 µM 4SP65, ranging from 0.7 to 10.6 µM for individual cell lines. TGI values indicate that cell growth above starting cell numbers was completely blocked at an average concentration of 6.8 ± 1.1 µM 4SP65 (range, 2.0-16.3 µM). LC50 values demonstrate that starting cell numbers were reduced by 50% at an average concentration of 9.1 ± 1.2 µM 4SP65 (range, 2.7 to 19.6 µM). LC99 values reveal that an average concentration of 11.2 ± 1.2 µM 4SP65 (range, 3.0-21.1 µM) induced near-complete cell death in all cancer cell lines, displaying a high degree of disease resolution. Moreover, if the IC50 values of >150 µM 4SP65 are used for strains 1 and 2 NHMECs and an IC50 of 76 µM 4SP65 is used for strain 3 NHMECs to calculate a selectivity index, then the average values for HCC38 and MDA-MB-231 breast cancer cells were >31 and >26, respectively, indicating that 4SP65 is a safe anticancer agent.

**Table 1:** Comparisons of dose-response metrics for 4SP65, 1LP65, and amifostine in human non-small cell lung cancer (NSCLC) and acute promyelocytic leukemia cell lines^a^.

Cell line^b^	Test drug			Dose reduction value^c^		
	4SP65	1LP65	Amifostine^d^	AMF ÷ 4SP65	AMF ÷ 1LP65	1LP65 ÷ 4SP65
A549 NSCLC cells (*TP53* WT; *CDKN2A*, *KRAS* & *STK11* mutations; EGFR protein over-expressed)						
IC50	6.8 ± 1.3	66.6 ± 7.9	261 ± 120	37	4.4	9.8
GI50	5.3 ± 1.1	52.3 ± 6.4	241 ± 130	43	5.5	9.9
TGI	8.5 ± 1.5	126 ± 3	ND	ND	ND	15
LC50	12.3 ± 2.6	155 ± 8	ND	ND	ND	13
LC99	14.9 ± 3.8	187 ± 11	ND	ND	ND	13
Experiments	n=4	n=5	n=3			
NCI-H460 NSCLC cells (*TP53* WT; *CDKN2A, KRAS, MAPK, MYC, PIK3CA*, & *STK11* mutations)						
IC50	10.7 ± 1.3	90.1 ± 16.6	ND	ND	ND	8.4
GI50	9.4 ± 1.5	77.7 ± 15.0	ND	ND	ND	8.3
TGI	14.1 ± 1.5	124 ± 13.3	ND	ND	ND	8.8
LC50	16.4 ± 0.8	155 ± 5.1	ND	ND	ND	9.5
LC99	17.7 ± 0.8	182 ± 6.7	ND	ND	ND	10
Experiments	n=6	n=3	n=3			
NCI-H1437 NSCLC cells (*TP53, JAK, MAPK, *& *NRTK3* mutations)						
IC50	12.5 ± 2.6	74.8 ± 2.1	ND	ND	ND	6.0
GI50	10.6 ± 2.0	66.2 ± 4.2	ND	ND	ND	6.5
TGI	16.3 ± 1.9	109 ± 17	ND	ND	ND	6.3
LC50	19.6 ± 0.7	157 ± 14	ND	ND	ND	8.0
LC99	21.1 ± 0.2	234 ± 16	ND	ND	ND	11
Experiments	n=7	n=4	n=4			
NCI-H1975 NSCLC cells (*EGFR* L858R & T790M mutations plus *TP53* & *PIK3CA* mutations)						
IC50	4.2 ± 1.0	52.3 ± 4.4	ND	ND	ND	13
GI50	3.1 ± 0.6	38.9 ± 2.0	130 ± 26	42	3.3	13
TGI	5.2 ± 0.9	51.8 ± 4.9	ND	ND	ND	10
LC50	7.6 ± 1.3	63.3 ± 5.2	ND	ND	ND	8.3
LC99	9.3 ± 1.1	86.8 ± 2.7	ND	ND	ND	9.3
Experiments	n =5	n=3	n=3			
Promyelocytic HL-60 leukemia cells (biallelic deletion of *TP53* null, *NRAS* mutation, *c-MYC* amplified)						
IC50	4.4 ± 0.9					
GI50	1.9 ± 0.6					
TGI	4.7 ± 0.7					
LC50	7.5 ± 2.1					
LC99	10.2 ± 3.4					
Experiments	n=4					

a Cell lines were plated, incubated for ~24 h prior to addition of test drugs, and scored after a 48-h treatment over a dose range of 4-star PEG-S-S-WR1065 (4SP65), m-PEG_6_-S-S-WR1065 (1LP65), or amifostine (AMF). The dose-response metrics measured included inhibitory concentration 50% (IC50), growth inhibitory concentration 50% (GI50), total growth inhibition (TGI), lethal concentration 50% and 99% (LC50, LC99) in each cancer cell line. Mean concentrations for each drug are given as µM, with standard errors provided for each experimental drug.

b Major oncogenic gene alterations are listed in parentheses after the name of each cancer cell line.

c Relative potency, a measurement of the fold-difference in the efficacy of the drug in the denominator compared to the drug in the numerator at a given dose-response metric; e.g., 4SP65 is 43-fold more effective than amifostine in achieving a GI50 in A549 cells.

d ND, not definable for the given dose-response metric when the highest concentration of amifostine used to treat the listed human cancer cell lines was 500 µM. Note that values above 100 µM for amifostine are listed for comparison purposes only because plasma concentrations of 100 µM amifostine and exposures of over 3 h are not readily achievable in humans or animals due to inherent dose-limiting toxicities and drug delivery restrictions (1).

**Table 2:** Comparisons of dose-response metrics for 4SP65, 1LP65, and amifostine in human mammary gland and prostate cancer cell lines^a^.

Cell line^b^	Test drug			Dose reduction value^c^		
	4SP65	1LP65	Amifostine^d^	AMF ÷ 4SP65	AMF ÷ 1LP65	1LP65 ÷ 4SP65
Mammary gland HCC38 cells (*TP53* & *PIK3CA* mutations)						
IC50	4.1 ± 0.2	47.3 ± 2.2	276 ± 92	67	5.8	12
GI50	1.3 ± 0.4	36.5 ± 2.3	78.1 ± 35.7	60	2.1	28
TGI	3.7 ± 0.3	46.9 ± 2.5	254 ± 123	69	5.4	13
LC50	6.8 ± 0.9	59.9 ± 2.9	ND	ND	ND	8.8
LC99	11.9 ± 1.6	83.9 ± 2.9	ND	ND	ND	7.1
Experiments	n=4	n=3	n=3			
Mammary gland MDA-MB-231 cells (*TP53, BRAF*, & *KRAS* mutations)						
IC50	4.7 ± 0.9	52.4 ± 4.8	ND	ND	ND	15
GI50	3.6 ± 0.8	41.8 ± 5.9	241 ± 130	151	5.8	26
TGI	6.6 ± 1.0	76.4 ± 9.2	ND	ND	ND	21
LC50	9.2 ± 1.2	110 ± 4.7	ND	ND	ND	15
LC99	11.0 ± 1.8	135 ± 6.6	ND	ND	ND	14
Experiments	n=7	n=3	n=3			
Prostate DU145 cells (*TP53, BRAF, BRCA2, EGFR, JAK2, MAPK, NRTK3, PIK3CA, *& *STK11* mutations; moderate metastatic potential)						
IC50	8.5 ± 1.0	39.8 ± 3.1	319 ± 105	38	8.0	3.5
GI50	7.5 ± 1.2	26.3 ± 3.8	126 ± 28	17	4.8	3.5
TGI	9.5 ± 0.7	52.8 ± 5.3	ND	ND	ND	5.6
LC50	11.1 ± 0.1	71.4 ± 4.3	ND	ND	ND	6.4
LC99	12.0 ± 0.1	83.5 ± 5.5	ND	ND	ND	7.0
Experiments	n=3	n=3	n=5			
Prostate LNCaP cells (*TP53 WT; ABL1/2, AKT2, ALK, BRCA1/2, EGFR, ERBB2/3/4, FLT3/4, VEGF, JAK, KIT, MAPK, MET, mTOR, MYC, NTRK3, ROS* mutations; low metastatic potential)						
IC50	3.1 ± 1.5	44.3 ± 2.8	378 ± 122	122	8.5	14
GI50	2.5 ± 1.0	28.3 ± 1.9	150 ± 47	60	5.3	11
TGI	4.7 ± 1.5	51.7 ± 3.8	ND	ND	ND	11
LC50	7.3 ± 1.8	88.8 ± 7.1	ND	ND	ND	12
LC99	10.1 ± 1.0	114 ± 8.4	ND	ND	ND	11
Experiments	n=3	n=3	n=5			
Prostate PC3 cells (*TP53* mutation; *PTEN* LOH; high metastatic potential)						
IC50	2.0 ± 0.3	37.5 ± 4.6	103 ± 15	52	2.8	19
GI50	0.7 ± 0.2	22.9 ± 1.8	53 ± 10	58	2.3	25
TGI	2.1 ± 0.5	40.5 ± 2.8	301 ± 115	143	7.4	19
LC50	4.5 ± 0.6	60.0 ± 1.1	ND	ND	ND	13
LC99	5.8 ± 0.8	86.3 ± 8.9	ND	ND	ND	15
Experiments	n=3	n=3	n=4			

a Cell lines were plated, incubated for ~24 h prior to addition of test drugs, and scored after a 48-h treatment over a dose range of 4-star PEG-S-S-WR1065 (4SP65), m-PEG_6_-S-S-WR1065 (1LP65), or amifostine (AMF). The dose-response metrics measured included inhibitory concentration 50% (IC50), growth inhibitory concentration 50% (GI50), total growth inhibition (TGI), lethal concentration 50% and 99% (LC50, LC99) in each cancer cell line. Mean concentrations for each drug are given as µM, with standard errors provided for each experimental drug.

b Major oncogenic gene alterations are listed in parentheses after the name of each cancer cell line.

c Relative potency, a measurement of the fold-difference in the efficacy of the drug in the denominator compared to the drug in the numerator at a given dose-response metric; e.g., 4SP65 is 60-fold more effective than amifostine in achieving a GI50 in HCC38 cells.

d ND, not definable for the given dose-response metric when the highest concentration of amifostine used to treat the listed human cancer cell lines was 500 µM. Note that values above 100 µM for amifostine are listed for comparison purposes only because plasma concentrations of 100 µM amifostine and exposures of over 3 h are not readily achievable in humans or animals due to inherent dose-limiting toxicities and drug delivery restrictions (1).

**Table 3:** Comparisons of dose-response metrics for 4SP65, 1LP65, and amifostine in human ovarian, pancreatic, and pleural mesothelioma cell lines^a^.

Cell line^b^	Test drug			Dose reduction value^c^		
	4SP65	1LP65	Amifostine^d^	AMF ÷ 4SP65	AMF ÷ 1LP65	1LP65 ÷ 4SP65
Ovary SKOV3 cells (*TP53, HRAS, PIK3CA* mutations)						
IC50	5.1 ± 0.9	45.5 ± 2.5	294 ± 119	58	6.5	8.9
GI50	4.0 ± 0.8	40.9 ± 0.5	81 ± 13	20	2.0	10
TGI	6.8 ± 0.9	50.3 ± 2.0	ND	ND	ND	7.4
LC50	9.2 ± 1.0	68.8 ± 1.8	ND	ND	ND	7.5
LC99	10.5 ± 1.5	98.9 ± 8.6	ND	ND	ND	9.4
Experiments	n=7	n=3	n=6			
Ovary TOV21G cells (*TP53* wild-type; hypermutated with *BRCA2*, *KRAS*, & *PIK3CA* mutations)						
IC50	1.2 ± 0.3	26.3 ± 3.6	57 ± 9	41	1.6	22
GI50	0.9 ± 0.4	21.3 ± 3.8	47 ± 10	52	2.2	24
TGI	2.0 ± 0.4	32.7 ± 6.2	ND	ND	ND	27
LC50	2.7 ± 0.4	51.3 ± 12.2	ND	ND	ND	19
LC99	3.0 ± 0.2	92.0 ± 2.8	ND	ND	ND	31
Experiments	n=3	n=3	n=3			
Pancreas PANC-1 cells (*TP53, KRAS*, & *CDKN2A/p16* mutations)						
IC50	3.7 ± 0.6		ND	ND		
GI50	2.8 ± 0.6		113 ± 39	39		
TGI	4.0 ± 0.4		ND	ND		
LC50	5.0 ± 0.3		ND	ND		
LC99	7.0 ± 0.5		ND	ND		
Experiments	n=3		n=3			
Malignant pleural mesothelioma HMESO1 cells (*TP53* mutant; biphasic histological subtype)						
IC50	2.9 ± 1.0		ND	ND		
GI50	1.3 ± 0.4		ND	ND		
TGI	2.9 ± 0.8		ND	ND		
LC50	4.8 ± 1.3		ND	ND		
LC99	7.9 ± 2.6		ND	ND		
Experiments	n=4		n=4			
Malignant pleural mesothelioma PPMMill cells (sarcomatoid histological subtype)						
IC50	7.4 ± 2.0		ND	ND		
GI50	6.0 ± 1.7		378 ± 122	39		
TGI	10.6 ± 3.0		ND	ND		
LC50	13.1 ± 2.9		ND	ND		
LC99	14.8 ± 2.9		ND	ND		
Experiments	n=6		n=3			

a Cell lines were plated, incubated for ~24 h prior to addition of test drugs, and scored after a 48-h treatment over a dose range of 4-star PEG-S-S-WR1065 (4SP65), m-PEG_6_-S-S-WR1065 (1LP65), or amifostine (AMF). The dose-response metrics measured included inhibitory concentration 50% (IC50), growth inhibitory concentration 50% (GI50), total growth inhibition (TGI), lethal concentration 50% and 99% (LC50, LC99) in each cancer cell line. Mean concentrations for each drug are given as µM, with standard errors provided for each experimental drug.

b Major oncogenic gene alterations are listed in parentheses after the name of each cancer cell line.

c Relative potency, a measurement of the fold-difference in the efficacy of the drug in the denominator compared to the drug in the numerator at a given dose-response metric; e.g., 4SP65 is 20-fold more effective than amifostine in achieving a GI50 in SKOV3 cells.

d ND, not definable for the given dose-response metric when the highest concentration of amifostine used to treat the listed human cancer cell lines was 500 µM. Note that values above 100 µM for amifostine are listed for comparison purposes only because plasma concentrations of 100 µM amifostine and exposures of over 3 h are not readily achievable in humans or animals due to inherent dose-limiting toxicities and drug delivery restrictions (1).

**Tables 1**–**3** include a list of the genes with pathogenic mutations associated with each human cancer cell line treated with 4SP65. Among eight cancer cell lines with *TP53* mutations (not including *TP53*-null cell lines), H1437 cells were much less responsive to the cytostatic/cytocidal effects of 4SP65 with significantly higher values observed for each dose-response metric compared to other *TP53* mutant cancer cell lines (*P*-values of 2 × 10^-7^ for GI50s to 6 × 10^-6^ for LC99s). A pilot study suggested that the low effectiveness of 4SP65 in *TP53*-mutant H1437 cells is a consequence of restricted active transport of the drug. This phenomenon also was observed in H460 cells and was overcome when 4SP65 was combined with cisplatin, resulting in a dramatic increase in the efficacy of 4SP65 (36-fold at the GI50 level), postulated to be due to cisplatin-induced polyamine depletion and compensatory changes in plasma membrane transport systems (68). Among the five cell lines with wild-type *TP53*, TOV21G cells were much more responsive to the cytostatic/cytocidal effects of 4SP65 with significantly lower dose-response metrics compared to other *TP53* wild-type cancer cell lines (*P*-values of 0.035 for GI50s to 0.001 for LC99s). TOV21G cells have wild-type *TP53* but exhibit a ‘hypermutator’ genotype that clearly sets them apart from other ovarian cancer cell lines including SKOV3 and high-grade serous ovarian cancer cell lines (69). If H1437 and TOV21G cells are excluded from analyses, comparisons of individual dose-response metrics for the remaining seven *TP53* mutant cancer cell lines to corresponding values for the other four *TP53* wild-type cancer cells lines show that the *TP53*-mutant cell lines are significantly more responsive than *TP53* wild-type cell lines to a 48-h 4SP65 treatment, with the *P*-values progressing from 0.03 at GI50 level cytostatic effects to 0.005 at LC99 level cytolytic effects (**Table 4**). In other words, roughly half as much 4SP65 (2.23- to 1.58-fold lower concentrations) was required to achieve equivalent responses at each dose-response metric in *TP53*-mutant cancer cell lines compared to *TP53* wild-type cell lines.

**Table 4:** Average dose-response metrics across human cancer cell lines exposed to 4SP65, 1LP65, or amifostine^a^.

Dose-response metric	4SP65				1LP65				Amifostine
	All cell lines^b^	TP53 Wild-Type^c^	TP53 Mutant^d^	P-value WT vs Mut	All cell lines^e^	TP53 Wild-Type^f^	TP53 Mutant^g^	P-value WT vs Mut	
GI50	4.1 ± 0.8	5.8 ± 1.4	2.6 ± 0.8	0.03	41.2 ± 5.4	52.7 ± 12.4	33.3 ± 3.7	0.05	47-81^h^, 149 ± 30^i^
TGI	6.8 ± 1.1	9.5 ± 2.0	4.4 ± 0.9	0.01	69.3 ± 10.3	101 ± 20.1	52.0 ± 7.3	0.03	278 ± 24^j^
LC50	9.1 ± 1.2	12.3 ± 1.9	6.7 ± 0.9	0.006	94.6 ± 12.8	133 ± 19.1	72.9 ± 9.5	0.01	Not definable^k^
LC99	11.2 ± 1.2	14.4 ± 1.6	9.1 ± 0.9	0.005	126 ± 15.8	161 ± 20.4	95.1 ± 9.9	0.01	Not definable^k^

a Cancer cell lines were plated, incubated for ~24 h prior to addition of experimental drugs, and scored after a 48-h treatment over a dose range of 4-star m-PEG_6_-S-S-WR1065 (4SP65), m-PEG_6_-S-S-WR1065 (1LP65), or amifostine. The response metrics measured included inhibitory concentration 50% (IC50), growth inhibitory 50% (GI50), total growth inhibition (TGI), lethal concentration 50% and 99% (LC50, LC99) in cancer cells. Mean concentrations for each drug are given as µM, with standard errors provided for each experimental drug tested. Additional abbreviations include: WT, wild-type; Mut, Mutant.

b Human cancer cell lines included HCC38 and MDA-MB-231 breast cancer cells; A549, H460, H1437, and H1975 non-small lung cancer cells, HMESO1 and PPMMill pleural mesothelioma cells; PANC1 pancreatic cancer cells; DU145, LNCaP, and PC3 prostate cancer cells; SKOV3 and TOV21G ovarian cancer cells; and HL60 acute promyelocytic leukemia cells.

c Included A549, H460, PPMMill, and LNCaP cell lines.

d Included HCC38, MDA-MB-231, H1975, HMESO1, PANC1, DU145, and PC3 cells.

e Included HCC38, MDA-MB-231, A549, H460, H1437, H1975, DU145, LNCaP, PC3, SKOV3, and TOV21G cells.

f Included A549, H460, and LNCaP cells.

g Included HCC38, MDA-MB-231, H1975, DU145, and PC3 cells.

h GI50 values <100 µM amifostine in 4/14 human cancer cell lines, including HCC38, PC3, SKOV3, and TOV21G cells.

i GI50 values >100 µM amifostine in 7/14 human cancer cell lines; GI50 values were not definable in 3/14 cancer cell lines.

j Amifostine concentrations >100 µM generated TGI level effects in only two cell lines, HCC38 and PC3.

k Not definable for the given dose-response metric when the highest concentration of amifostine used to treat the listed human cancer cell lines was 500 µM. Note that values above 100 µM for amifostine are listed for comparison purposes only because plasma concentrations of 100 µM amifostine and exposures of over 3 h are not readily achievable in humans or animals due to inherent dose-limiting toxicities and drug delivery restrictions (1).

4SP65 also was found to be effective against all tested human cancer cell lines having a pathogenic *KRAS* mutation in the presence or absence of a *TP53* mutation. The specific cell lines and their respective *KRAS* mutations responsive to the cytolytic effects of 4SP65 included A549 and H460 NSCLC cell lines with *KRAS* G12S and Q61H mutations, respectively, MDA-MB-231 breast cancer cells with a *KRAS* G13D mutation plus a highly expressed *TP53* R280K mutation, TOV21G ovarian cancer cells with a *KRAS* G13C mutation, and PANC1 pancreatic cancer cells with a *KRAS* G12D mutation plus a *TP53* 818G>A mutation (70–73). Three of these *KRAS* mutations (G12D, G13D, Q61H) are reported to confer either primary resistance, or to occur as “on-target” secondary mutations causing acquired resistance, to inhibitors of the most common *KRAS* mutation in NSCLC, which is *KRAS* G12C (70–72). These findings suggest the possibility that treatments combining 4SP65 with a *KRAS* ^G12C^ inhibitor can improve therapeutic outcomes given that acquired *KRAS* alterations occur in ~50% of patients developing resistance to *KRAS* G12C inhibitors (72).

Dose-response metric values for cisplatin and paclitaxel from the NCI COMPARE database allowed comparisons to the corresponding values for seven human cancer cell lines treated with 4SP65 (**Table 5**). In these cell lines, the cytostatic effects of 4SP65 largely overlapped with those of cisplatin and paclitaxel at the GI50 level. For example, the GI50 values ranged from 0.7 to 9.4 µM for 4SP65 compared to 0.1 to 5.3 µM for cisplatin in all cell lines excluding the GI50 of 22.4 µM cisplatin in MDA-MB-231 cells. In contrast, 4SP65 was 6- to >22-fold more potent than cisplatin or paclitaxel at the LC50 level, but notably the reported LC50 concentrations for cisplatin and paclitaxel are neither achievable nor safe in cancer patients (78, 79).

**Table 5:** Comparisons of dose-response metrics for 4SP65 and 1LP65 versus values reported for selected chemotherapeutic agents and PRIMA-1 or APR-246^a^.

Tissue and Cell line	Test drug				
	4SP65	1LP65	Cisplatin	Paclitaxel	PRIMA-1* or APR-246**
Lung A549 cells (*TP53* wild-type)					
IC50	6.8				29**
GI50	5.3	52	3.8	9.2	63*
TGI	8.5	126	>100	>100	
LC50	12.3	155	>100	>100	
Lung NCI-H460 cells (*TP53* wild-type)					
IC50	10.7				225**
GI50	9.4	77.7	0.6	5.8	40*
TGI	14.1	124	>100	90.4	
LC50	16.4	155	>100	>100	
Lung NCI-H1975 cells (*TP53* mutant)					
IC50	4.2	52.3			9.6**
Acute promyelocytic HL60 leukemia cells (*TP53* mutant)					
IC50	4.4				~20**
GI50	1.9		1.8	25.8	
TGI	4.7			>100	
LC50	7.5			>100	
Mammary gland MDA-MB-231 cells (*TP53* mutant)					
IC50	4.7	52.4			141*
GI50	3.6	41.8	22.4	6.4	
TGI	6.6	76.4		40.4	
LC50	9.2	110		>100	
Prostate DU145 cells (*TP53* mutant)					
GI50	7.5	26.3	2.0	1.9	
TGI	9.5	52.8		>100	
LC50	11.1	71.4			
Prostate PC3 cells (*TP53* mutant)					
GI50	0.7	22.9	5.0	36.4	
TGI	2.1	40.5		>100	
LC50	4.5	60.0		>100	
Ovary SKOV3 cells (*TP53*-null)					
IC50	5.1				16.7**
GI50	4.0	40.9	2.5	0.52	63*
TGI	6.8	50.3	>100	5.2	
LC50	9.2	68.8	>100	>100	
Pancreas PANC-1 cells (*TP53* mutant)					
IC50	3.7				66*

a Dose-response metric values for cisplatin and paclitaxel are from the NCI Database of Screening Results (https://dtp.cancer.gov/databases_tools/default.htm) and the IC50 or GI50 values for PRIMA-1 or APR-246 are from Perdrix et al. (74) (and references therein), Bykov et al. (75), Xin et al. (76), or Maslah et al. (77), with concentrations for each drug expressed as µM. * Refers to PRIMA-1 as a test drug; ** Refers to APR-246 as a test drug.

**Table 5** also shows further comparisons of GI50 values for 4SP65 with those for PRIMA-1 or APR-246 (PRIMA-1^MET^/Eprenetapopt), which are anticancer drugs that reactivate mutant p53 protein (75). Among the seven cancer cell lines available for comparisons, 4SP65 was more effective as a cytostatic agent than PRIMA-1 or APR-246 by 4.3- to 21-fold in *TP53* wild-type cells, 2.3- to 30-fold in *TP53* mutant cells, and 3.3- to 16-fold in *TP53*-null SKOV3 cells.

### 1LP65 has broad-spectrum *in vitro* anticancer effectiveness

*In vitro* treatment with 1LP65, delivering only one WR1065/molecule intracellularly, also induced broad-spectrum anticancer effects (**Figure 2** and **Tables 1** **–** **3**). Across 11 human cancer cell lines treated for 48 h with 1LP65, the dose-response metric averages were 41.2 ± 5.4 µM (range, 21.3-77.7) for GI50s, 69.3 ± 10.3 µM (range, 32.7-126) for TGIs, 94.6 ± 12.8 µM (range, 51.3-157) for LC50s, and 126 ± 15.8 µM (range, 83.5-234) for LC99s. As was the case with 4SP65, if H1437 and TOV21G cells were omitted from the analyses, comparisons of the dose-response metrics for the remaining six *TP53* mutant cancer cell lines to the corresponding values for the other three *TP53* wild-type cancer cell lines showed that the *TP53*-mutated cell lines were significantly more responsive (by ~1.8-fold lower concentrations of drug) than *TP53* wild-type cell lines to 1LP65 treatment (**Table 4**).

**Table 5** shows comparisons between dose-response metrics for 1LP65, cisplatin, and paclitaxel in six human cancer cell lines treated for 48 h. At the GI50 level, the doses of cisplatin that achieved cytostatic effects were ~5- to 14-fold lower than those of 1LP65 in four cell lines and 130-fold lower in H460 cells. MDA-MB-231 cells were the exception, where a GI50 of 22.4 µM cisplatin is toxic in cancer patients (78) while a GI50 of 41.8 µM 1LP65 is safe based upon the NHMEC studies. Similarly, at the GI50 level, the cytostatic effects of paclitaxel were achieved at approximately 6- to 14-fold lower doses than 1LP65 in four cell lines and 77-fold lower in SKOV3 cells. PC3 cells were the exception, where a GI50 of 36.4 µM paclitaxel is highly toxic *in vivo* (79), while a GI50 of 22.9 µM 1LP65 likely is safe and clinically achievable. Notably, the levels of cisplatin and paclitaxel required for LC50 level cytolytic effects in these cancer cell lines occur at drug concentrations that are neither safe nor achievable in cancer patients (78, 79). While *in vitro* studies indicate that 500 µM 1LP65 is not cytotoxic in NHMECs, pharmacokinetic studies need to be conducted to determine if concentrations of 60-155 µM 1LP65 that induce LC50 level cytolytic effects across the 11 cancer cell lines tested can be achieved safely *in vivo*.

### Amifostine has limited cytostatic and lacks cytolytic effectiveness in human cancer cell lines compared to 4SP65 and 1LP65

The potential cytostatic and cytolytic effects of amifostine, delivering one WR1065/molecule extracellularly, were assessed in the same human cancer cell lines to enable direct comparisons to the anticancer effectiveness of 4SP65 and 1LP65 (**Figure 2** and **Tables 1** **–** **4**). Although plasma concentrations of 100 µM amifostine and exposures of over 3 h are not readily achievable in humans or animals due to inherent drug delivery restrictions and dose-limiting side-effects (1), for comparison purposes, cell lines were exposed for 48 h to 0-500 µM amifostine. Among 14 cancer cell lines, GI50 values of 47-81 µM amifostine were attained in four cell lines (HCC38, PC3, SKOV3, and TOV21G) reported to exhibit moderate/high expression of alkaline phosphatase (80, 81), GI50 values of 113-378 µM were achieved in seven cell lines, and exposures up to 500 µM failed to induce cytostatic effects in three cell lines. Amifostine levels >100 µM induced TGI level effects in only two cell lines, HCC38 and PC3. Cytolytic effects were not found in any cell line even at exposure levels of 500 µM amifostine. At high concentrations of 200-500 µM amifostine, the dose-response curves were U-shaped for 6/14 cell lines, indicating that exposure to higher drug levels did not induce a greater effect. In the other cell lines, the dose-response curves flattened between 200-500 µM amifostine, without achieving greater growth inhibition. In contrast, dose-response curves for 4SP65 and 1LP65 did not display a U-shape at any concentration range in cancer cell lines, and did not flatten until >97% cytolytic effects were achieved.

**Figure 3:**
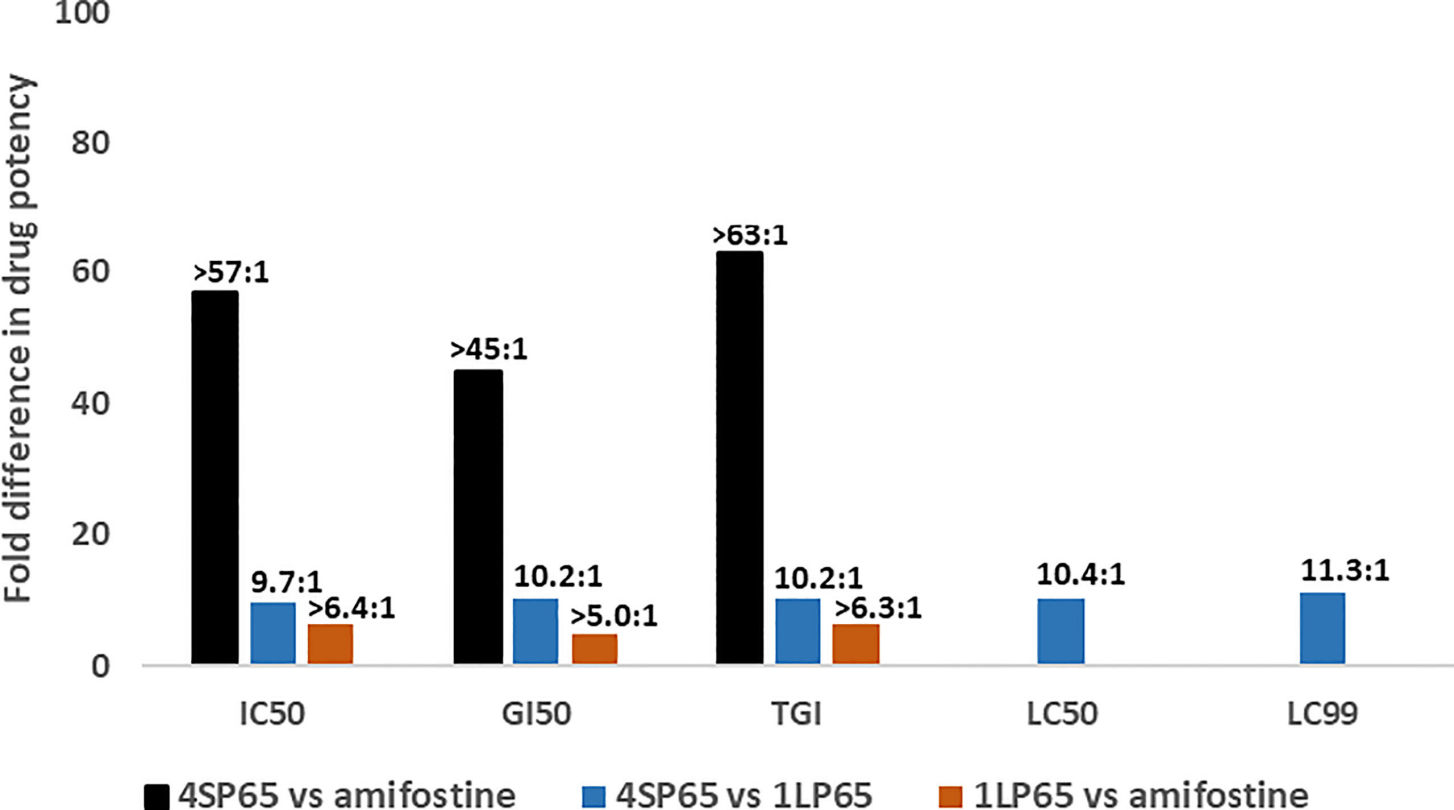
Relative drug potency ratios based on the dose-response metrics for 4SP65, 1LP65, and amifostine across a panel of human cancer cell lines. For each dose-response metric, the values obtained after a 48-hour treatment of 11 human cancer cell lines with each individual drug (see data in **Tables 1**–**3**) were first averaged and then fold-differences in drug potency were calculated as ratios for 4SP65 compared to amifostine, 4SP65 compared to 1LP65, and 1LP65 compared to amifostine. IC50, GI50, and TGI values were measurable in only nine or fewer cancer cell lines following maximum exposures of 500 µM amifostine, and thus the solid bars represent minimal fold differences in drug potency for cytostatic effects between 4SP65 or 1LP65 compared to amifostine. Maximum exposures to 500 µM amifostine did not yield LC50 or LC99 values in any cancer cell line, so fold differences in drug potency for cytolytic effects between 4SP65 or 1LP65 versus amifostine cannot be calculated.

**Figure 3** shows the results of the estimates of the relative anticancer potency of 4SP65, 1LP65, and amifostine based on the averages of individual dose-response metrics for each compound across a panel of cancer cell lines. First, 4SP65 was 45- to 63-fold (averaging 55-fold) more potent than amifostine as a cytostatic agent, far exceeding the difference predicted solely by the ratio of four versus one WR1065/molecule in 4SP65 compared to amifostine. Second, 1LP65 was 5.0- to 6.4-fold (averaging 5.9-fold) more potent than amifostine as a cytostatic agent even though both drugs delivered a single WR1065 molecule. If the analysis of the relative cytostatic potency between 4SP65 or 1LP65 versus amifostine is restricted to comparisons where amifostine achieved a GI50 value at <100 µM drug in four cell lines, then the average differences in potency remain significant (*P <*0.007) with a 38-fold greater effect for 4SP65 (average GI50 = 1.7 µM) than amifostine (average GI50 = 64.8 µM) and a 2.1-fold greater effect for 1LP65 (average GI50 = 30.4 µM) than amifostine. Third, 4SP65 was 9.7- to 11.3-fold (averaging 10.4-fold) more potent than 1LP65 depending on the dose-response metric, with the difference increasing as drug levels were raised to achieve LC50 or LC99 effects. Thus, the potency of 4SP65 was ~2.5-fold greater than that predicted by the ratio of four WR1065s/molecule in 4SP65 versus one in 1LP65. Last, the relative potency of 4SP65 and 1LP65 as cytocidal agents compared to amifostine is immeasurable because the latter drug did not induce cytolytic effects in any cancer cell line.

### 4SP65 enhances anticancer effectiveness and overcomes drug resistance in combination with cisplatin

A series of experiments was conducted to assess the relative drug efficacy of 4SP65 and cisplatin as single agents or in combination against two NSCLC, two pleural mesothelioma, and two ovarian cancer cell lines. For these experiments, a human cancer cell line was considered resistant to cisplatin when the amount of drug required to achieve cytolytic effects in cell culture was ≥15 µM, which exceeds reported peak plasma levels (C_max_) of free drug averaging from 3.8 ± 1.7 µM to 7.5 ± 6.8 µM cisplatin during routine 1 h IV infusions to differing sets of cancer patients (78, 82). 4SP65 was deemed to overcome resistance to cisplatin when the two drugs combined resulted in a significant fold-increase in the level of cytocidal effects for cisplatin at concentrations below 15 µM and the efficacy of 4SP65 was increased as well.

**Figure 4:**
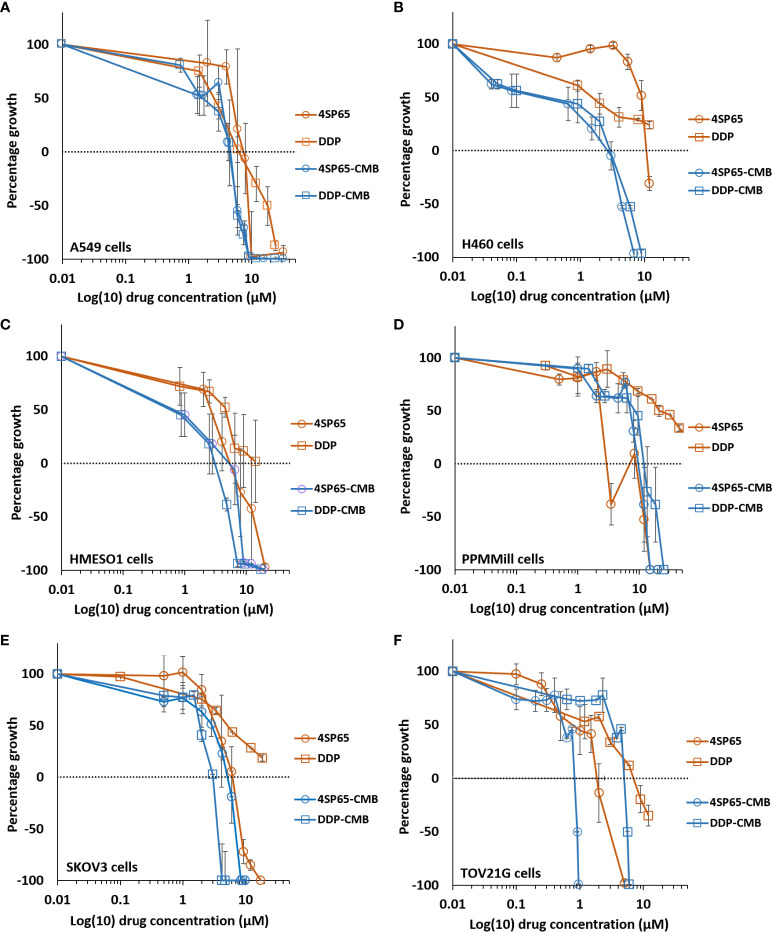
Dose-response curves for growth of human cancer cell lines exposed to 4SP65, cisplatin (DDP), or both drugs. Graphs **(A–F)** show percentage of cell growth (cytostatic growth inhibitory effects above dotted lines, total growth inhibitory effects at dotted horizontal lines, and lethal effects below dotted lines) after 48-h treatments, with the dotted line representing starting cell numbers. In the binary drug pair, the concentrations of the individual drugs in the combination are plotted as 4SP65-CMB and DDP-CMB. Data points represent average values across 3 or 4 experiments for single or combined drug treatment of each cell line (see **Table 6**); error bars represent SEs for the means of related experiments, with a minimum of four biological replicates/treatment level included in each experiment.

For each cancer cell line, **Figures 4A–F** shows the dose-response curves for cell growth after 48-h treatments with 4SP65, cisplatin, or both drugs, while **Table 6** lists the corresponding dose-response metrics, the fold-increase in efficacy for each drug in the combination (with *P*-values indicated where increases are significant), and the synergy scores for the combination. For H460, HMESO1, PPMMill, and SKOV3 cells, cisplatin alone induced only cytostatic effects, consistent with slowing of progressive disease. In contrast, 4SP65-cisplatin combined resulted in cytolytic effects at plasma-equivalent levels of cisplatin with significant dose-dependent gains of both cytostatic and cytolytic activities by 1.7- to 6.9-fold for cisplatin in all four cancer cell lines. Co-treatment with 4SP65-cisplatin also resulted in significant fold increases in the cytostatic and cytolytic activities by 1.3- to 36-fold in H460 and SKOV3 cells, but the modest gains of effect for 4SP65 (1.1- to 1.3-fold) in HMESO1 and PPMMill cells were not significant. In A549 cells and TOV21G cells, treatment with cisplatin alone achieved a LC50 but not a LC99 at <15 µM drug. In A549 cells, combined treatment with 4SP65-cisplatin resulted in significant gains of both cytostatic and cytolytic activities by 1.9- to 2.4-fold for 4SP65 and 1.7- to 3.5-fold for cisplatin to achieve a LC99 at 8 µM 4SP65 plus 7 µM cisplatin. In TOV21G cells, co-treatment with 4SP65-cisplatin also yielded significant gains in total growth inhibition and cytolytic effects by 2.4- to 3.2-fold for 4SP65 and 1.3- to 2.9-fold for cisplatin, reducing the LC99 values of 3.0 µM for 4SP65 alone and 17.2 µM for cisplatin alone to 0.95 µM 4SP65 plus 5.9 µM cisplatin when combined.

**Table 6:** Dose response metrics for 4SP65 alone, cisplatin alone, and 4SP65 and cisplatin combined (CMB) in drug-exposed human cancer cell lines^a^.

Experiments using 4SP65 and/or cisplatin (DDP)						
Cell line	4SP65	4SP65-CMB	DDP	DDP-CMB	Fold increase in efficacy	
					4SP65 ÷ 4SP65-CMB	DDP ÷ DDP-CMB
A549 NSCLC cells (n = 4 experiments; drug ratio was 50% 4SP65 [µM]:50% DDP [µM]						
IC50	6.8 ± 1.3	3.1 ± 0.5	4.7 ± 0.8	2.8 ± 0.7	2.2*	1.7*
GI50	5.4 ± 2.5	2.3 ± 0.5	3.5 ± 0.7	2.0 ± 0.6	2.4*	2.3*
TGI	8.3 ± 1.5	4.3 ± 0.5	8.2 ± 1.0	3.8 ± 0.7	1.9*	1.8**
LC50	12.1 ± 2.7	6.5 ± 0.6	13.6 ± 2.2	5.7 ± 0.8	1.9*	2.0**
LC99	14.9 ± 3.8	8.0 ± 0.6	24.4 ± 2.6	7.0 ± 0.8	1.9*	3.5***
Average HSA (and ZIP) synergy scores = 23.6 (19.8); Most synergistic area scores = 29.8 (25.8)^b^						
NCI-H460 NSCLC cells (n = 4 experiments; drug ratio was 43% 4SP65 [µM]:57% DDP [µM])						
IC50	13.3 ± 1.0	0.67 ± 0.04	4.0 ± 0.1	0.88 ± 0.53	20**	4.6***
GI50	12.3 ± 1.1	0.34 ± 0.07	3.1 ± 0.1	0.45 ± 0.09	36**	6.9***
TGI	16.9 ± 0.6	2.3 ± 0.3	ND (>15)	2.6 ± 0.9	7.4^c^	>5.8 *GoE*
LC50	17.6 ± 0.5	3.3 ± 0.2	ND (>15)	4.4 ± 0.3	5.3^c^	>3.4 *GoE*
LC99	18.2 ± 0.4	4.2 ± 0.3	ND (>15)	5.6 ± 0.4	4.3^c^	>2.7 *GoE*
Average HSA (and ZIP) synergy scores = 36.4 (31.9); Most synergistic area scores = 57.5 (54.6)^b^						
HMESO1 mesothelioma cells (n = 3 experiments; drug ratio was 50% 4SP65 [µM]:50% DDP [µM])						
IC50	4.7 ± 0.2	4.4 ± 0.7	8.6 ± 1.1	3.0 ± 0.3	1.1 (NS)	2.9*
GI50	2.0 ± 0.3	1.3 ± 0.3	3.0 ± 0.4	0.8 ± 0.2	1.5 (NS)	3.9*
TGI	4.3 ± 0.2	3.4 ± 0.2	6.7 ± 0.7	2.6 ± 0.3	1.3 (NS)	2.6**
LC50	7.0 ± 0.1	7.0 ± 1.0	ND (>15)	4.8 ± 0.3	1.0 (NS)	>3.1 *GoE*
LC99	12.4 ± 0.1	10.9 ± 0.6	ND (>15)	8.9 ± 0.4	1.1 (NS)	>1.7 *GoE*
Average HSA (and ZIP) synergy scores = 13.1 (14.3); Most synergistic area scores = 36.1 (31.9)^b^						
PPMMill mesothelioma cells (n = 4 experiments; drug ratio was 56% 4SP65 [µM]:44% DDP [µM])						
IC50	10.0 ± 1.9	7.7 ± 1.5	40.5 ± 8.1	11.6 ± 2.3	1.3 (NS)	3.5**
GI50	8.4 ± 1.5	6.3 ± 1.4	27.2 ± 3.4	9.5 ± 2.1	1.3 (NS)	5.5**
TGI	14.1 ± 3.3	10.8 ± 1.8	ND (>100)	16.3 ± 2.7	1.3 (NS)	>6.1 *GoE*
LC50	16.2 ± 3.2	12.9 ± 1.3	ND (>100)	19.3 ± 1.9	1.3 (NS)	>5.2 *GoE*
LC99	17.6 ± 3.5	13.9 ± 1.2	ND (>100)	20.9 ± 1.8	1.3 (NS)	>4.8 *GoE*
Average HSA (and ZIP) synergy scores = 5.8 (10.3); Most synergistic area scores = 20.8 (13.3)^b^						
SKOV3 ovarian cancer cells (n = 4 experiments; drug ratio was 43% 4SP65 [µM]:57% DDP [µM])						
IC50	5.2 ± 1.0	2.9 ± 0.6	6.7 ± 0.6	2.6 ± 0.3	1.8**	3.2**
GI50	4.3 ± 1.0	2.7 ± 0.6	5.3 ± 0.4	2.3 ± 0.2	1.5**	1.7**
TGI	7.3 ± 0.8	4.9 ± 0.6	ND (>21)	4.8 ± 0.9	1.5*	>4.4 *GoE*
LC50	9.7 ± 0.5	7.4 ± 0.3	ND (>21)	8.4 ± 2.8	1.3**	>2.5 *GoE*
LC99	10.0 ± 0.8	7.7 ± 0.1	ND (>21)	12.9 ± 3.8	1.3*	>1.6 *GoE*
Average HSA (and ZIP) synergy scores = 18.2 (19.8); Most synergistic area scores = 42.1 (41.6)^b^						
TOV21G ovarian cancer cells (n = 3 experiments; drug ratio was 14% 4SP65 [µM]:86% DDP [µM])						
IC50	1.2 ± 0.3	0.62 ± 0.01	3.0 ± 0.3	3.8 ± 0.1	1.9 (NS)	0.8 (NS)
GI50	0.93 ± 0.37	0.41 ± 0.13	2.2 ± 0.4	2.5 ± 0.8	2.3 (NS)	0.9 (NS)
TGI	2.0 ± 0.4	0.85 ± 0.01	6.7 ± 0.3	5.2 ± 0.1	2.4*	1.3*
LC50	2.7 ± 0.1	0.91 ± 0.01	13.2 ± 2.2	5.6 ± 0.1	3.0***	2.4*
LC99	3.0 ± 0.2	0.95 ± 0.02	17.2 ± 5.2	5.9 ± 0.1	3.2***	2.9*
Average HSA (and ZIP) synergy scores = 18.2 (14.8); Most synergistic area scores = 26.4 (20.6)^b^						

a Cell lines were plated in 96-well microtiter dishes, incubated for ~24 h prior to addition of experimental drugs, and then treated over a dose range for 48 h before collecting data and calculating values for inhibitory concentration 50% (IC50), growth inhibitor concentration 50% (GI50), total growth inhibitory concentration (TGI), lethal concentration 50% (LC50) and 99% (LC99) as set forth by the NCI (56, 83). All units are µM, with means and SEMs. 4SP65-CMB and DDP-CMB refer to the amounts of each drug in combination at each dose-response metric; ND, not definable, with following numbers in parentheses indicating the highest concentration of drug tested in µM for a given cancer cell line; GoE, gain of effect; NS, non-significant.

b Synergy score data obtained via SynergyFinder (59); scores <-10 likely antagonistic, -10 to +10 likely additive, and >10 likely synergistic on a scale of 60.

* *P* <0.05, ** *P* ≤0.01, *** *P* ≤0.001

### Visual analytics of synergy for 4SP65 and cisplatin combined

**Figure 5:**
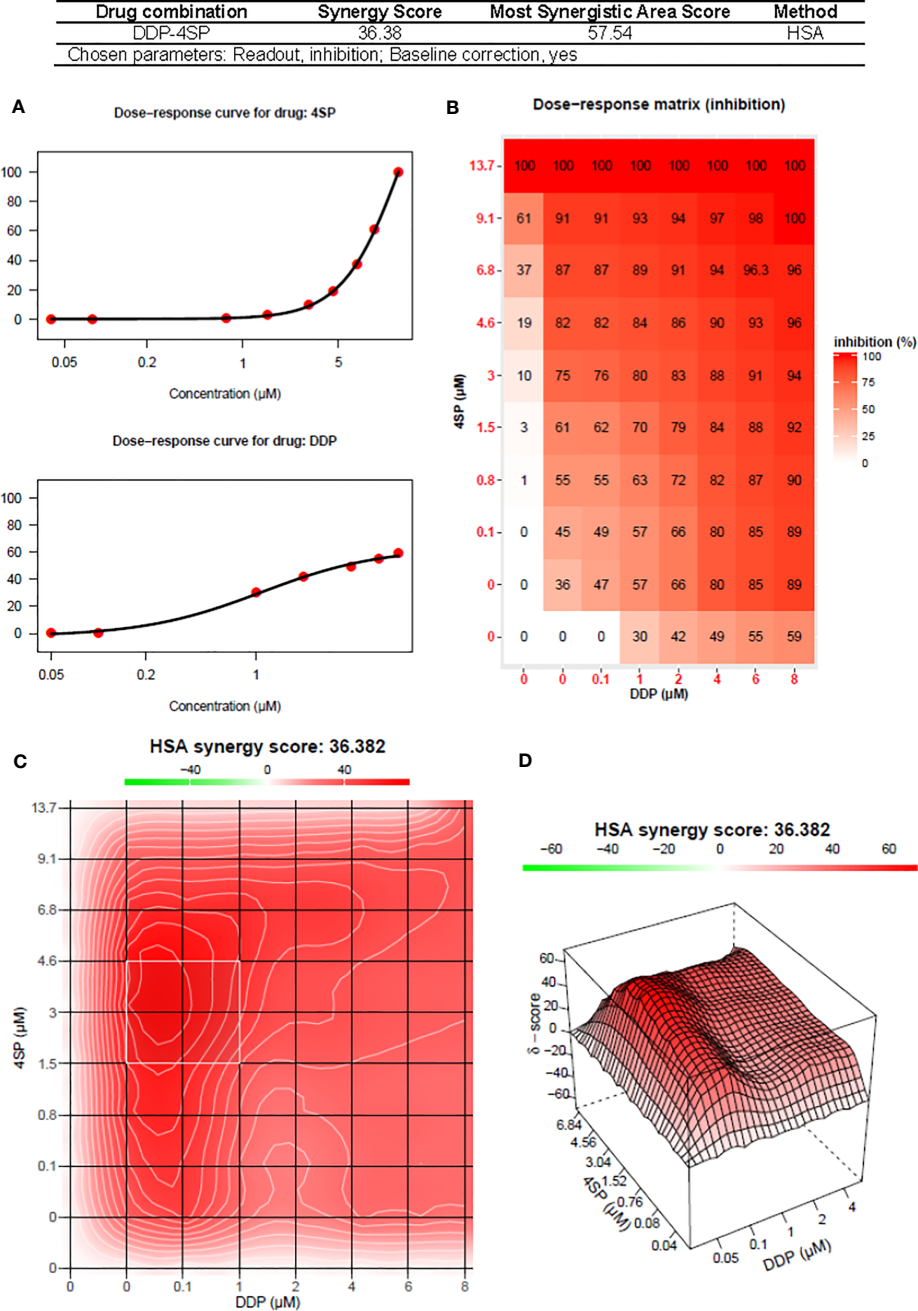
Synergy report for H460 NSCLC cells treated with cisplatin (DDP) alone, 4SP65 (4SP) alone, or both drugs for 48 h. The report was generated using SynergyFinder 2.0 (https://synergyfinder.fimm.fi) (59). Panels include **(A)** the dose-response curves for 4SP65 alone (upper graph) and cisplatin alone (lower graph), **(B)** the dose-response matrix for growth inhibition by each drug alone and in combination, and **(C)** a two-dimension heat map and **(D)** a three-dimension volcano plot showing the distribution of areas of differing degrees of synergism.

The nature of the combined cytostatic/cytocidal effects of 4SP65-cisplatin was surveyed further via synergy reports where the upper limits for cisplatin were set at 15 µM to focus on responses at *in vitro* treatment levels corresponding to reported peak plasma levels of free drug. The highest average HSA synergy score of 36.4 on a scale of 60 was observed in H460 cells, where the potentiation of 4SP65-cisplatin combined was most prominent in the low-dose region of both drugs as shown in the heat map and in a ridge of high synergy score in the volcano plot of 45 to 57 at concentrations of <1 µM cisplatin combined with 1.5 to 4.6 µM 4SP65 (**Figure 5**). A similar pattern with an average HSA synergy score of 23.6 was observed in A549 cells with, for example, treatment with 3-6 µM cisplatin and 4-8 µM 4SP65 yielding ~80-90% reduction in starting cell numbers (**Supplementary Figure 6A**) while 13.6 µM cisplatin alone reduced starting cell numbers by only 50% (**Table 6**). The average HSA synergy score in SKOV3 cells was 18.2 with a ridge of high energy score approaching 42 in the volcano plot at concentrations of 4.5 to 6.0 µM cisplatin and 6 to 9 µM 4SP65 associated with 95-100% reduction in starting cell numbers (**Supplementary Figure 6E**). In contrast, corresponding levels of cisplatin alone were not cytolytic in SKOV3 cells. TOV21G cells were highly sensitive to cytolytic effects of 4SP65 so that, while the average synergy score for the combination treatment was 18.2, a 48-h exposure to ~6.0 µM cisplatin and 1 µM 4SP65 resulted in near-complete cell killing in the region of the most synergistic area score of 26.4 (**Supplementary Figure 6F**). On the other hand, cisplatin alone reduced starting numbers by only 50% (LC50) at 13.2 µM cisplatin in TOV21G cells. 4SP65-cisplatin combined was least effective against HMESO1 and PPMMill cells, with weak potentiation or additive effects driven primarily by significant increases in the cytolytic effects of cisplatin in the combination, effects that were not achieved by plasma-equivalent levels of cisplatin alone (**Table 6** and **Supplementary Figures 6C, D**).

### 4SP65 enhances anticancer effectiveness and overcomes drug resistance in combination with gefitinib in A549 cells with innate resistance to EGFR-TKIs

**Figure 6:**
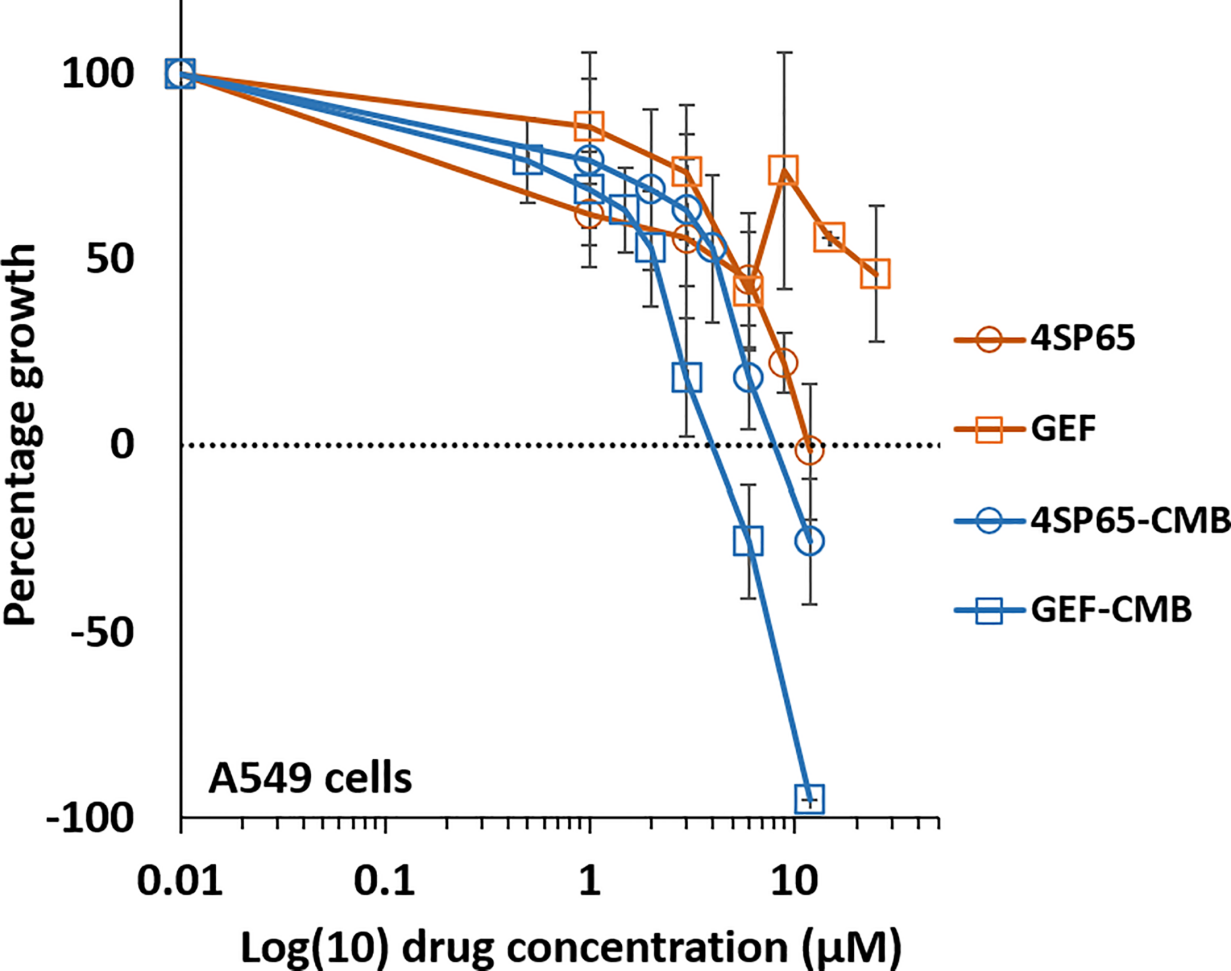
Dose-response curves for growth of human cancer cell lines exposed to 4SP65, gefitinib (GEF), or both drugs. The graph shows percentage of cell growth (cytostatic growth inhibitory effects above dotted lines, total growth inhibitory effects at dotted horizontal lines, and lethal effects below dotted lines) after 48-h treatments, with the dotted line representing starting cell numbers. In the binary drug pair, the concentrations of the individual drugs in the combination are plotted as 4SP65-CMB and GEF-CMB. Data points represent average values across 5 experiments for single or combined drug treatments of A549 cells; error bars represent SEs for the means of related experiments, with a minimum of 4 biological replicates/treatment level included in each experiment (see **Table 7**).

A limited set of experiments was conducted to determine if 4SP65 enhanced the activity of and overcame resistance to the first-generation TKI gefitinib in A549 cells, which are *TP53* wild-type, *KRAS* mutant, and overexpress wild-type EGFR and are considered a representative cell line for innate EGFR-TKI resistance (84). For these experiments, a human cancer cell line was considered resistant to gefitinib when the amount of drug required to achieve cytolytic effects in cell culture was >4.5 µM. This upper limit was based upon reports that, in cancer patients receiving 250 mg gefitinib daily, C_max_ plasma concentrations were 0.5-1 µM or more (85) but trough levels ranged from 0.257−4.50 µM and 0.282−6.55 µM after 3 and 8 days of treatment, respectively, due to the long elimination half-life of 48 h for gefitinib (86). For each cancer cell line, **Figure 6** shows the dose-response curves for cell growth after 48-h treatments with each drug alone or 4SP65-gefitinib combined, while **Table 7** lists the corresponding dose-response metrics, the fold-increase in efficacy for each drug in the combination (with *P*-values indicated where increases are significant), and the synergy scores for the combination.

**Table 7:** Dose response metrics for 4SP65 alone, gefitinib alone, and 4SP65-gefitinib combined (CMB) in drug-exposed human A549 NSCLC cells^a^.

Cell line	4SP65	4SP65-CMB	GEF	GEF-CMB	Fold increase in efficacy	
					4SP65 ÷ 4SP65- CMB	GEF ÷ GEF- CMB
A549 (n = 5 experiments; drug ratio was 67% 4SP65 [µM]:33% GEF [µM])						
IC50	7.4 ± 0.7	5.3 ± 0.8	23.3 ± 5.7	2.6 ± 0.5	1.4**	9.0***
GI50	6.0 ± 0.6	4.1 ± 0.7	17.5 ± 4.4	2.0 ± 0.5	1.5**	8.8**
TGI	11.3 ± 1.5	7.8 ± 0.9	ND (>25)	3.9 ± 0.8	1.5**	>6.4 (*GoE*)
LC50	11.4 ± 2.7	10.9 ± 0.5	ND (>25)	5.4 ± 1.1	1.1 (NS)	>4.6 (*GoE*)
LC99	12.3 ± 3.8	12.5 ± 0.8	ND (>25)	6.2 ± 1.4	1.0 (NS)	>4.0 (*GoE*)
Average HSA (and ZIP) synergy scores = 14.7 (12.0); Most synergistic area scores = 20.5 (16.2)^b^					

a Cell lines were plated in 96-well microtiter dishes, incubated for ~24 h prior to addition of experimental drugs, and then treated over a dose range for 48 h before calculating values for inhibitory concentration 50% (IC50), growth inhibitory concentration 50% (GI50), total growth inhibitory concentration (TGI), lethal concentration 50% (LC50) and 99% (LC99) as set forth by the NCI (56, 83). All units are µM, with means and SEMs. CMB, drug in combination (4SP65-CMB and GEF-CMB refer to the amounts of each drug in combination at each dose-response metric); ND, not definable, with following number in parentheses indicating the highest concentration of drug tested in µM for a given cancer cell line; GoE, gain of effect; NS, non-significant.

b Synergy score data obtained via SynergyFinder (59); scores < -10 likely antagonistic, -10 to +10 likely additive, and >10 likely synergistic on a scale of 60.

** *P* ≤0.01, *** *P* ≤0.001

**Figure 7:**
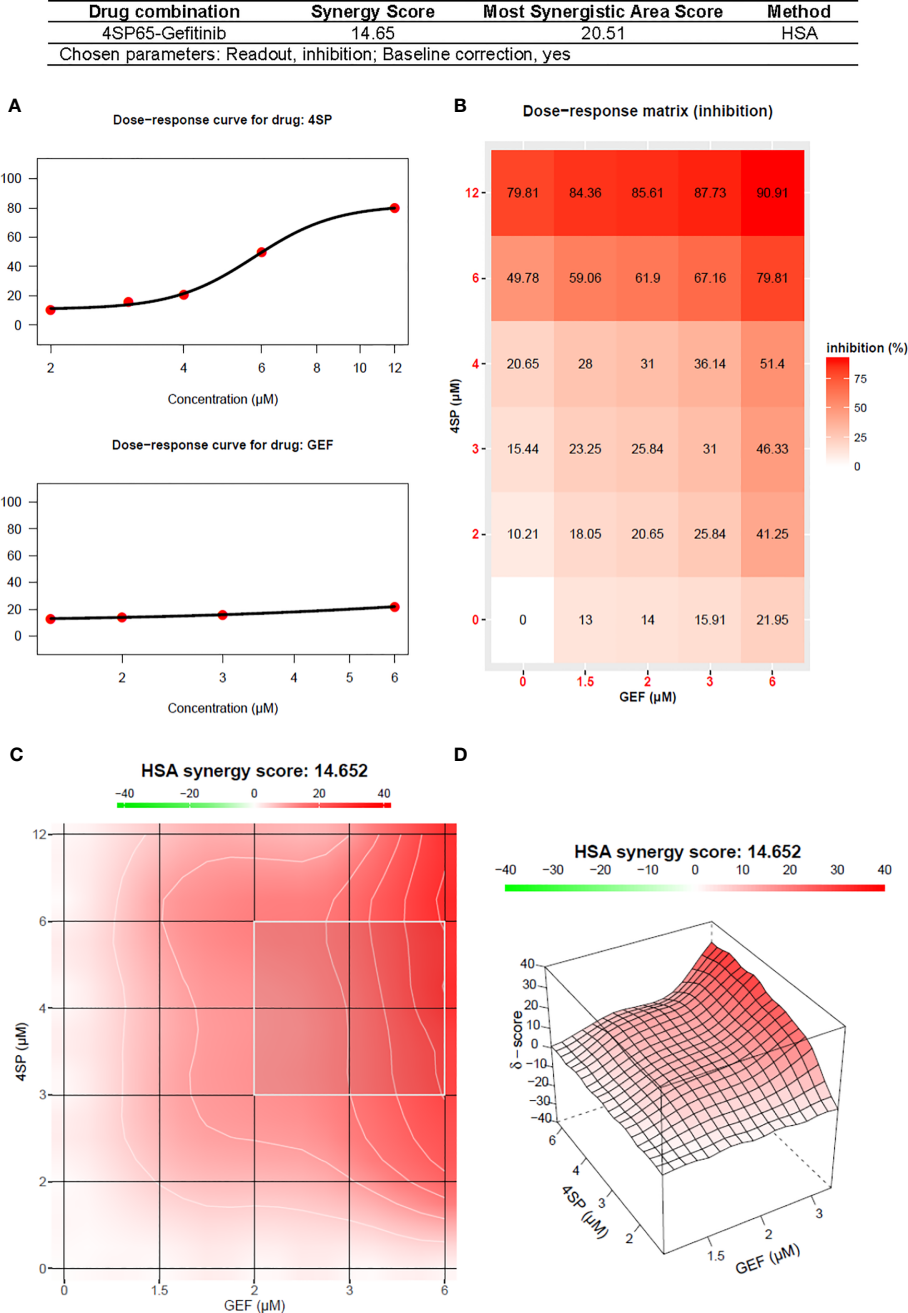
Synergy report for A549 NSCLC cells treated with gefitinib (GEF) alone, 4SP65 alone, or both drugs for 48 h. The report was generated using SynergyFinder 2.0 (https://synergyfinder.fimm.fi) (59). Panels include **(A)** the dose-response curves for 4SP65 alone (upper graph) and cisplatin alone (lower graph), **(B)** the dose-response matrix for growth inhibition by each drug alone and in combination, and **(C)** a two-dimension heat map and **(D)** a three-dimension volcano plot showing the distribution of areas of differing degrees of synergism.

Treatment of A549 cells for 48 h with 4SP65-gefitinib resulted in gefitinib having a significant therapeutic effect at the GI50 and LC50 levels, respectively, when cells were exposed at clinically relevant levels ranging from 2.0 to 5.4 µM gefitinib in the drug combination (**Table 7** and **Figure 6**). By comparison, gefitinib alone exhibited a GI50 of 17.5 ± 4.4 µM in A549 cells, which was nearly four-fold greater than the highest trough concentrations of the drug observed in cancer patients. However, total growth inhibition or cytolytic effects were not induced in A549 cells at a treatment level of 25 µM gefitinib alone. In the combined treatment of A549 cells, the amplification in the effects of gefitinib in combination with 4SP65 was significant for all dose-response metrics, as shown by ~9- and ~4-fold increases in efficacy of gefitinib at the GI50 and LC99 levels, respectively. Co-exposure of A549 cells to 4SP65-gefitinib also yielded significant increases in the growth inhibitory effects of 4SP65 but not in the cytolytic effects of 4SP65 in the combination. The nature of the combined cytostatic/cytocidal effects of 4SP65 plus gefitinib was surveyed further via synergy reports where the upper limits for gefitinib were set at 6 µM to focus on responses at *in vitro* treatment levels corresponding to reported peak plasma trough levels of free drug at 4.50 µM and 6.55 µM after 3 and 8 days of treatment in patients with NSCLC (86). The synergy report for A549 cells shows that despite a nearly flat dose-response curve for gefitinib alone, co-treatment with 4SP65-gefitinib triggered both cytostatic and cytolytic effects at biologically-relevant concentrations of both drugs, achieving ~91% reduction in starting cell numbers at 6 µM gefitinib plus 12 µM 4SP65 (**Figure 7**). The dose-response matrix in the synergy report shows that 48-h co-treatment with 4SP65-gefitinib resulted in ~62% to 80% reduction in starting cell numbers as the concentration of gefitinib was increased from 2 to 6 µM in combination with 6 µM 4SP65. The heat map and volcano plot further demonstrate that the average HSA synergy score of 14.7 was driven by the most synergistic area score of 20.5 occurring between 2 and 6 µM gefitinib and 3 and 6 µM 4SP65.

## Discussion

The original goal of this research was to design a new prodrug for the intracellular delivery of WR1065 or other Walter Reed aminothiols (e.g., WR255591 of phosphonol) (1, 87) to both normal and diseased cells. During this process, it became clear that the initial BRG prodrugs, 4SP65 and 1LP65, showed surprising therapeutic effectiveness against a range of human cancer cell lines that were *TP53* wild-type, mutant, or null. 4SP65 and 1LP65 had significantly greater cytostatic activity than amifostine and showed dose-dependent cytolysis in all human cancer cell lines tested, while amifostine failed to induce cytolytic effects. 4SP65 also induced cell killing effects in human cancer cell lines where cisplatin or paclitaxel did not achieve such effects at safe drug levels, and 4SP65 did so without cytotoxicity to normal cells. Both synergistic and drug resistance reversal effects were induced by 4SP65 in combination with two different types of anticancer agents, the cytotoxic chemotherapeutic cisplatin and the first-generation EGFR-TKI gefitinib.

The differences in *in vitro* cell fate decision outcomes induced by amifostine versus the BRG prodrugs probably reflect a multiplicity of factors beyond differences in drug uptake, metabolic activation, and number of active ingredients (1). Amifostine is converted to its active form by an alkaline phosphatase-mediated hydrolysis reaction, resulting in cellular uptake of WR1065 as a protonated thiol. In contrast, after being taken into cells, the BRG disulfide prodrugs can be converted into active ingredients by oxidation/reduction and/or thiol-disulfide exchange reactions, resulting in the potential for BRG components to participate in a series of complex reactions (88). These differences are postulated to account for some of the divergence in observed anticancer efficacy of these three drugs. Another source of potential differences comes from SwissADME ligand-binding molecular modeling (89) that indicates a limited predicted overlap between potential targets for WR1065 and m-PEG_6_-thiol (**Supplementary Table 1** and **Supplementary Figure 8**), with the thiol-PEG polymer predicted to bind to a range of kinases, caspases, metalloproteinases, and other enzymes involved in oncogenesis. These predictions raise the potential for WR1065 and the thiol-PEG polymers to work synergistically, and suggest that the thiol-PEG polymers contribute to the striking differences in the anticancer efficacy of 4SP65, 1LP65, and amifostine in the same cancer cell lines (**Figure 3**).

Studies showing differences in BRG prodrug effectiveness when the growth medium was, or was not, supplemented with plasma membrane transport modulators support the conclusion that BRG prodrugs are taken up by one or more plasma membrane transport systems and drug efficacy is affected by the degree of drug uptake. This phenomenon has important implications for achieving anticancer efficacy with the BRG prodrugs *in vivo* because there are safe methods for modulating plasma membrane transport activity in animal models and cancer patients (90–92). This approach can make it possible to ensure adequate BRG prodrug uptake in tumor cells and induction of rapid tumor shrinkage.

The initial studies reported here were focused upon demonstration of the range of BRG prodrug anticancer effectiveness, while deferring mode of action investigations for later work. However, WR1065 is one active ingredient of the BRG prodrugs and thus a succinct review of reported activities of WR1065 can provide insight into potential mechanistic activities of the BRG prodrugs. Studies of WR1065-mediated effects upon p53 gene expression and protein activity show that it induces increased expression of p53 target genes and affects p53-regulated pathway activity (31, 35). In normal and cancer cells, WR1065 is reported to increase and maintain the level of nuclear p53 protein for up to 60-70 h (31, 32, 93); some evidence supports the postulate that this effect is dependent upon competent DNA repair (93). By activating and stabilizing the p53 protein, WR1065 achieves the full impact of p53-mediated functionality; without stabilization p53 is degraded rapidly (94). These WR1065-mediated effects are attributed to binding (under non-reducing conditions) to p53 protein with resultant induction of conformational changes and enhanced DNA binding (31, 32, 35). WR1065 also can induce p53 protein up-regulation through indirect mechanisms that are postulated to result from induction of cellular stress responses (32).

In *in vitro* models, WR1065 has been reported to restore wild-type conformation and activity in an array of temperature-dependent p53 mutants (34–36, 95) that are considered damaging to normal p53 function (96). The majority of p53 mutations are missense, with ~93% affecting the DNA-binding domain including each of the 50 most common missense mutations in human cancers (95, 96). Among these top 50 p53 mutations, 11 temperature-dependent mutants originating from several different human cancers have been analyzed for the effects of WR1065 on the transactivation capability and conformation of p53. WR1065 was found to reactivate p53 to some degree in 8/11 (~73%) of these mutants (34–36, 95). Temperature-dependent p53 mutants are remarkable because they feature only limited and reversible perturbations of the thermodynamic equilibrium of the DNA-binding domain that can be functionally rescued just by a temperature shift toward the permissive value (95). These data suggest that a majority of p53 mutations with key roles in cancer are amenable to functional rescue by small molecules like WR1065.

WR1065 also has effects that go beyond p53, including interacting with multiple transcription factors and altering the gene expression profiles of components involved in cell cycle arrest and programmed cell death (PCD). In normal cells, WR1065 induces a p53-dependent G1 arrest (30, 33, 35, 93, 97). This arrest is considered to have a major role in the cytoprotective effects of WR1065 in normal cells (93), and helps explain the differential effects of WR1065 in p53-competent versus p53-incompetent cells (31, 93). In cancer cells, arrest in both G1 and G2 has been reported. Lee et al. (93) detected arrest at G1 for p53-proficient HCT116 human colon cancer cells and at G2 for their p53-incompetent counterparts. In contrast, WR1065 was reported to induce G1 arrest in a human p53-incompetent endometrial tumor cell line (10), and G2 arrest in a different p53-incompetent endometrial tumor cell line (15). Both Dai et al. (10) and Luo et al. (15) attributed tumor cell death to the WR1065-induced arrest at these checkpoints. Thus, the checkpoints at which WR1065 induces arrest do not explain completely the observed outcomes.

Reported effects for WR1065 also provide insight into possible PCD pathways and cell death induction mechanisms by BRG prodrugs. The p53 protein has a major role in both intrinsic and extrinsic apoptosis (98) and thus these pathways constitute one process by which BRG prodrugs can induce cell death. WR1065 induced p53-dependent PCD processes in human endometrial cancer cells (15), A549 NSCLC cells (99), p53-proficient human HCT116 colon cancer cells (93) and human melanoma cells (100). WR1065 also induced p53-independent PCD processes, as reported for a myelodysplastic syndrome cell line (101), HL60 cells (102), K652 leukemia cells (14), Dami leukemia cells (103), and HCT116 human colon cancer cells lacking p53 activity (93). WR1065 also affected transcription factors beyond p53, including NF-κB, and AP-1 (31, 32, 104), and was postulated to affect other transcription factors with redox-sensitive regulatory cysteines (32). Grdina et al. (105) showed that WR1065 activated the NF-κB p50-p65 heterodimer, but not complexes with p62 or c-Rel, in SV40-immortalized human endothelial cells and glioma cell lines U87 (p53 competent) and U251 (p53 mutant). WR1065 modulated JNK phosphorylation and binding (105), and JNK has roles in multiple PCD pathways including intrinsic and extrinsic apoptosis, necroptosis, autophagy, pyroptosis, and possibly ferroptosis (98, 106).

The results reported here place BRG prodrugs among a few anticancer agents that function to reactivate mutant p53 protein (4), such as APR-246, which interacts with key cysteine thiols via the active metabolite methylene quinuclidinone (75). However, WR1065 differs from methylene quinuclidinone in several respects. Methylene quinuclidinone, as well as other p53 reactivators for which information is available, are Michael acceptors that react with nucleophiles (107). In contrast, WR1065 and the thiolated-PEG polymers generally function as nucleophiles (88), and thus their binding sites on key proteins, and conditions affecting this binding, can differ from those of methylene quinuclidinone. To gain extended p53 activity (108), APR-246 and PRIMA-1 are combined with agents that block murine double minute 2 (MDM2) and prevent degradation of p53, while WR1065 both activates p53 protein and inhibits its degradation via a mechanism distinct from MDM2 inhibitors (17, 32).

Although available reports of WR1065 modulatory effects upon gene and protein expression profiles are few, they reveal an interesting pattern that suggests a mechanistic hypothesis for BRG-induced activities. These activities include (i) safety in normal cells, (ii) broad-spectrum anticancer activity, (iii) synergistic effects in combination with other drugs, and (iv) drug resistance reversal effects. In brief, gene expression studies in normal cells report that WR1065 alone enhances pro-survival stress-response related genes and proteins while suppressing pro-cell death components/pathways. This profile is enhanced further if WR1065-pretreated cells are exposed to a stress-inducing agent such as ionizing radiation within the appropriate time frame. These effects provide an explanation for the safety of BRG prodrugs in normal cells and for the cytoprotective effects of WR1065 in cells exposed to stress conditions. Conversely, an opposite pattern is reported in cancer cells where WR1065 alone enhances expression of genes encoding pro-cell death components and suppresses pro-survival gene expression. This altered gene expression profile is enhanced further if cancer cells are exposed to a second stress-inducing agent, resulting in reduction of barriers to PCD pathways and induction of cell death. Some of the commonly reported PCD components modulated by WR1065 include p53, caspases, BCL2 family members, and pathways including MAPK and PI3K/AKT. Although these paradoxical pro-survival/pro-death effects are unexpected, they are outcomes of cellular stress response systems, which are regulated to a large degree by the status of oxygen/sulfur redoxomes (109, 110). Blockades to PCD pathways are postulated to confer broad-spectrum intrinsic and acquired drug resistance capacity to a wide range of neoplasms (47, 111, 112). Thus, the ability of BRG prodrugs to overcome these barriers in tumor cells provides an explanation for their synergism and drug resistance reversal effects when combined with other anticancer agents.

Evidence supporting this hypothesis in normal cells comes from several studies. Khodarev et al. (113) examined changes in expression of a family of genes with roles in apoptosis and stress-responses in microvascular endothelial cells exposed *in vitro* to WR1065 alone, ionizing radiation alone, or the combination. In the category of apoptosis and stress response genes, WR1065 alone up-regulated a few pro-death/stress response genes, down-regulated several, and had no effect upon a third group. However, when cells were exposed to WR1065 combined with ionizing radiation, the expression of most pro-PCD genes was suppressed. Similar modulation of pro-survival pathways and elements was observed in human pulmonary artery endothelial cells exposed to WR1065 and bacterial lipopolysaccharides, H_2_O_2_, or IL-6 (114). Shen et al. (31) also found that WR1065 protected immortalized cells with wild-type p53 from paclitaxel-induced cell death, but enhanced cell death in immortalized cells with mutant p53 and in cancer cells with either wild-type or mutant p53. Similar modulation of PCD components and pathways has been reported for *in vivo* studies in animal models exposed to WR1065 via amifostine. In mice exposed to amifostine with and without concomitant radiation exposure, Segreto et al. (115) showed that WR1065 reduced numbers of apoptotic cells in bone marrow, and increased p53 protein expression in radiation-naïve and radiation-exposed granulocytes. WR1065 prevented or reduced radiation-induced caspase-3 cleavage, activation of PUMA, and phosphorylation of p38. Yoon et al. (116) found that WR1065 reduced p53 and Bax expression in immature ovary of mice exposed *in vivo* to WR1065 and ionizing radiation.

Studies in cancer cells show that, compared to effects in normal cells, WR1065 has a different impact upon pro-apoptotic and anti-apoptotic components. Bianchini et al. (14) studied the combinatorial effects of WR1065 alone and in combination with imatinib in K562 leukemia cells, which are p53 mutant and imatinib resistant. In cells exposed to WR1065 alone, a subset of pro-apoptotic genes was up-regulated and a subset of anti-apoptotic genes was down-regulated. When WR1065 was combined with imatinib, these effects were enhanced. Rosalski et al. (11) reported similar trends for HL60 cells (p53-null) exposed to WR1065 alone or in combination with doxorubicin. In these studies, significant changes in caspase-3, p65, and bax were reported. Rho et al. (117) investigated the potential role of p53 in the growth inhibitory and apoptotic effects induced by gefitinib in A549 cells, which are *TP53* wild-type with no oncogenic *EGFR* mutations. Treatment of A549 cells with gefitinib alone induced translocation of wild-type p53 from the cytosol with accumulation in the nucleus. This event resulted in p53- and caspase-dependent enhancement of cancer cell growth inhibition and apoptosis through up-regulation of Fas and caspase, PCD components also reported to be modulated by WR1065 (115, 118). These effects can explain the synergism between 4SP65 and gefitinib in A549 cells. Comparable gene expression studies have not been conducted in human patients, however, the probability that similar changes underly the anti-cancer effects of amifostine in human patient trials is supported by multiple reports of improved responses to therapy for patients receiving amifostine in combination with a second anticancer agent (17–21). Diverse mechanisms are involved in multi-drug resistance, and it is postulated that drugs with multi-modal modes of action are needed to address this problem (119). The above described activities of BRG prodrugs in cancer cells and the diversity of WR1065 effects upon cellular stress response system components support the conclusion that BRG prodrugs can function as broad-spectrum drug resistance reversal agents.

Multiple authors studying WR1065-related anticancer outcomes have postulated that these effects reflect WR1065 modulation of the cellular redox balance (10, 15), a hypothesis consistent with the major role of the oxygen and sulfur redoxomes in cellular stress response resolution outcomes (109, 110). WR1065 can act as a hydrogen donor and by increasing the levels of reduced glutathione (GSH) to maintain or enhance the approximate 100:1 GSH:GSSG ratio (120, 121) and the reductive capacity of the cell cytosol. The antioxidant and free radical scavenging activities of WR1065 have been reported widely (22). However, both WR1065 and the thiol-PEG polymers are reactive sulfur species. Due to the diversity of reactions to which cysteines are vulnerable (88), the altered cellular milieu in neoplasia can result in a broad, diverse array of potential binding sites for BRG thiols, sites that can differ significantly from those available in normal cells. Multiple binding sites then offer opportunities for BRG prodrug components to alter protein conformation, activity, and intracellular signaling. These considerations support the postulate that there are additional, significant modes of action for the BRGs that await further investigation.

In summary, the BRG prodrug family offers a novel strategy for intracellular delivery of WR1065 or other bioactive aminothiols for effective and safe uses in new clinical areas, particularly cancer treatment. The *in vitro* findings that 4SP65 induces near-to-complete cell death in all cancer cell lines tested to date augur well for successful translation of the BRG prodrugs to demonstration of *in vivo* efficacy (54). The findings presented here and in various reports of amifostine/WR1065, which show cytoprotective effects in normal cells and concomitant anticancer effects in neoplastic cells, further support this conclusion. Additional studies are needed to unravel the mechanisms underlying the differential effects of BRG prodrugs as anticancer agents that do not harm normal cells. Future advancements of BRG prodrugs have the potential to improve the lives of cancer patients by providing effective therapeutics that protect normal cells and address the problem of anticancer drug resistance.

## Data availability statement

The raw data supporting the conclusions of this article will be made available by the authors, without undue reservation.

## Author contributions

Conception and design, DW, MP, VW. Development of methodology and protocols, DW, TL, SR, TM, BC, VW. Generation of data (performed experiments, provided facilities and human cell lines, characterized and verified authenticity of cell lines, etc.), DW, MP, TM, BC, VW. Analysis and interpretation of data (e.g., data modeling and computational analysis, statistical analysis): DW, BC, VW. Administrative, technical, or material support (i.e., study supervision, organizing data, constructing databases), DW, VW. Preparation of figures and tables and writing of the manuscript, DW, TL, SR, VW. All authors contributed to the article and approved the submitted version.

## In memoriam

This article is dedicated in memory of Patricia (“Pat”) B. Upton, an outstanding senior research technologist at the University of North Carolina Chapel Hill, who was a great resource to graduate students, post-doctoral fellows, visiting scientists, and professors/researchers in her group. After early retirement, her battle against recurrent lung cancer and participation in clinical trials served as an inspiration for ongoing research concerning therapeutic options to overcome drug resistance. Her friendship and talents to support and nurture nascent researchers will be deeply missed.
