# Supplementary material for: WR1065 conjugated to thiol-PEG polymers as novel anticancer prodrugs: broad spectrum efficacy, synergism, and drug resistance reversal
Source: Front Oncol. 2023 Jul 28;13:1212604. doi: 10.3389/fonc.2023.1212604 (PMC10419174; doi:10.3389/fonc.2023.1212604)
Supplement: Supplementary file 1 [file DataSheet_1.pdf]

Supplementary Material

WR1065 Conjugated to Thiol-PEG Polymers as Novel Anticancer Prodrugs: Broad Spectrum Efficacy, Synergism, and Drug Resistance Reversal

Dale M. Walker, Tsvetelina I. Lazarova, Steven W. Riesinger, Miriam C. Poirier, Terri Messier, Brian Cunniff, and Vernon E. Walker*

*** Correspondence:** vwalker@uvm.edu

1. Supplementary Data - Synthesis of 4SP65 and 1LP65; Solubility, storage, and stability of 4SP65 and 1LP65; Figures S1-S5; and Table S1.

Synthesis of 4SP65 and 1LP65

The scheme for the synthesis of 4-'star' PEG-S-S-WR1065 (4SP65) is shown in **Figure S1**, with the steps described in brief below. Further details of the synthesis of 4SP65 are described in patent # WO 2017/087668 or PCT/US/2016/062526, entitled *Methods for improved protection and delivery of aminothiols and analogs thereof*. **Step 1:** WR1065 as a dihydrochloride salt (1.21 mmol of *substrate 1*) was dissolved in anhydrous dichloromethane (5 mL). Triethyl amine (6 eq) and boc anhydride (2.1 eq) were added and the reaction was stirred at ambient temperature overnight under a positive nitrogen atmosphere. The next day, the reaction was diluted with dichloromethane and washed with brine. The organic layer was dried over magnesium sulfate and concentrated in vacuo to give *compound 2* as a clear oil (85% yield). **Step 2:** *Compound 2* (1.03 mmol) was dissolved in 1/1 water/methanol (10 ml) and disulfide (2 eq) (*compound 3*) was added. The reaction was stirred at ambient temperature under nitrogen overnight. The next day, the reaction was concentrated in vacuo, diluted with dichloromethane, washed with brine, and dried over magnesium sulfate. The resulting *compound 4* was purified by column chromatography with silica gel and hexane/ethyl acetate gradient (46% yield). **Step 3:** A solution of 4arm-PEG-SH or star polymer (0.75 g of *compound 5*, average molecular weight 10,000) in PBS (8 ml, pH 7.4) was added to a solution of *compound 4* (0.45 mmol) in ethanol (2 ml). The reaction was stirred for 4 h at ambient temperature and then lyophilized overnight. The crude was dissolved in water (4 ml) and DMSO (2 ml) and was dialyzed against water for 48 h with four water changes. Afterwards, the solution was lyophilized and *conjugate 6* was isolated. **Step 4:** *Conjugate 6* was treated with 1/1 trifluoroacetic acid/dichloromethane (5 ml) for 30 min. The solvent was removed in vacuo and the residue was dried on a vacuum pump overnight. The next day, the residue was washed with ethyl ether (twice) and further dried on a vacuum pump overnight to yield *conjugate 7* or 4-'star' PEG-S-S-WR1065 (4SP65) as a viscous aggregate polymer. The spectrum from MALDI of *conjugate 7* showed a peak corresponding to 4SP65 at an average MW of 10,531.95 Da (**Figure S2**). The boc deprotected aminothiol conjugated to 4arm-PEG-SH (10,000 Da) was 146 Da so that if all four arms were reacted the result would be a polymer of 10,584 Da, indicating that the 4SP65 molecules used in reported experiments had nearly four units of WR1065 incorporated on average.

As indicated in the main text, the same synthetic scheme was used in the synthesis of m-PEG$_6$-S-S-WR1065 (1LP65) except that m-PEG$_6$-thiol was used a PEG-SH scaffold for *compound 5* in **Step 3** to yield 1LP65 as *conjugate 7* in **Step 4** (**Figures S3 and S4**).

Solubility, storage, and stability of 4SP65 and 1LP65

The solubility of 4SP65 was tested in de-ionized water, PBS, DMSO, and RPMI or DMEM base medium with and without FBS. While the drug was partially soluble in all media evaluated except DMSO, a portion of the drug absorbed liquid to form a gel that was consistent across all aliquots of drug

prepared for use and that was not altered by the solvent used, heating up to 37°C for up to 3 days, and shaking or rocking for up to 3 days at RT or 4°C. For experiment, an aliquot of drug was first mixed in medium, the solvent was removed and saved, and gel pieces were recovered, dried on a vacuum pump, and weighed to determine the mass of drug that did not dissolve. The difference in the starting weight of drug placed into solvent minus the weight of the residue after drying on a vacuum pump was considered to represent the amount of drug that went into solution. The amount of drug that was dissolved at a given time ranged from 40 to 61%, averaging 50.7 ± 8.1% (SD). The weight of the 4SP65 in solution was then used for diluting the drug to concentrations of 200 or 400 µM in supplemented RPMI or DMEM/F12 medium for routine treatment of cells. The dried residue was not used in the in vitro drug efficacy studies. The formation of a gel in the aqueous solvents tested was unexpected since the 4SP65 molecule was found to have nearly four units of WR1065 incorporated on average, leaving few free thiols in polymer arms of the PEG scaffold for cross-linking reactions to occur. However, unintended topical entanglements between polymer chains of multi-arm PEG-thiol molecules without disulfide formation have been reported (Tang and Bradley 2014 24860800). Aliquots of 4SP65 dissolved in FBS enriched medium has remained stable for >6 months while remnants of the original viscous polymer have maintained stability without any apparent loss of activity when stored for 60 months at –20°C.

The boc deprotected aminothiol conjugated to m-PEG6-thiol was dried on a vacuum pump overnight to yield a viscous oil that on analysis gave rise to spectra corresponding to 1LP65 as a di-trifluroacetic acid salt at a MW of 672.87 Da or a MW of 445 (**Figure S4**) once dissolved in solution. As such, 1LP65 is soluble 100% DMSO or aqueous solution. However, the bulk of the product was dissolved at 40 mM in DMSO and stored in aliquots at –80°C for future use once by further dilution in DMSO and then final dilution in medium for treatment of normal human epithelium and human cancer cell lines at working solutions of 4 mM or 400 µM drug. 1LP65 has maintained stability for six months in frozen aliquots but should be stable in DMSO at –80°C for two to three years based upon recommended storage conditions for cystamine dihydrochloride (Selleck Chemical product information), an analogous 225 Da compound with a disulfide bond and amine groups like 1LP65.

2. Supplementary Figures and Tables

2.1 Supplementary Figures presented below in the following order:

Supplementary Figure 1 - Schematic for synthesis of 4-star PEG-S-S-WR1065 (4SP65)
Supplementary Figure 2 - MALDI-TOF spectra of 4SP65
Supplementary Figure 3 - NMR spectra for mPEG$_6$-S-S-WR1065 (1LP65)
Supplementary Figure 4 - ESI+ Mass spectrum for 1LL65
Supplementary Figure 5 - Comparison of relative cell survival measured using alamarBlue versus CyQUANT® dye in H1437 cells treated with 4SP65 or cisplatin
Supplementary Figure 6A-F - Synergy reports for human cancer cell lines treated with 4SP65, cisplatin, or both drugs in combination
Supplementary Figure 7 - Synergy report for A549 NSCLC cells treated with 4SP65, gefitinib, or both drugs in combination
Supplementary Figure 8 - Pie charts showing the relative distribution of molecules targeted by 1LP65, mPEG$_6$-SH (1LP polymer), and WR1065 based upon SwissADME modeling.

2.2 Supplementary Table 1 - Swiss ADME web tool predicted targets listed for mPEG$_6$-S-S-WR1065 (1LP65), mPEG$_6$-SH (1LP polymer), and WR1065

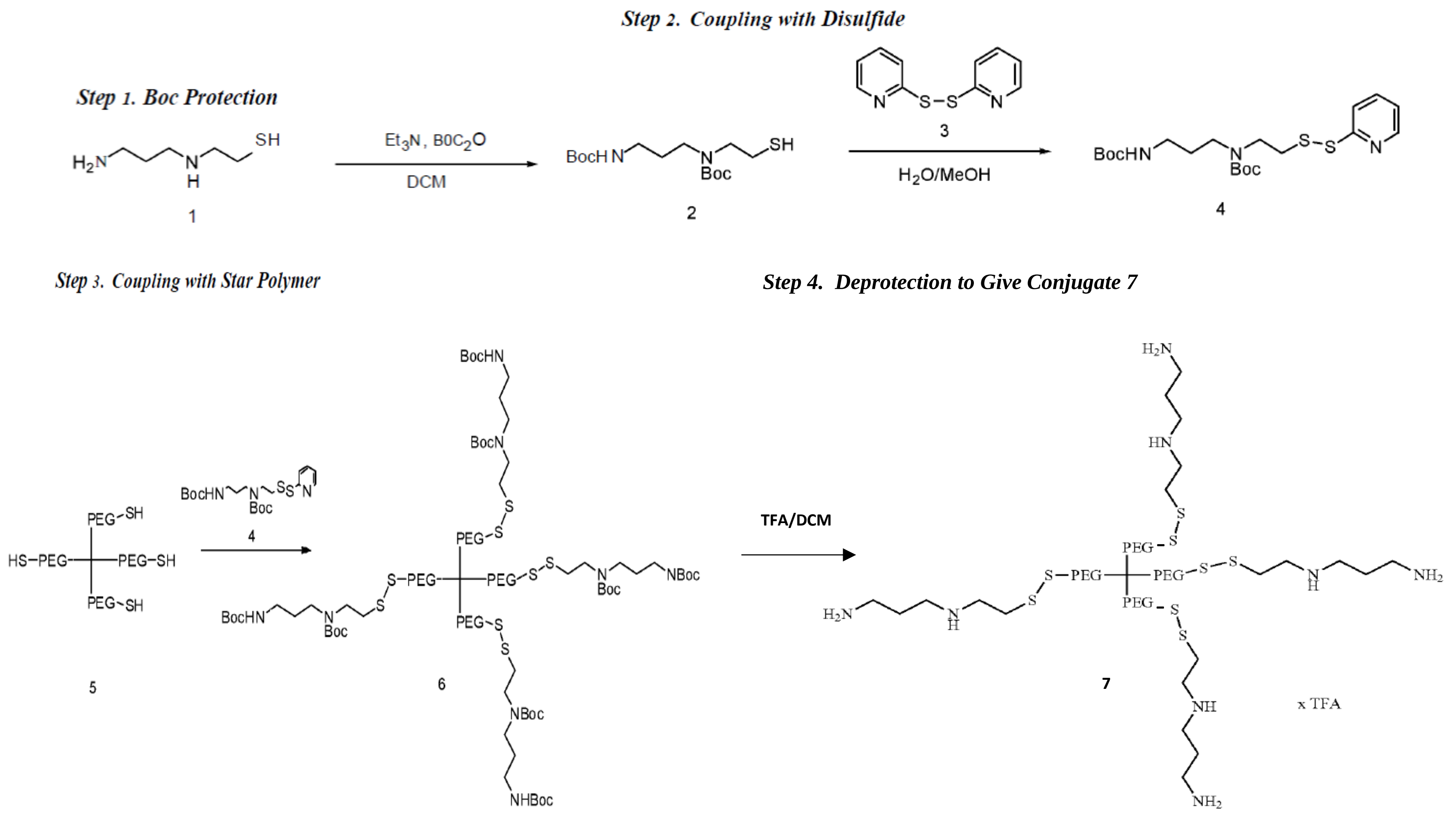

Supplementary Figure 1. Schematic for synthesis of 4-star PEG-S-S-WR1065 (conjugate 7), abbreviated 4SP65. *Step 1*. WR1065, substrate 1, in anhydrous dichloromethane (DCM) was dissolved with triethyl amine (Et_3N) and Boc anhydride (Boc_2O) to generate a substrate 2, providing Boc protection of WR1065 amine groups. *Step 2*. Substrate 2 was dissolved in water/methanol (MeOH) and disulfide (substrate 3) was added for coupling of substrates 2 and 3 to yield compound 4. *Step 3*. A solution of compound 4 in ethanol was added to a solution of 4-arm PEG-SH polymer (average MW 10,000) (compound 5) to generate conjugate 6. *Step 4*. Conjugate 6 was treated with 1/1 trifluoroacetic acid (TFA)/DCM, the solvent was removed in vacuo, and the residue was dried on a vacuum pump overnight to achieve Boc deprotection and obtain conjugate 7.

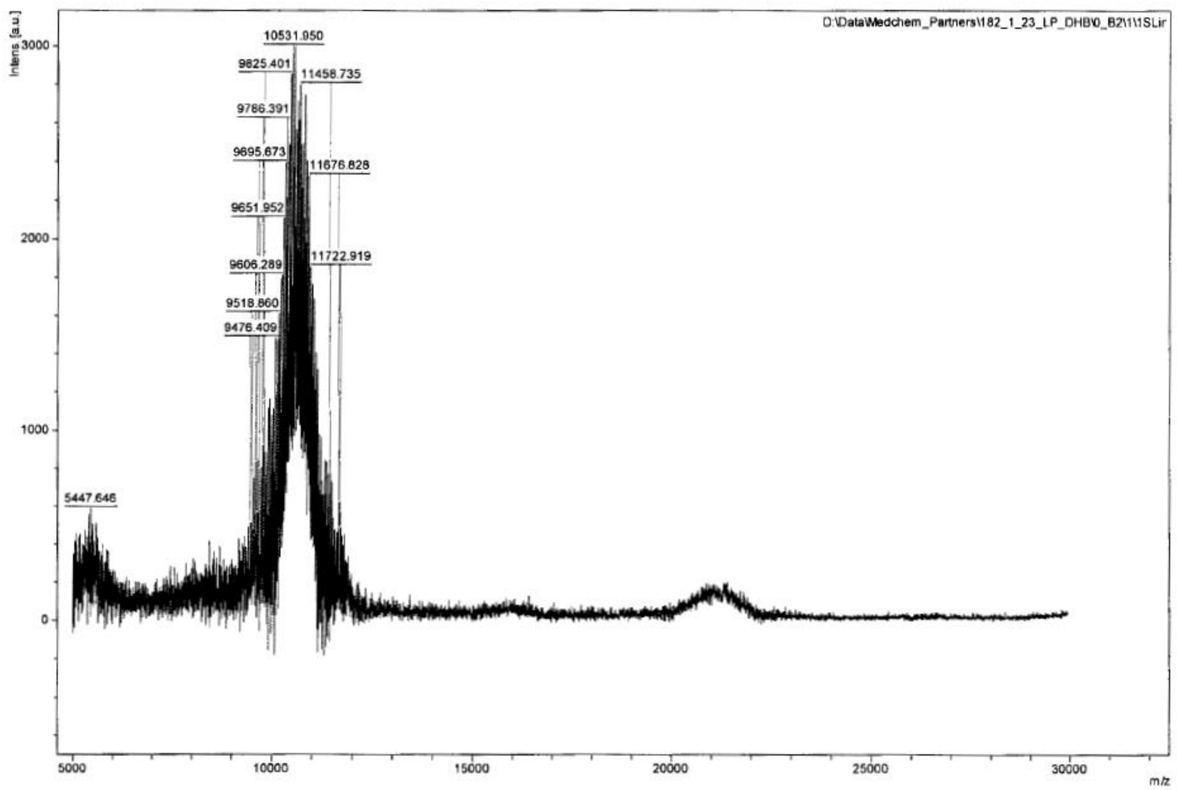

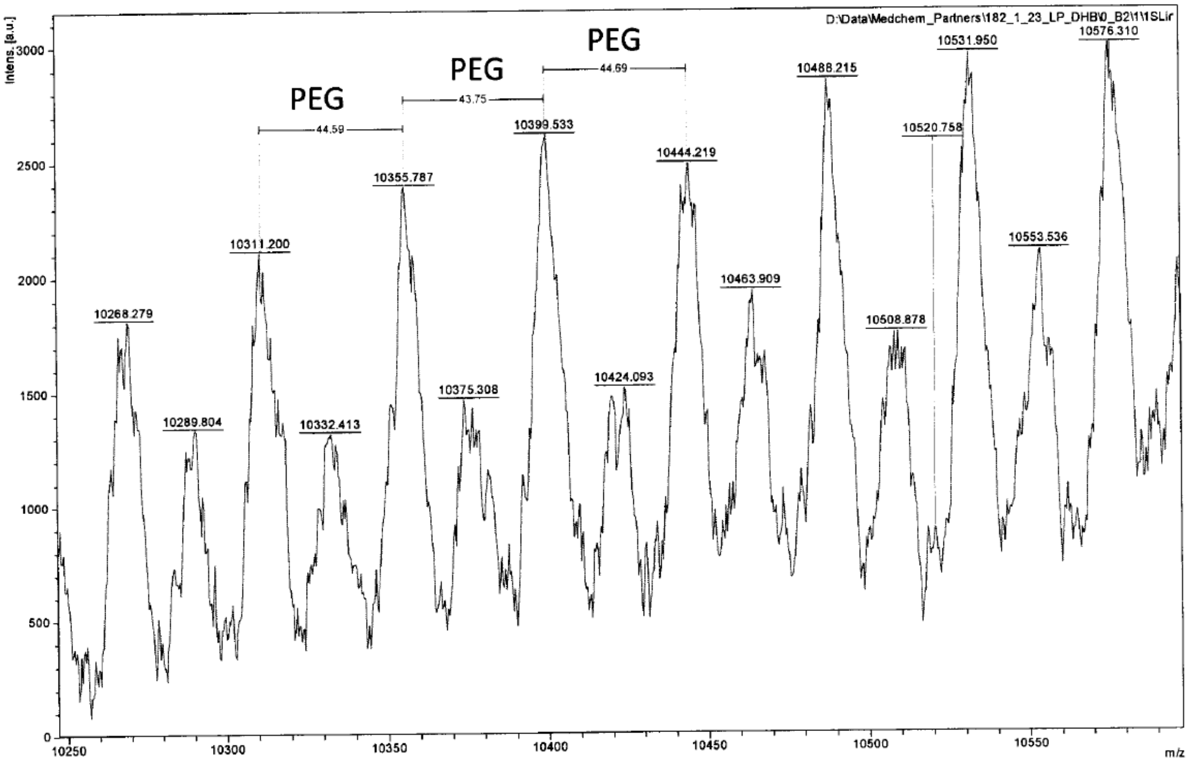

Supplementary Figure 2. *Top*) MALDI-TOF spectra of 4SP65 showing the AMW= 10531.950 which corresponds to the incorporation of four molecules of WR1065 onto the 10,000mw backbone of the 4-arm PEG polymer. *Bottom*) Expansion of the MALD-TOF spectra showing the PEG repeats in the backbone of the polymer.

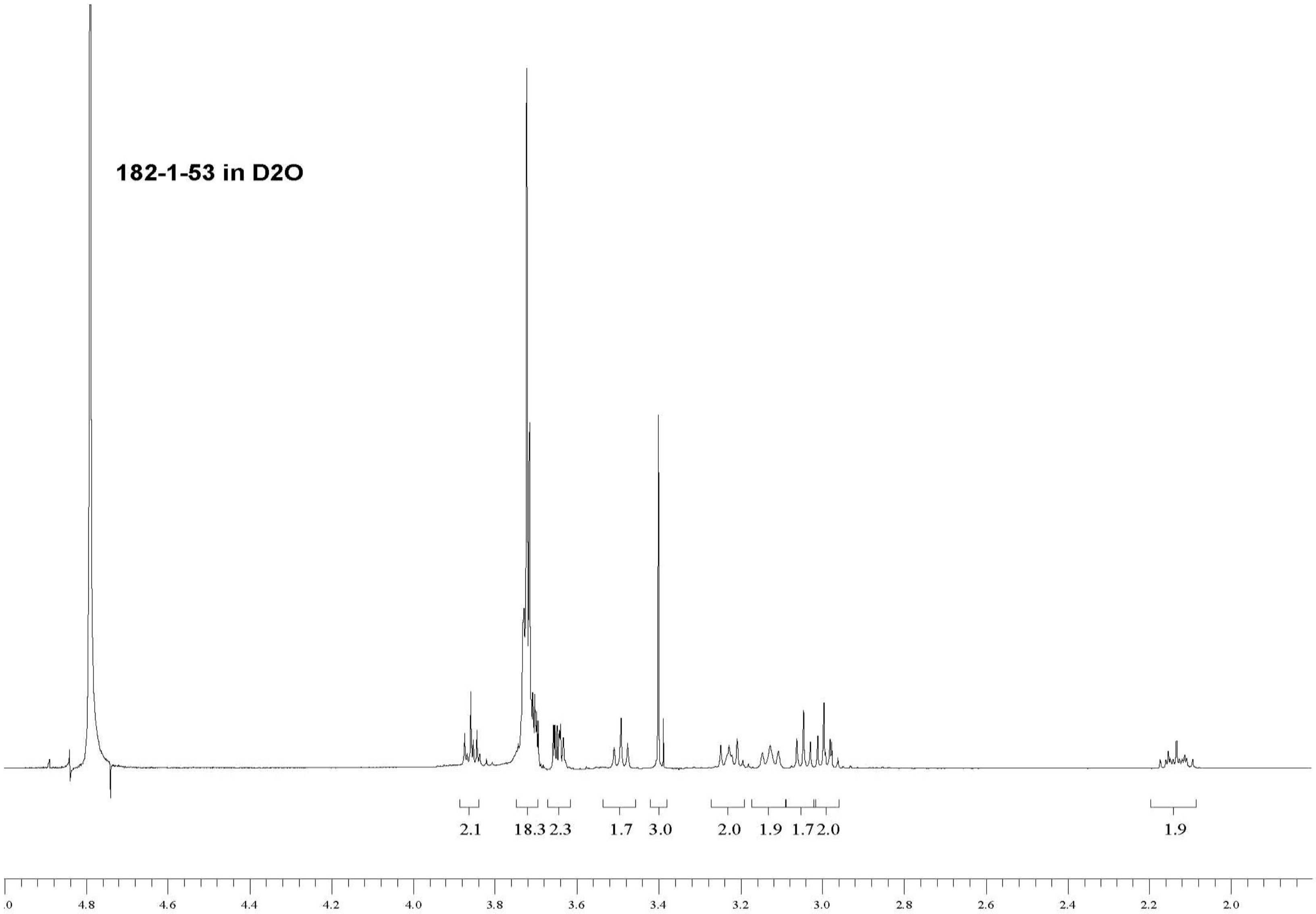

Supplementary Figure 3. 400 MHz1H NMR spectra for 1LP65 in D2O the peaks at 3.88(2H), 3.71(18H), 3.65(2H), 3.4(3H), and 3.06 (2H) are from the mPEG6-SH backbone, the other resonances are from the 2-((3-aminopropyl)amino)ethanethiol moiety.

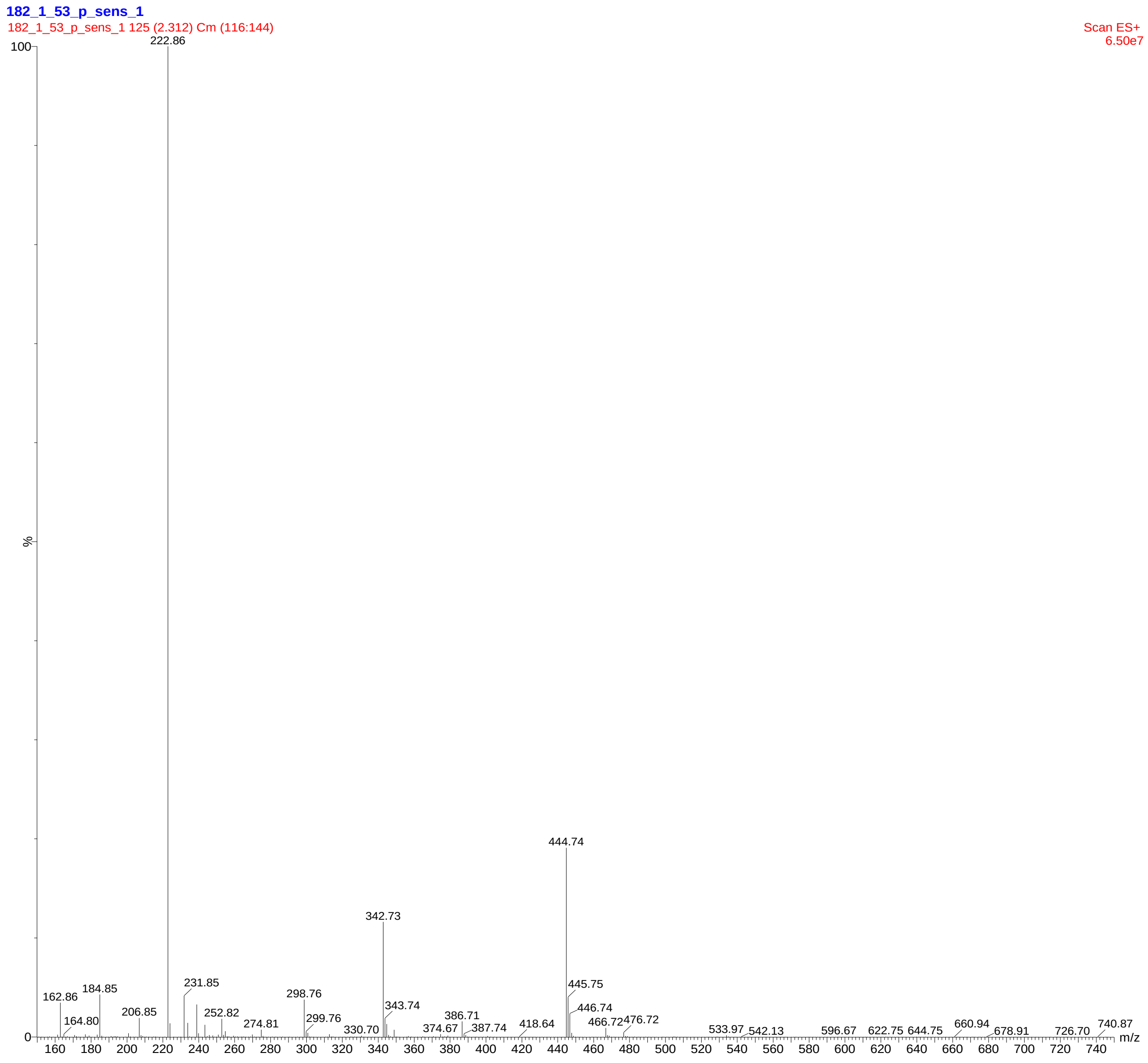

Supplementary Figure 4. ESI+ Mass spectrum for 1LL65 showing the mass of 444.74 (M+H) which corresponds with the calculated mass for this structure.

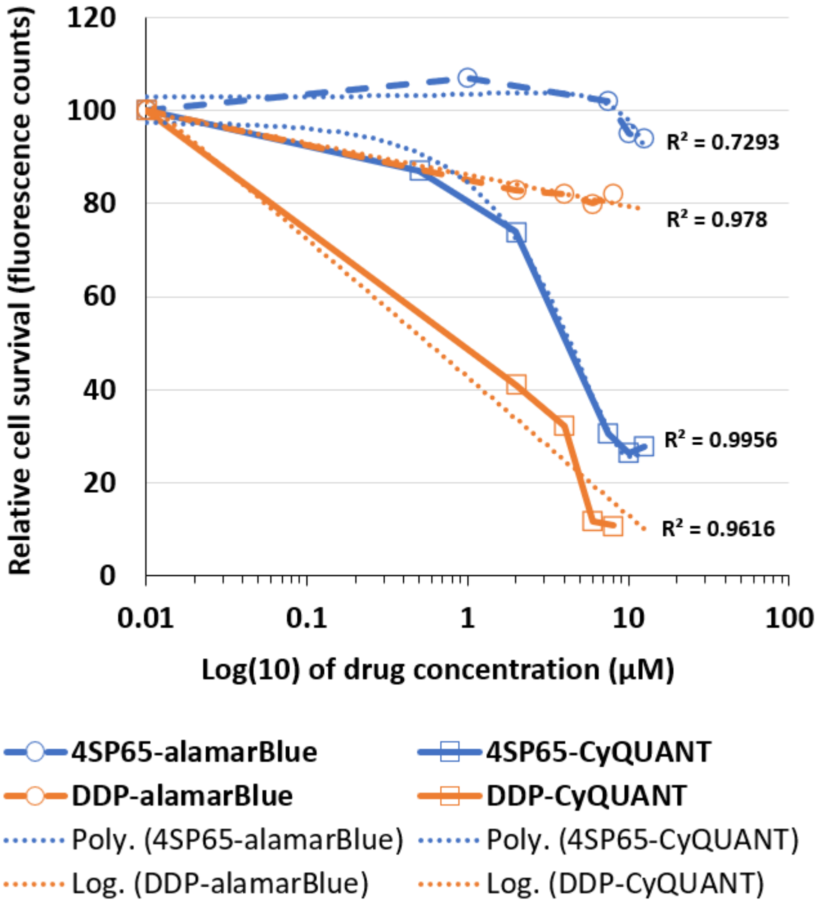

Supplementary Figure 5. Comparison of relative cell survival based upon fluorescence counts for H1437 cells exposed to 4SP65 or cisplatin for 48 h. Cell viability was evaluated using alamarBlue or CyQUANT® dye. Best-fit trendlines fitted to the relative cell survival curves for H1437 cells exposed to 4SP65 (blue) or cisplatin (DDP; orange) using Excel had R_2 values above 0.95 for all curves except H1437 cells exposed to 4SP65 and assessed using alamarBlue. The 4SP64-alamarBlue curve has a best-fit trendline R_2 value of ~0.73; note that other trendlines were fitted to the data but the R_2 values were lower than 0.73. The shape of the trendline for this curve is relatively flat, indicative of near to complete reduction of resuzurin to resorufin until relative cell survival has been reduced to approximately 30%. AlamarBlue is reported to be reduced primarily by NADPH (1), thus, supporting the conclusion that WR1065 maintained NADPH in its reduced form until the number of viable cells had been lowered significantly. Maintenance of the reducing capacity of cells has roles in both cell protective effects and in anticancer effects, with caspases and phosphatases reported to require a reducing environment for activity (2, 3). Note that the dose response curves for H1437 cells exposed to cisplatin (DDP) are linear for both alamarBlue and CyQUANT®, indicating that cisplatin does not have a significant effect upon the reduction capacity of H1437 cells.

1. Rampersad SN. Multiple applications of Alamar Blue as an indicator of metabolic function and cellular health in cell viability bioassays. Sensors (Basel). (2012) 12(9):12347-60.
2. Clerkin JS, Naughton R, Quiney C, Cotter TG. Mechanisms of ROS modulated cell survival during carcinogenesis. Cancer Lett. (2008) 266(1):30-6.
3. Giles FJ, Shi GG, Cortes JE, Thomas D, Keating AR, Kantarjian HM, et al. Amifostine does not reduce the toxicity of the fludarabine and cyclophosphamide regimen in patients with chronic lymphocytic leukemia. Cancer Chemother Pharmacol. (2003) 52(3):223-8.

Supplementary Figure 6A-F. Synergy reports for human cancer cell lines treated with 4SP65, cisplatin, or both drugs in combination. SynergyFinder 2.0 (https://synergyfinder.fimm.fi) was used to produce dose-response curves for 4SP65 (4SP) and cisplatin (DDP), a dose-response matrix for growth inhibition, a heat map showing clustering of degrees of synergism, and a volcano plot showing the distribution of synergy strength, and to derive synergy scores following single drug and combination drug treatments of (A) A459 cells, (B) H460 cells, (C) HMESO1 cells, (D) PPM-Mill cells, (E) SKOV3 cells, and (F) TOV21G cells for 48 h. Note that clicking on the volcano plot in each synergy report activates a 3-D animation of the distribution of synergy strength.

Figure S6A. Treatment of A549 cells

Calculation and Visualization of synergy scores for Drug Combinations

Drug combinations:

Drug combination	Synergy score	Most synergistic area score	Method
DDP - 4SP	23.56	29.79	HSA

Chosen parameters:

Readout: inhibition ; Baseline correction: Yes ;

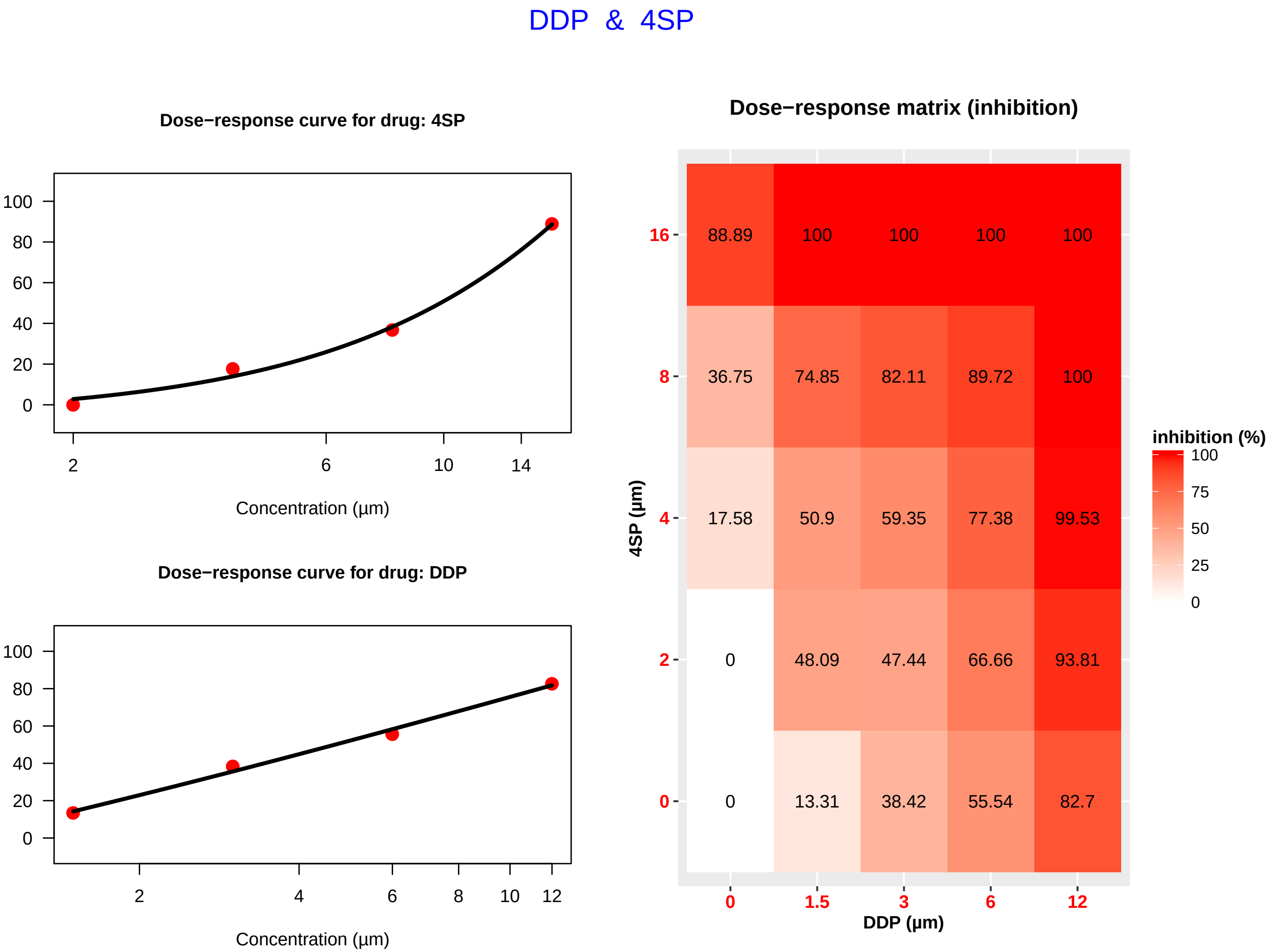
DDP & 4SP
Dose−response curve for drug: 4SP
0 20 40 60 80 100
2 6 10 14
Concentration (µm)
Dose−response curve for drug: DDP
0 20 40 60 80 100
2 4 6 8 10 12
Concentration (µm)
Dose−response matrix (inhibition)
16 8 4 2 0
88.89 100 100 100 100
36.75 74.85 82.11 89.72 100
17.58 50.9 59.35 77.38 99.53
0 48.09 47.44 66.66 93.81
0 13.31 38.42 55.54 82.7
0 1.5 3 6 12
4SP (µm)
DDP (µm)
inhibition (%)
100 75 50 25 0

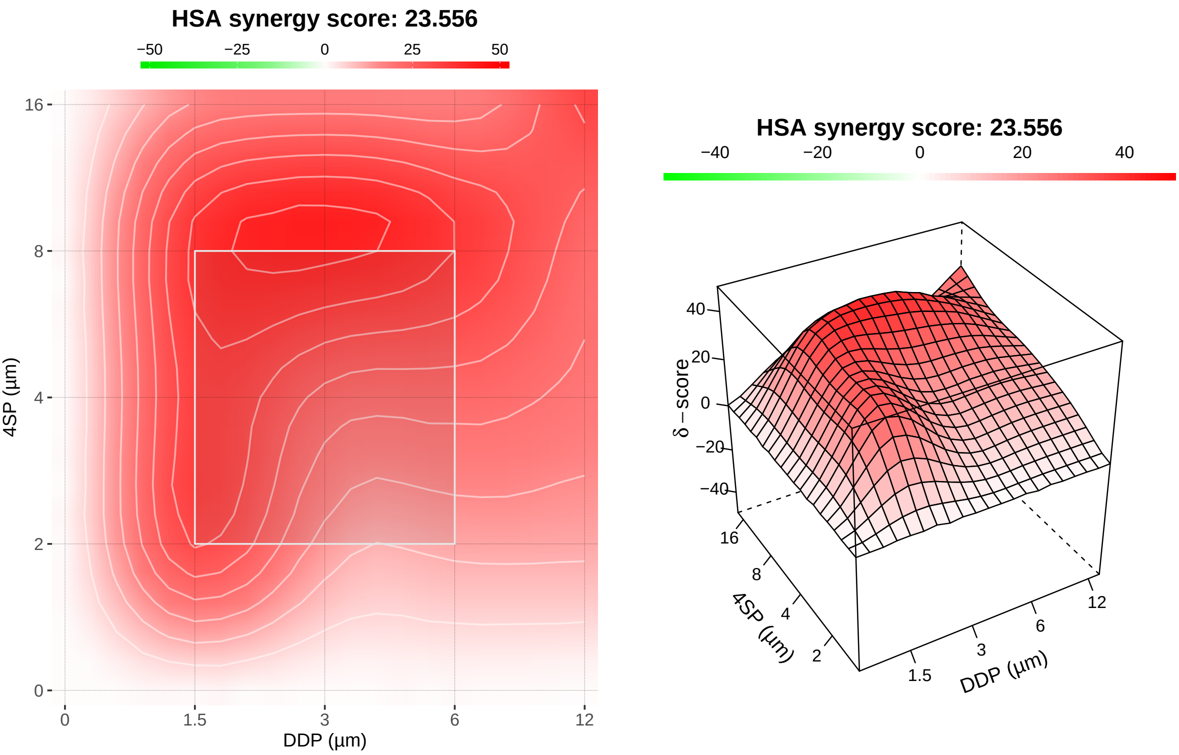
HSA synergy score: 23.556
−50
−25
0
25
50
16
8
4
2
0
4SP (µm)
0
1.5
3
6
12
DDP (µm)
HSA synergy score: 23.556
−40
−20
0
20
40
δ − score
40
20
0
−20
−40
16
8
4
2
4SP (µm)
1.5
3
6
12
DDP (µm)

Figure S6B. Treatment of H460 cells

Calculation and Visualization of synergy scores for Drug Combinations

Drug combinations:

Drug combination	Synergy score	Most synergistic area score	Method
DDP - 4SP	36.38	57.54	HSA

Chosen parameters:

Readout: inhibition ; Baseline correction: Yes ;

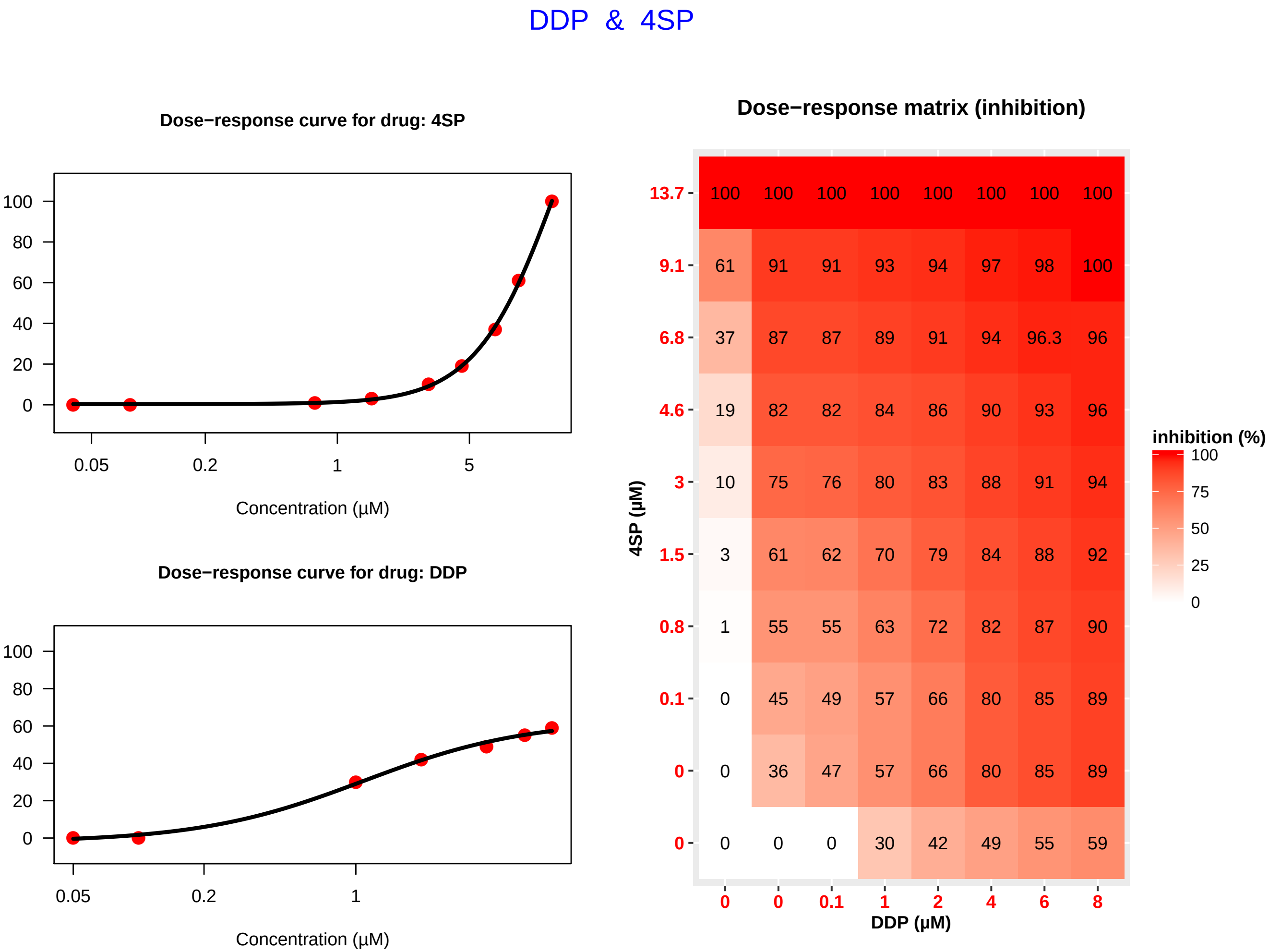
DDP & 4SP
Dose−response curve for drug: 4SP
Concentration (µM)
Dose−response curve for drug: DDP
Concentration (µM)
Dose−response matrix (inhibition)
4SP (µM)
DDP (µM)
inhibition (%)
13.7: 100 100 100 100 100 100 100 100
9.1: 61 91 91 93 94 97 98 100
6.8: 37 87 87 89 91 94 96.3 96
4.6: 19 82 82 84 86 90 93 96
3: 10 75 76 80 83 88 91 94
1.5: 3 61 62 70 79 84 88 92
0.8: 1 55 55 63 72 82 87 90
0.1: 0 45 49 57 66 80 85 89
0: 0 36 47 57 66 80 85 89
0: 0 0 0 30 42 49 55 59
0 0 0.1 1 2 4 6 8

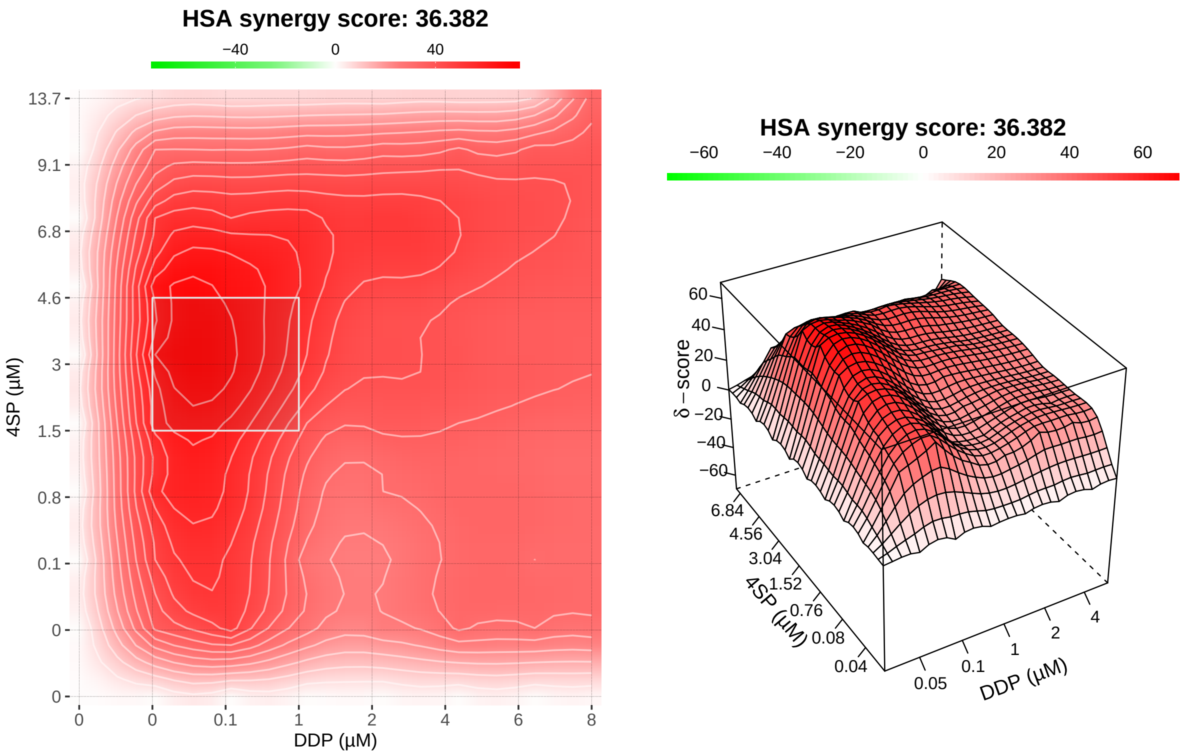
HSA synergy score: 36.382
−40
0
40
13.7
9.1
6.8
4.6
3
1.5
0.8
0.1
0
0
4SP (μM)
0
0
0.1
1
2
4
6
8
DDP (μM)
HSA synergy score: 36.382
−60
−40
−20
0
20
40
60
60
40
20
0
−20
−40
−60
δ − score
6.84
4.56
3.04
1.52
0.76
0.08
0.04
4SP (μM)
0.05
0.1
1
2
4
DDP (μM)

Figure S6C. Treatment of HMESO1 cells

Calculation and Visualization of synergy scores for Drug Combinations

Drug combinations:

Drug combination	Synergy score	Most synergistic area score	Method
DDP - 4SP	13.10	36.10	HSA

Chosen parameters:

Readout: inhibition ; Baseline correction: Yes ;

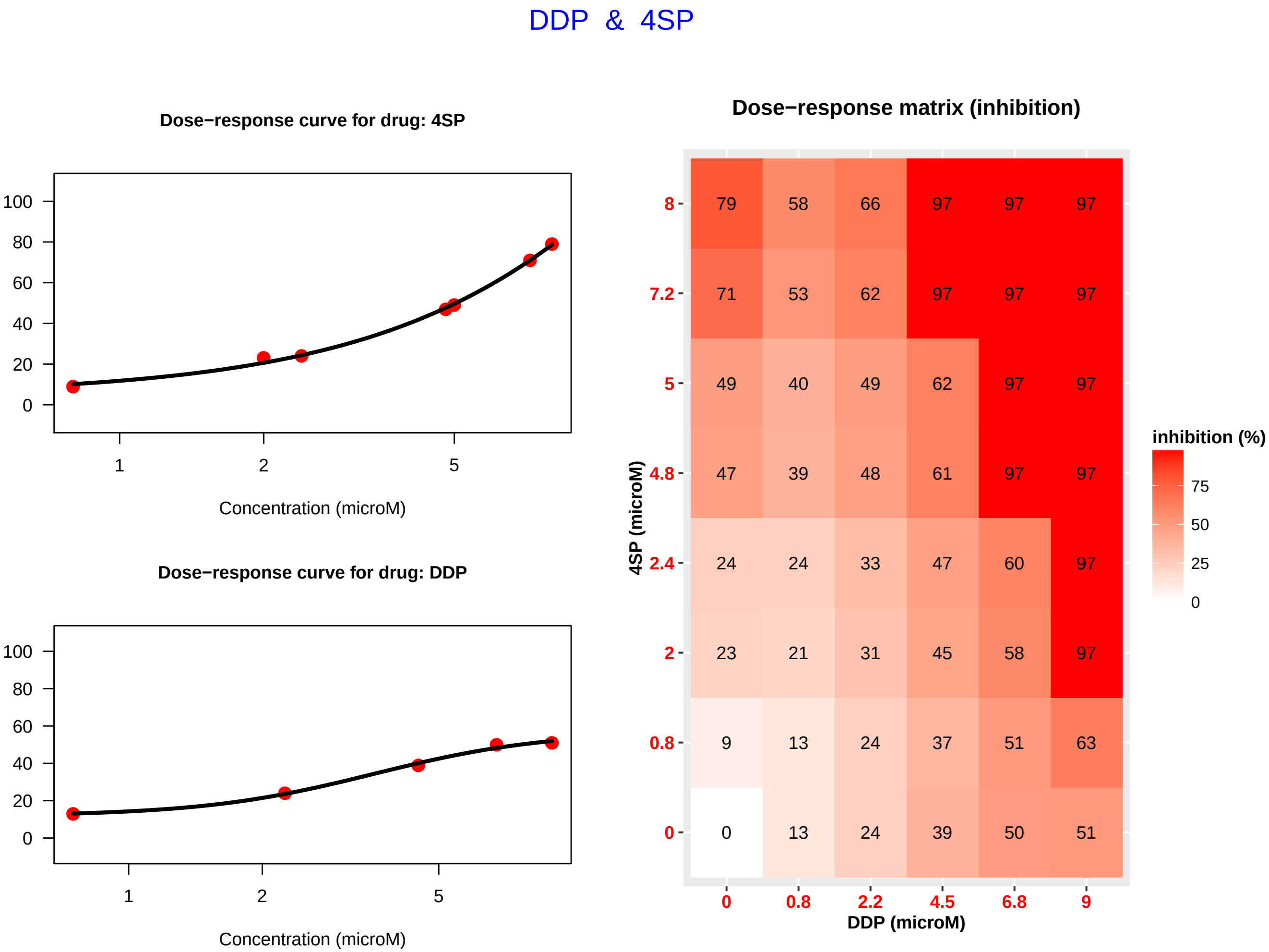
DDP & 4SP
Dose−response curve for drug: 4SP
100
80
60
40
20
0
1
2
5
Concentration (microM)
Dose−response curve for drug: DDP
100
80
60
40
20
0
1
2
5
Concentration (microM)
Dose−response matrix (inhibition)
4SP (microM)
8
7.2
5
4.8
2.4
2
0.8
0
79 58 66 97 97 97
71 53 62 97 97 97
49 40 49 62 97 97
47 39 48 61 97 97
24 24 33 47 60 97
23 21 31 45 58 97
9 13 24 37 51 63
0 13 24 39 50 51
0
0.8
2.2
4.5
6.8
9
DDP (microM)
inhibition (%)
75
50
25
0

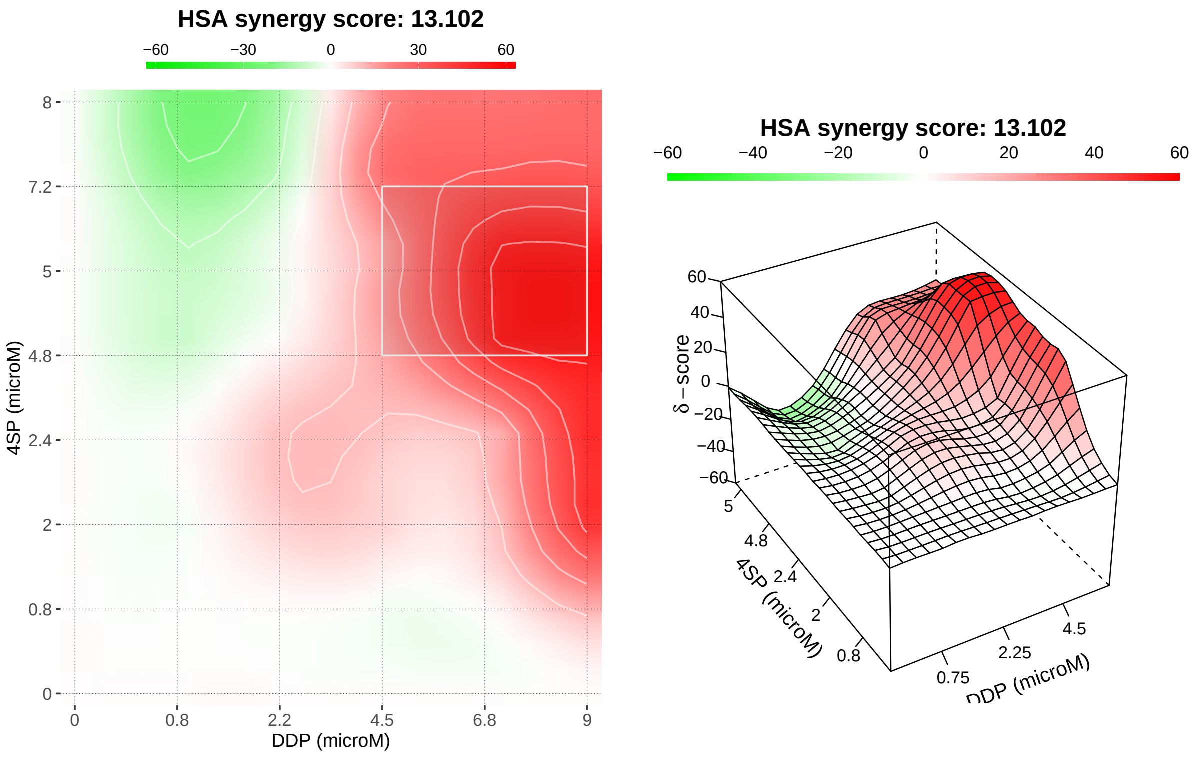
HSA synergy score: 13.102
−60
−30
0
30
60
4SP (microM)
8
7.2
5
4.8
2.4
2
0.8
0
DDP (microM)
0
0.8
2.2
4.5
6.8
9
HSA synergy score: 13.102
−60
−40
−20
0
20
40
60
δ − score
60
40
20
0
−20
−40
−60
4SP (microM)
5
4.8
2.4
2
0.8
DDP (microM)
0.75
2.25
4.5

Figure S6D. Treatment of PPM-Mill cells

Calculation and Visualization of synergy scores for Drug Combinations

Drug combinations:

Drug combination	Synergy score	Most synergistic area score	Method
DDP - 4SP	5.82	20.78	HSA

Chosen parameters:

Readout: inhibition ; Baseline correction: Yes ;

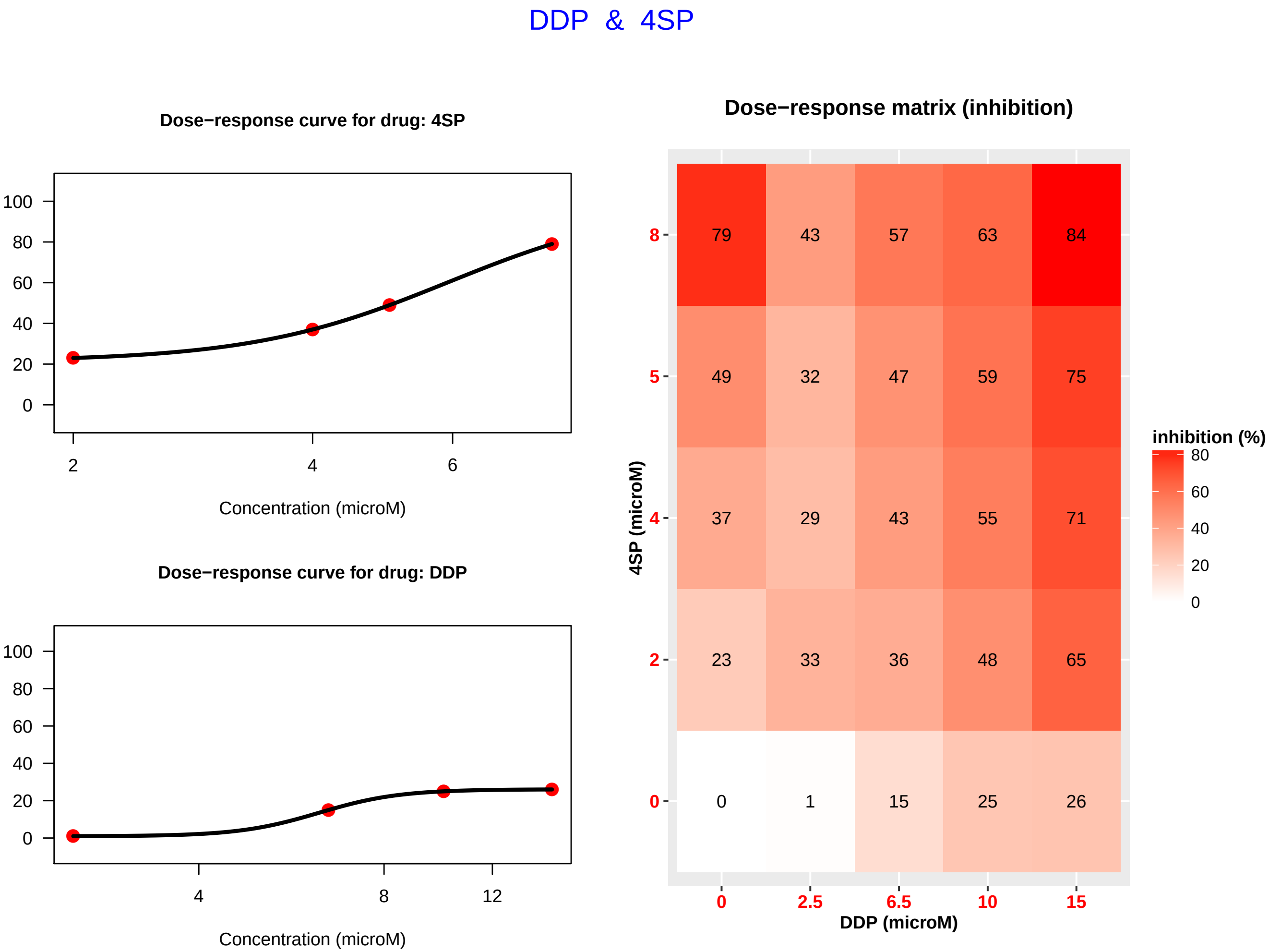

DDP & 4SP
Dose−response curve for drug: 4SP
100
80
60
40
20
0
2
4
6
Concentration (microM)
Dose−response curve for drug: DDP
100
80
60
40
20
0
4
8
12
Concentration (microM)
Dose−response matrix (inhibition)
4SP (microM)
8
5
4
2
0
79 43 57 63 84
49 32 47 59 75
37 29 43 55 71
23 33 36 48 65
0 1 15 25 26
0
2.5
6.5
10
15
DDP (microM)
inhibition (%)
80
60
40
20
0

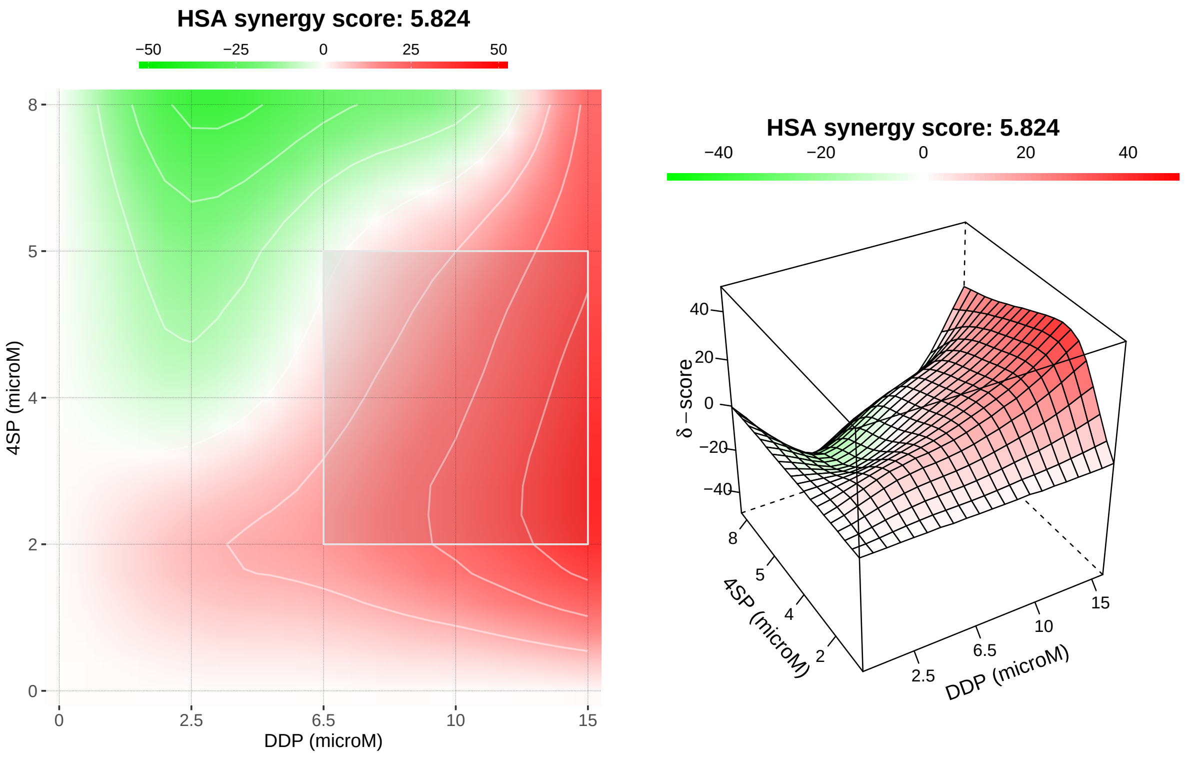
HSA synergy score: 5.824
−50
−25
0
25
50
8
5
4
2
0
0
2.5
6.5
10
15
DDP (microM)
4SP (microM)
HSA synergy score: 5.824
−40
−20
0
20
40
δ − score
40
20
0
−20
−40
8
5
4
2
4SP (microM)
2.5
6.5
10
15
DDP (microM)

Figure S6E. Treatment of SKOV3 cells

Calculation and Visualization of synergy scores for Drug Combinations

Drug combinations:

Drug combination	Synergy score	Most synergistic area score	Method
DDP - 4SP	18.16	42.09	HSA

Chosen parameters:

Readout: inhibition ; Baseline correction: Yes ;

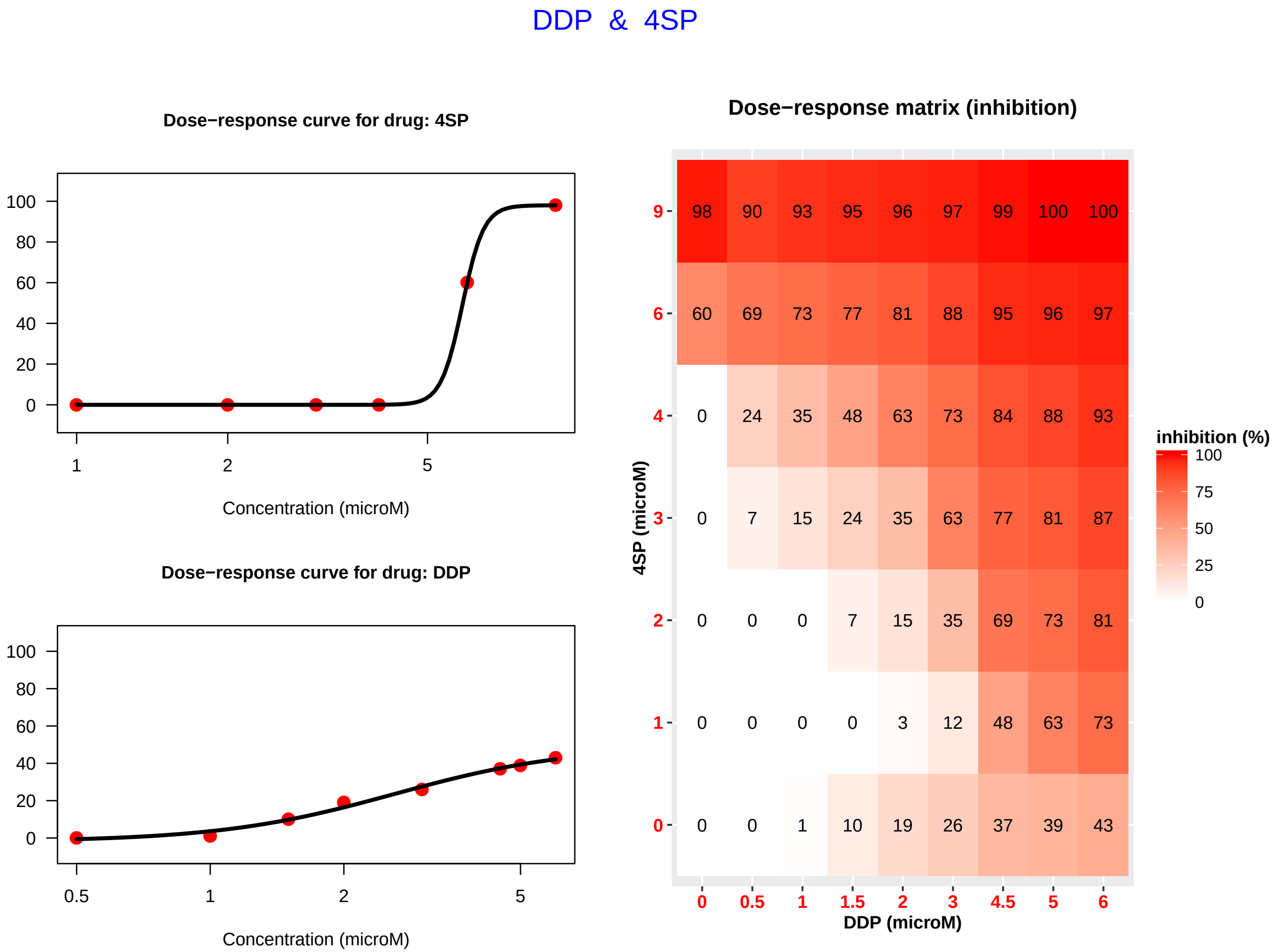
DDP & 4SP
Dose−response curve for drug: 4SP
100
80
60
40
20
0
1
2
5
Concentration (microM)
Dose−response curve for drug: DDP
100
80
60
40
20
0
0.5
1
2
5
Concentration (microM)
Dose−response matrix (inhibition)
9: 98 90 93 95 96 97 99 100 100
6: 60 69 73 77 81 88 95 96 97
4: 0 24 35 48 63 73 84 88 93
3: 0 7 15 24 35 63 77 81 87
2: 0 0 0 7 15 35 69 73 81
1: 0 0 0 0 3 12 48 63 73
0: 0 0 1 10 19 26 37 39 43
4SP (microM)
0 0.5 1 1.5 2 3 4.5 5 6
DDP (microM)
inhibition (%)
100
75
50
25
0

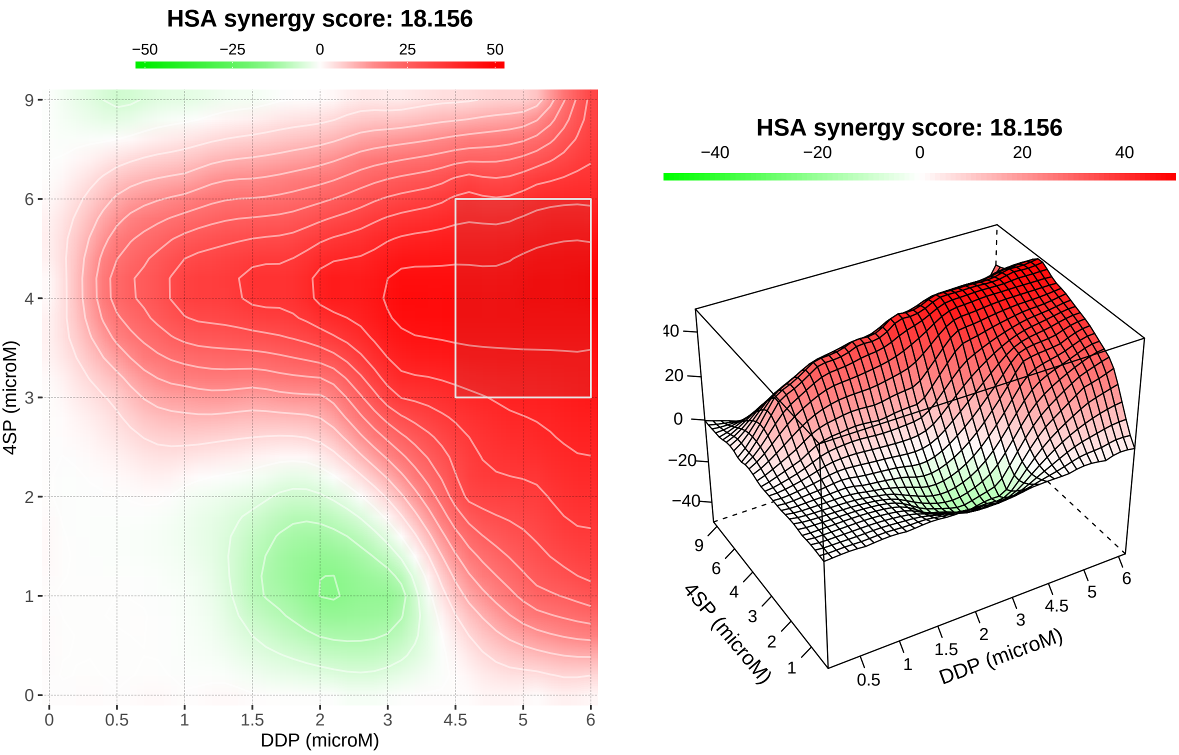
HSA synergy score: 18.156
−50
−25
0
25
50
4SP (microM)
DDP (microM)
HSA synergy score: 18.156
−40
−20
0
20
40
4SP (microM)
DDP (microM)

Figure S6F. Treatment of SKOV3 cells

Calculation and Visualization of synergy scores for Drug Combinations

Drug combinations:

Drug combination	Synergy score	Most synergistic area score	Method
DDP - 4SP	18.22	26.43	HSA

Chosen parameters:

Readout: inhibition ; Baseline correction: Yes ;

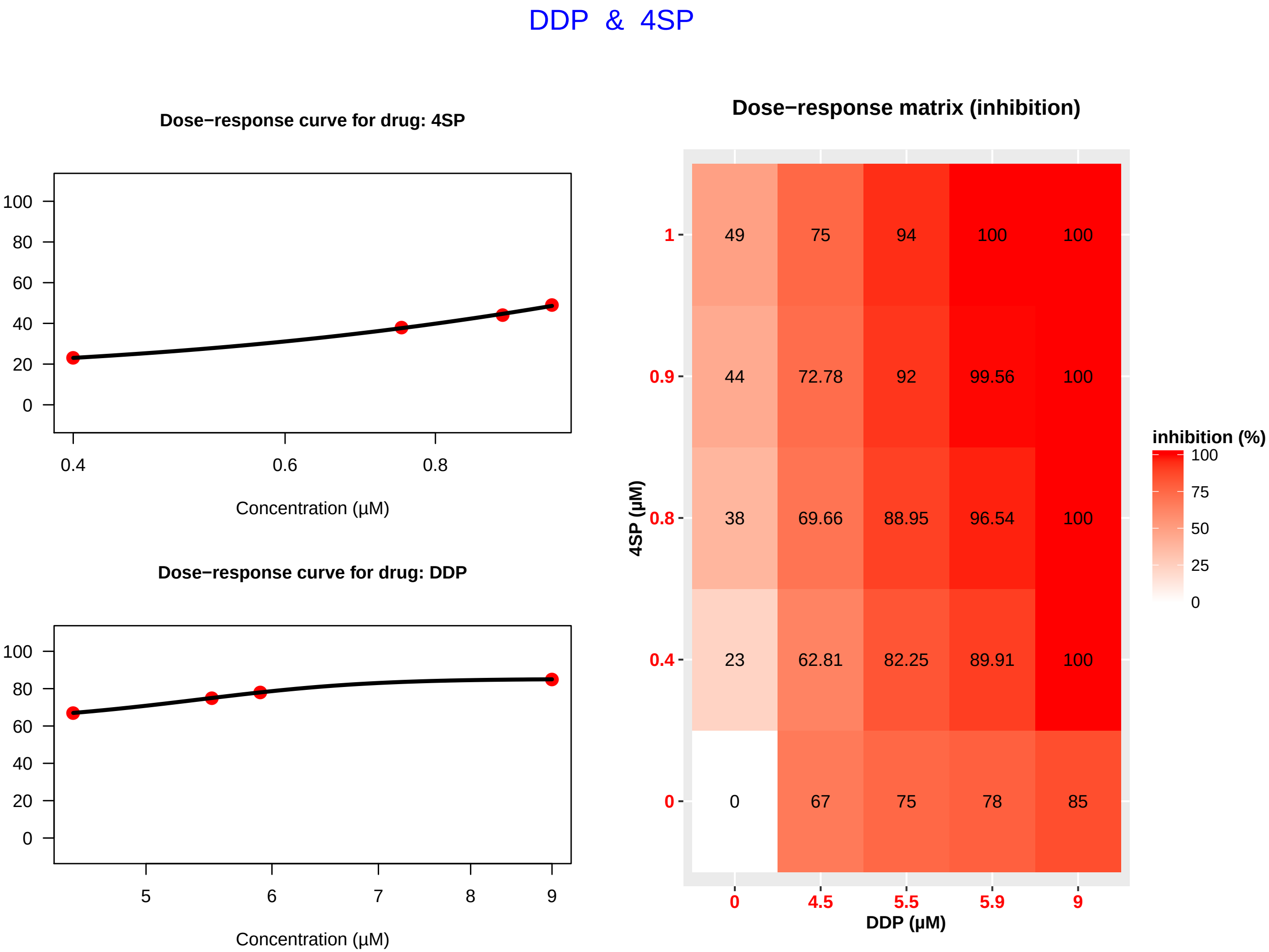
DDP & 4SP
Dose−response curve for drug: 4SP
Concentration (μM)
Dose−response curve for drug: DDP
Concentration (μM)
Dose−response matrix (inhibition)
4SP (μM)
DDP (μM)
inhibition (%)
49 75 94 100 100
44 72.78 92 99.56 100
38 69.66 88.95 96.54 100
23 62.81 82.25 89.91 100
0 67 75 78 85

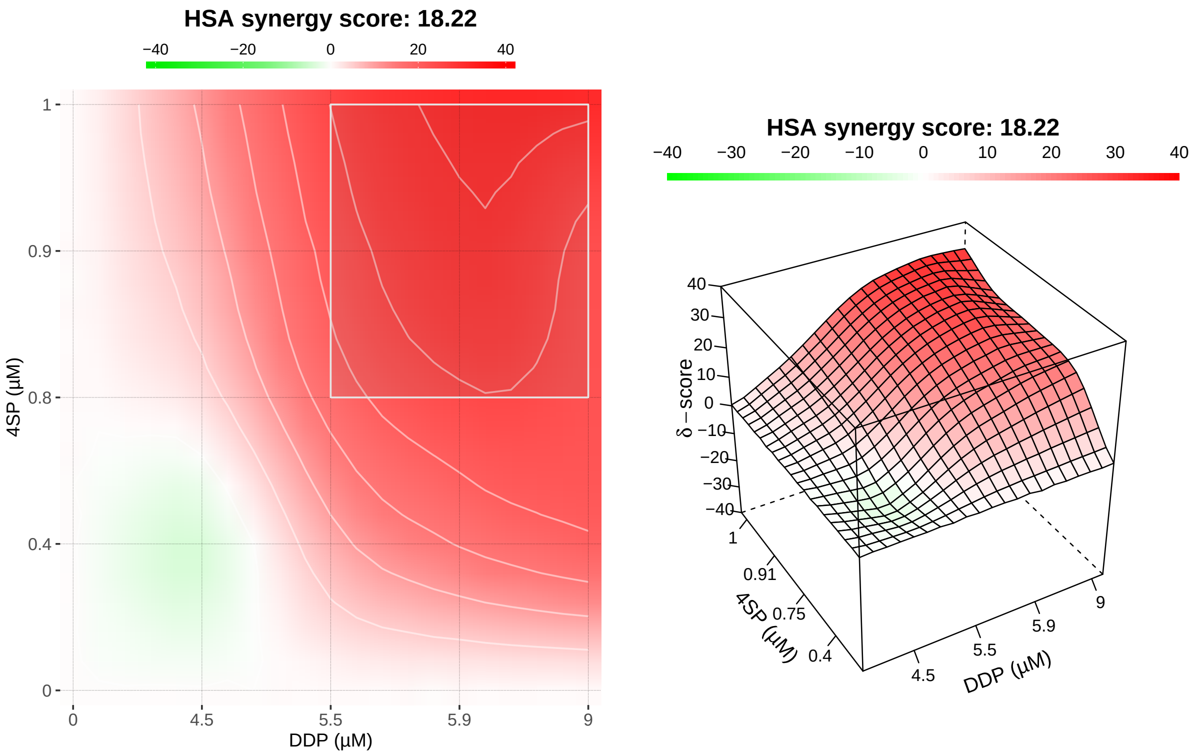
HSA synergy score: 18.22
−40
−20
0
20
40
4SP (μM)
1
0.9
0.8
0.4
0
DDP (μM)
0
4.5
5.5
5.9
9
HSA synergy score: 18.22
−40
−30
−20
−10
0
10
20
30
40
δ − score
40
30
20
10
0
−10
−20
−30
−40
4SP (μM)
1
0.91
0.75
0.4
DDP (μM)
4.5
5.5
5.9
9

Supplementary Figure 7. Synergy report for A549 non-small lung cancer cells treated with 4SP65, gefitinib, or both drugs in combination. SynergyFinder 2.0 (https://synergyfinder.fimm.fi) was used to produce dose-response curves for 4SP65 (4SP) and gefitinib (GEF), a dose-response matrix for growth inhibition, a heat map showing clustering of degrees of synergism, and a volcano plot showing the distribution of synergy strength, and to derive synergy scores following single drug and combination drug treatments of A549 cells for 48 h. Note that clicking on the volcano plot in each synergy report activates a 3-D animation of the distribution of synergy strength.

Figure S7. Treatment of A549 cells

Calculation and Visualization of synergy scores for Drug Combinations

Drug combinations:

Drug combination	Synergy score	Most synergistic area score	Method
GEF - 4SP	14.65	20.51	HSA

Chosen parameters:

Readout: inhibition ; Baseline correction: Yes ;

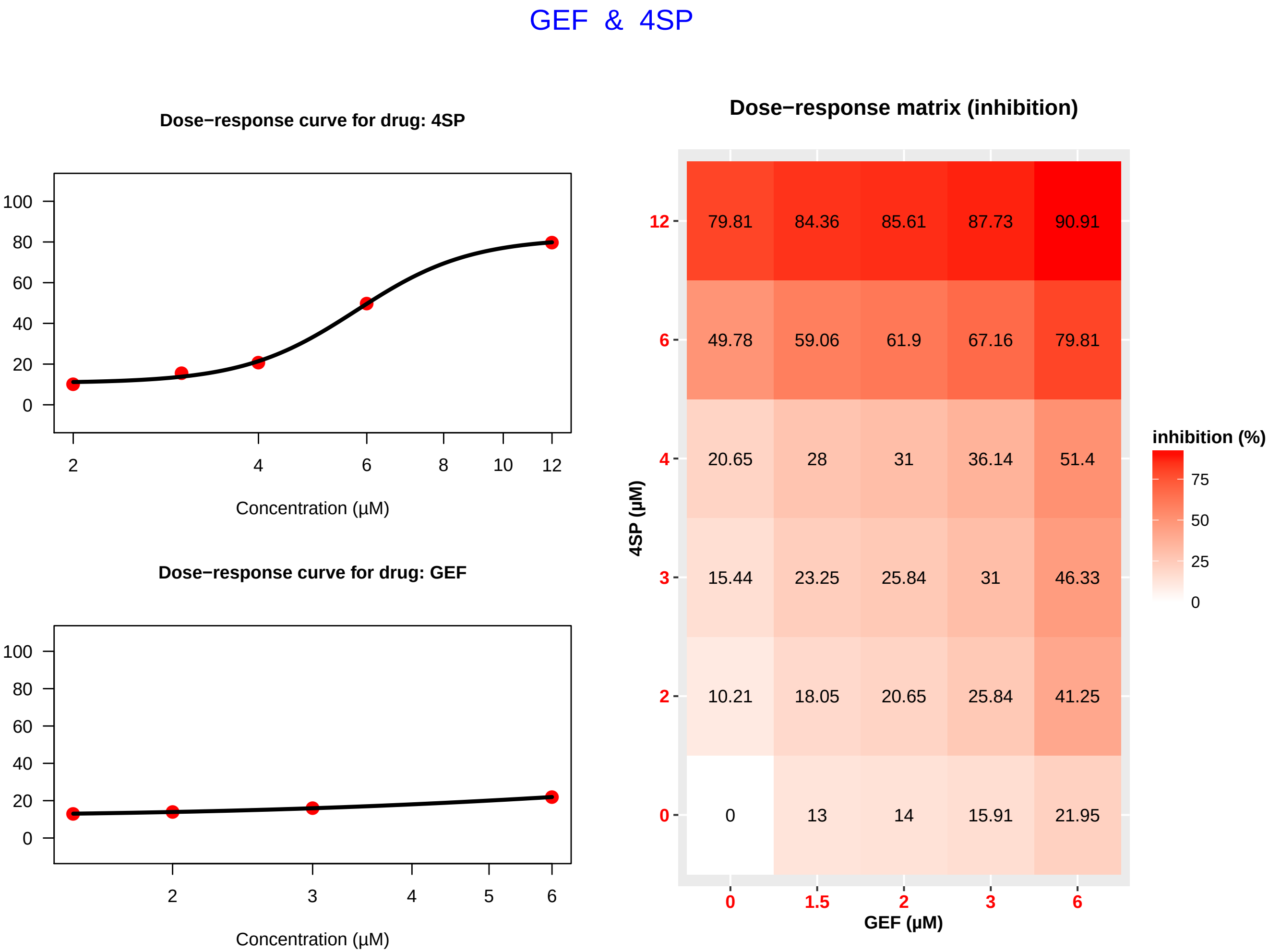
GEF & 4SP
Dose−response curve for drug: 4SP
100
80
60
40
20
0
2
4
6
8
10
12
Concentration (µM)
Dose−response curve for drug: GEF
100
80
60
40
20
0
2
3
4
5
6
Concentration (µM)
Dose−response matrix (inhibition)
4SP (µM)
12
6
4
3
2
0
79.81 84.36 85.61 87.73 90.91
49.78 59.06 61.9 67.16 79.81
20.65 28 31 36.14 51.4
15.44 23.25 25.84 31 46.33
10.21 18.05 20.65 25.84 41.25
0 13 14 15.91 21.95
0
1.5
2
3
6
GEF (µM)
inhibition (%)
75
50
25
0

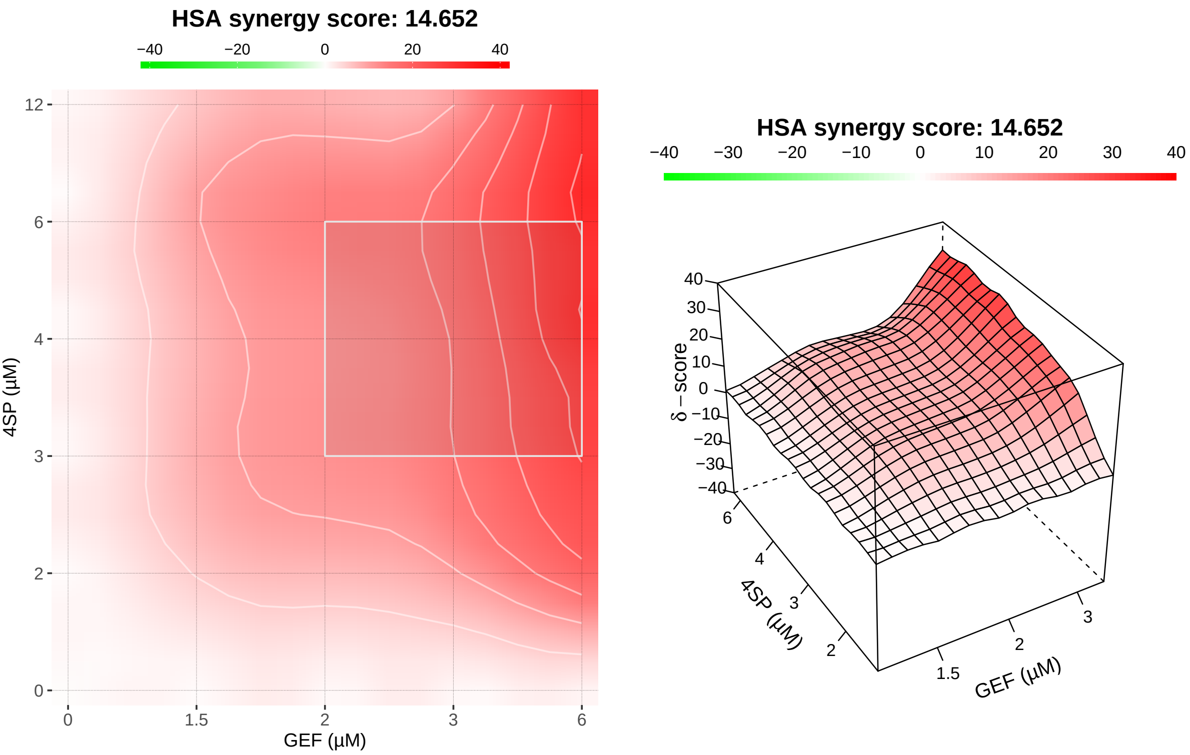
HSA synergy score: 14.652
−40
−20
0
20
40
12
6
4
3
2
0
4SP (μM)
0
1.5
2
3
6
GEF (μM)
HSA synergy score: 14.652
−40
−30
−20
−10
0
10
20
30
40
δ − score
40
30
20
10
0
−10
−20
−30
−40
6
4
3
2
4SP (μM)
1.5
2
3
GEF (μM)

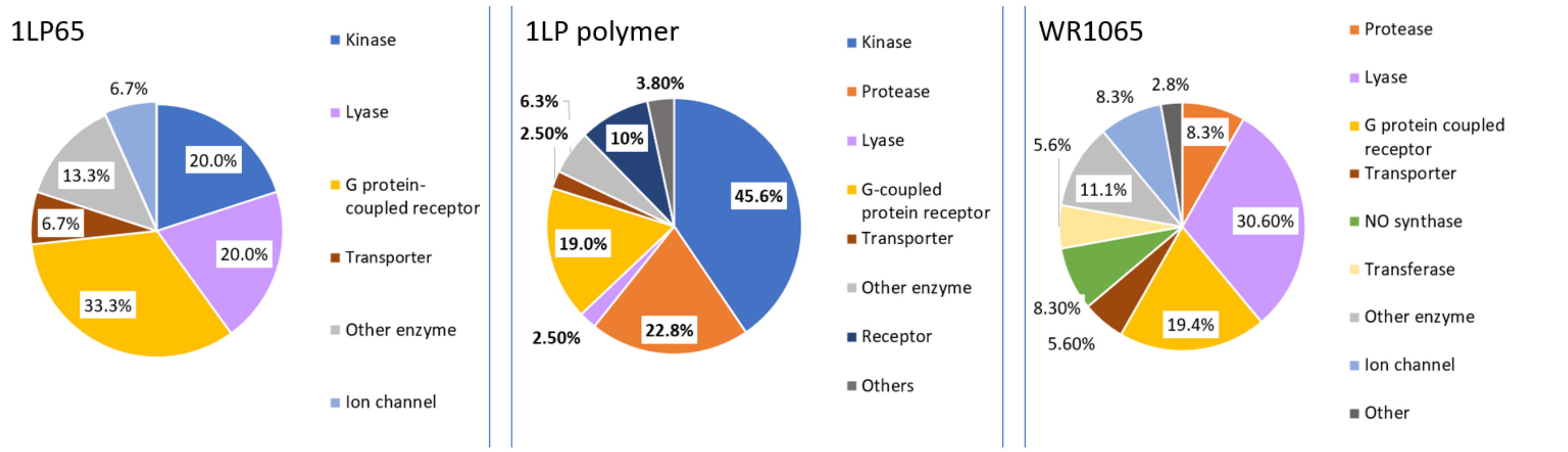

Supplementary Figure 8. Pie charts showing the relative distribution of target molecules based upon SwissADME modeling (Daina et al 2017). There were 30 predicted targets for mPEG$_6$-S-S-WR1065 (1LP65), 100 for mPEG$_6$-SH (1LP polymer), and 36 for WR1065. Note that miscellaneous enzymes with low probability of activity as targets are grouped under 'other enzyme'. The finding of many predicted targets for 1LP polymer, with over 50% categorized as kinases, proteases, or lyases offers potential insight into the anticancer modes of action of 1LP65 compared to amifostine. Also note that no predictions for 4-star-PEG-S-S-WR1065 (4SP65) or 4-arm PEG-SH (4SP65 polymer) are available because the Swiss ADME program only evaluates small molecules. Based upon the similarity in structure between the 1LP polymer and one arm of 4SP polymer, it is postulated that predicted targets are similar.

Daina A, Michielin O, Zoete V. SwissADME: a free web tool to evaluate pharmacokinetics, drug-likeness and medicinal chemistry friendliness of small molecules. Scientific reports. (2017) 7:42717.

Supplementary Table 1. Swiss ADME web tool (Daina et al 2017) predicted targets for mPEG$_6$-S-S-WR1065 (1LP65), mPEG$_6$-SH (1LP polymer), and WR1065

**Compound**	***Target***	***Common name***	***Target Class***
**1LP65**			
	3-phosphoinositide dependent protein kinase-1	PDPK1	Kinase
	Inhibitor of NF-kB kinase beta subunit	IKBKB	Kinase
	PI3-kinase p110-alpha subunit	PIK3CA	Kinase
	Serine/threonine-protein kinase Aurora-A	AURKA	Kinase
	Serine/threonine-protein kinase Aurora-B	AURKB	Kinase
	Serine/threonine-protein kinase mTOR	MTOR	Kinase
	Carbonic anhydrase I	CA1	Lyase
	Carbonic anhydrase II	CA2	Lyase
	Carbonic anhydrase IV	CA4	Lyase
	Carbonic anhydrase IX	CA9	Lyase
	Carbonic anhydrase XII	CA12	Lyase
	Carbonic anhydrase XIV	CA14	Lyase
	Adenosine A1 receptor	ADORA1	Family A G protein-coupled receptor
	Adenosine A2a receptor	ADORA2A	Family A G protein-coupled receptor
	Adenosine A3 receptor	ADORA3	Family A G protein-coupled receptor
	Beta-1 adrenergic receptor	ADRB1	Family A G protein-coupled receptor
	Beta-3 adrenergic receptor	ADRB3	Family A G protein-coupled receptor
	Dopamine D2 receptor	DRD2	Family A G protein-coupled receptor
	Dopamine D3 receptor	DRD3	Family A G protein-coupled receptor
	Muscarinic acetylcholine receptor M1	CHRM1	Family A G protein-coupled receptor
	Muscarinic acetylcholine receptor M3	CHRM3	Family A G protein-coupled receptor
	Serotonin 4 (5-HT4) receptor	HTR4	Family A G protein-coupled receptor
	ATP-binding cassette sub-family G member 2		Transporter - primary active
	Sodium/potassium transporting ATPase alpha-1 chain	ATP1A1	Transporter - primary active
	Acetylcholinesterase	ACHE	Other enzyme -hydrolase
	Beta-galactosidase	GLB1	Other enzyme -hydrolase
	Butyrylcholinesterase	ACHE	Other enzyme -hydrolase
	Phosphodiesterase 4D	PDE4D	Other enzyme - phosphodiesterase
	Apoptosis regulator Bcl-2	BCL2	Other ion channel
	Apoptosis regulator Bcl-x	BCL2L1	Other ion channel
**1LP polymer**			
	c-Jun N-terminal kinase 1	MAPK8	Kinase
	c-Jun N-terminal kinase 3	MAPK10	Kinase
	Cyclin-dependent kinase 2/cyclin E	CCNE2<br>CDK2<br>CCNE1	Kinase
	Cyclin-dependent kinase 5/CDK5 activator 1	CDK5R1<br>CDK5	Kinase
	Dual specificity mitogen-activated protein kinase kinase 1	MAP2K1	Kinase
	Epidermal growth factor receptor erbB1	EGFR	Kinase

	Fibroblast growth factor receptor 1	FGFR1	Kinase
	Fibroblast growth factor receptor 2	FGFR2	Kinase
	Fibroblast growth factor receptor 3	FGFR3	Kinase
	Hepatocyte growth factor receptor	MET	Kinase
	Inhibitor of NF-Kb kinase beta subunit	IKBKB	Kinase
	Inhibitor of NF-kB kinase epsilon subunit	IKBKE	Kinase
	Interleukin-1 receptor-associated kinase 4	IRAK4	Kinase
	Kinesin-1 heavy chain/Tyrosine-protein kinase receptor RET	RET	Kinase
	Macrophage colony stimulating factor receptor	CSF1R	Kinase
	Macrophage-stimulating protein receptor	MST1R	Kinase
	MAP kinase ERK2	MAPK1	Kinase
	Nerve growth factor receptor Trk-A	NTRK1	Kinase
	Neurotropic tyrosine kinase receptor type 2	NTRK2	Kinase
	PI2-kinase p110-alpha subunit	PIK3CB	Kinase
	PI2-kinase p110-beta subunit	PIK3CB	Kinase
	Platelet-derived growth factor receptor beta	PDGFRB	Kinase
	Proto-oncogene tyrosine protein kinase MER	MERTK	Kinase
	Serine/threonine-protein kinase Aurora-A	AURKA	Kinase
	Serine/threonine-protein kinase Aurora-B	AURKB	Kinase
	Serine/threonine-protein kinase Chk1	CHEK1	Kinase
	Serine/threonine-protein kinase mTOR (by homology)	TBK1	Kinase
	Serine/threonine-protein kinase TBK1 (by homology)	MTOR	Kinase
	Thymidine kinase (mitochondrial)	TK2	Kinase
	Tyrosine kinase receptor FLT3	FLT3	Kinase
	Tyrosine-protein kinase ABL	ABL1	Kinase
	Tyrosine-protein kinase JAK2	JAK2	Kinase
	Tyrosine-protein kinase SRC	SRC	Kinase
	Vascular endothelial growth factor receptor 1	FLT1	Kinase
	Vascular endothelial growth factor receptor 2	KDR	Kinase
	Vascular endothelial growth factor receptor 3	FLT4	Kinase
	Angiotensin-converting enzyme	ACE	Protease
	Bone morphogenetic protein 1	BMP1	Protease
	Caspase-1	CASP1	Protease
	Caspase-3	CASP3	Protease
	Caspase-7	CASP7	Protease
	Cathepsin S	CTSS	Protease
	Endothelin-converting enzyme 1	ECE1	Protease
	Matrix metalloproteinase 1	MMP1	Protease
	Matrix metalloproteinase 2	MMP2	Protease
	Matrix metalloproteinase 3	MMP3	Protease
	Matrix metalloproteinase 7	MMP7	Protease
	Matrix metalloproteinase 8	MMP8	Protease
	Matrix metalloproteinase 9	MMP9	Protease
	Matrix metalloproteinase 12	MMP12	Protease
	Matrix metalloproteinase 13	MMP13	Protease
	Matrix metalloproteinase 14	MMP14	Protease
	Neprilysin	MME	Protease
	Thrombin and coagulation factor X	F10	Protease

	Carbonic anhydrase II	CA2	Lyase
	Carbonic anhydrase VII	CA7	Lyase
	Adenosine A1 receptor	ADORA1	Family A G-protein coupled receptor
	Adenosine A2a receptor	ADORA2A	Family A G-protein coupled receptor
	Adenosine A2b receptor	ADORA2B	Family A G-protein coupled receptor
	Adenosine A3 receptor	ADORA3	Family A G-protein coupled receptor
	Angiotensin II receptor	AGTR2	Family A G-protein coupled receptor
	C-C chemokine receptor type 4	CCR4	Family A G-protein coupled receptor
	Cysteinyl leukotriene receptor 1	CYSLTR1	Family A G-protein coupled receptor
	Endothelin receptor ET-A (by homology)	EDNRA	Family A G-protein coupled receptor
	Hydroxycarboxylic acid receptor 2	HCAR2	Family A G-protein coupled receptor
	Melanin-concentrating hormone receptor 1	MCHR1	Family A G-protein coupled receptor
	Neurokinin 2 receptor	TACR2	Family A G-protein coupled receptor
	Neurotensin receptor 1	NTSR1	Family A G-protein coupled receptor
	Prostanoid EP4 receptor	PTGER4	Family A G-protein coupled receptor
	Type-1 angiotensin II receptor	AGTR1	Family A G-protein coupled receptor
	Growth hormone-releasing hormone receptor	GHRHR	Family B G-protein coupled receptor
	11-beta-hydroxiysteroid dehydrogenase 1 (by homology)	HSD11B1	Other enzyme - dehydrogenase
	Glyceraldehyde-3-phosphate dehydrogenase	GADPH	Other enzyme -oxidoreductase
	Liver glycogen phosphorylase	PYGL	Other enzyme - phosphorylase
	Protein-tyrosine phosphatase 1B	PTPN1	Other enzyme - phosphatase
	Phosphodiesterase 5A	PDE5A	Other enzyme - phosphodiesterase
	Diacylglycerol O-acyltransferase 1	DGAT1	Transferase
	Nicotinamide phosphoribosyltransferase	NAMPT	Transferase
	Integrin alpha-4/beta-1	ITGB1	Receptor - membrane
	Integrin alpha-4/beta-7	ITGB7<br>ITGA4	Receptor - membrane
	Integrin alpha-IIb/beta-3	ITGA2B<br>ITGB3	Receptor - membrane
	Integrin alpha-V/beta-3	ITGAV	Receptor - membrane
	Integrin alpha-V/beta-5	ITGB5<br>ITGAV	Receptor - membrane
	Integrin alpha-V/beta-6	ITGAV<br>ITGB6	Receptor - membrane
	Intercellular adhesion molecule (ICAM-1), Integrin alpha-L/beta-2	ITGAL<br>ICAM1<br>ITGB2	Receptor - membrane
	Integrin alpha-4	ITGA4	Receptor - membrane
	Peroxisome proliferator-activated receptor alpha	PPARA	Receptor - nuclear
	Peroxisome proliferator-activated receptor delta	PPARD	Receptor - nuclear
	Peroxisome proliferator-activated receptor gamma	PPARG	Receptor - nuclear
	Equilibrative nucleoside transporter 1	SLC29A1	Transporter (electrochemical)
	Sodium/glucose cotransporter 1	SLC5A1	Transporter (electrochemical)
	Sodium/glucose cotransporter 2	SLC5A2	Transporter (electrochemical)
	Galectin-1	LGALS1	Protein - other cytosolic
	Galectin-3	LGALS3	Protein - other cytosolic

	Induced myeloid leukemia cell differentiation protein Mcl-1	MCL1	Protein - other cytosolic
	Transmembrane domain containing protein TMIGD3	TMIGD3	Protein - unclassified
	Apoptosis regulator Bcl-X		Ion channel - other
	Cholesteryl ester transfer protein	CETP	Ion channel - other
	HERG	KCNH2	Ion channel - voltage-gated
	Cytochrome P450 3A4	CYP3A4	Cytochrome P450
**WR1065**			
	Carbonic anhydrase I	CA1	Lyase
	Carbonic anhydrase II	CA2	Lyase
	Carbonic anhydrase IV	CA4	Lyase
	Carbonic anhydrase 5A	CA5A	Lyase
	Carbonic anhydrase 5B	CA5B	Lyase
	Carbonic anhydrase VI	CA6	Lyase
	Carbonic anhydrase VII	CA7	Lyase
	Carbonic anhydrase IX	CA9	Lyase
	Carbonic anhydrase XII	CA12	Lyase
	Carbonic anhydrase XIII (by homology)	CA13	Lyase
	Carbonic anhydrase XIV	CA14	Lyase
	Alpha-2a adrenergic receptor	ADRA2A	Family A G protein – coupled receptor
	Dopamine D2 receptor	DRD2	Family A G protein – coupled receptor
	Dopamine D3 receptor	DRD3	Family A G protein – coupled receptor
	Histamine H2 receptor	HRH2	Family A G protein – coupled receptor
	Histamine H3 receptor	HRH3	Family A G protein – coupled receptor
	Histamine H4 receptor	HRH4	Family A G protein – coupled receptor
	Serotonin 1b (5-HT1b) receptor (by homology)	HTR1B	Family A G protein – coupled receptor
	Nitric-oxide synthase, brain	NOS1	NO synthase enzyme
	Nitric-oxide synthase, endothelial	NOS3	NO synthase enzyme
	Nitric-oxide synthase, inducible	NOS2	NO synthase enzyme
	Phenylethanolamine N-methyltransferase	PMNT	Transferase enzyme
	Histone acetyltransferase PCAF	KAT2B	Transferase (writer)
	Neuronal acetylcholine receptor, alpha3/beta4	CHRNA3 CHRNB4	Ligand-gated ion channel
	Neuronal acetylcholine receptor protein alpha4/beta2	CHRNA4 CHRNB2	Ligand-gated ion channel
	Neuronal acetylcholine receptor protein alpha-4 subunit	CHRNA4	Ligand-gated ion channel
	Aminopeptidase A	ENPEP	Protease
	Caspase-2	CASP2	Protease
	Dipeptidyl peptidase IV	DPP4	Protease
	Monoamine oxidase A	MAOA	Other enzyme oxidoreductase
	Monoamine oxidase B	MAOB	Other enzyme oxidoreductase
	NAD-dependent deacetylase sirtuin 1	SIRT1	Other enzyme deacetylase (eraser)
	NAD-dependent deacetylase sirtuin 2	SIRT2	Other enzyme deacetylase (eraser)
	Dopamine transporter (by homology)	SLC6A3	Electrochemical transporter
	Seratonin transporter (by homology)	SLC6A4	Electrochemical transporter
	Cytochrome P450 2A6	CYP2A6	Cytochrome P450
